# Author Correction: Targeted small molecule inhibitors blocking the cytolytic effects of pneumolysin and homologous toxins

**DOI:** 10.1038/s41467-024-48642-1

**Published:** 2024-05-21

**Authors:** Umer Bin Abdul Aziz, Ali Saoud, Marcel Bermudez, Maren Mieth, Amira Atef, Thomas Rudolf, Christoph Arkona, Timo Trenkner, Christoph Böttcher, Kai Ludwig, Angelique Hoelzemer, Andreas C. Hocke, Gerhard Wolber, Jörg Rademann

**Affiliations:** 1 Institute of Pharmacy, Freie Universität Berlin, Königin-Luise-Str. 2+4, 14195 Berlin, Germany; 2 Institute for Pharmaceutical and Medicinal Chemistry, University of Münster, Corrensstr. 48, 48149 Münster, Germany; 3 Department of Infectious Diseases, Respiratory Medicine, and Critical Care, Charité - Universitätsmedizin Berlin, Corporate Member of Freie Universität Berlin and Humboldt-Universität zu Berlin, Berlin, Germany; 4 Department of Medicinal Chemistry, Faculty of Pharmacy, Assuit University, Assiut, 71526 Egypt; 5 Leibniz Institute of Virology, Hamburg, 20251 Germany; 6 Institute of Chemistry and Biochemistry, Research Center of Electron Microscopy (FZEM), Freie Universität Berlin, Fabeckstraße 36A, 14195 Berlin, Germany; 7 First Department of Medicine, University Medical Center Hamburg-Eppendorf (UKE), 20251 Hamburg, Germany

**Keywords:** Target validation, Virtual drug screening, Cryoelectron tomography, Drug discovery and development

Correction to: *Nature Communications* 10.1038/s41467-024-47741-3, published online 26 April 2024

The original version of the Supplementary Information associated with this article contained an error as it was missing two sections, Supplementary Note 1 (Chemical Synthesis) and Supplementary Note 2 (Protein MS Reports). The HTML has been updated to include a corrected version of the Supplementary Information.

### Supplementary information

Updated Supplementary information

