# Supplementary material for: Targeted small molecule inhibitors blocking the cytolytic effects of pneumolysin and homologous toxins
Source: Nat Commun. 2024 Apr 26;15:3537. doi: 10.1038/s41467-024-47741-3 (PMC11053136; doi:10.1038/s41467-024-47741-3)
Supplement: Supplementary file 1 — Supplementary Information [file 41467_2024_47741_MOESM1_ESM.pdf]

Supplementary Information

Targeted Small Molecule Inhibitors Blocking the Cytolytic Effects of Pneumolysin and Homologous Toxins

Contents:

Supplementary Tables 1-3……………………………………….....................................2

Supplementary Figures 1-47…………………………………………………………………..3-52

Supplementary Note 1: Chemical Synthesis……………….…………………………….....53-78

Supplementary Note 2: Protein MS Reports………..…………………………................79-99

Name of the molecule	Specs ID
**VH-1**	AP-970/43375589
**VH-2**	AO-476/43362647
**VH-3**	AG-690/36532030
**VH-4**	AK-918/43077841
**VH-5**	AS-871/42714319
**VH-6**	AN-465/14401023
**VH-7**	AS-871/43476226
**VH-8/PB-1**	AK-968/37129204
**VH-9**	AN-465/43421625
**VH-10**	AF-399/15393031
**PB-1.1**	AE-641/14714337
**PB-1.2**	AG-205/11611396
**PB-1.3**	AG-205/36812063
**PB-1.4**	AG-690/34651017
**PB-1.5**	AG-690/11665897
**PB-2**	AG-690/12889237
**PB-2.13**	AO-299/40799554
**PB-2.14**	AO-299/40799484

Supplementary Table 1. Specs identification numbers of virtual hits (VH), **PB-1** derivatives and **PB-2**.

**Data collection**	
Magnification	28,000
Voltage (kV)	200
Electron exposure ($e^{-}/Å^2$)	180
Defocus value (µm)	-30
Pixel size (Å)	3.73
Dual/Single axis	single
Tilt range	-64°– +64°
Tilt increment	2°
Volta phase plate	yes
**Data processing**	
Software package (version)	FEI Inspect 3D (4.3)
Rebin factor	2
Stack alignment	3 iterations (tilt axis orientation update manually after 2 iterations)
Refinement tracking	Patches (13 x 13)
Reconstruction technique	SIRT (20 iterations)
Voxel size (Å)	7.46

Supplementary Table 2. Cryo-ET data collection and processing

PLY-Variant	Mutation	Primer Sequence 5´ → 3´
PLY-DM	T459G, L460G	CCATTAGCATTTGGGGCACCGGCGGGTATCCCCAGGTAGAAGATAAGG
PLY-D205R	D205R	ACTACACGGTGAGCGTGCGCGCCGTCAAGAATCCGGGC
PLY-E151K	E151K	CCTGCGCGGATGCAGTATAAGAAAATCACGGCGCACAGC
PLY-E151Q	E151Q	CCTGCGCGGATGCAGTATCAGAAAATCACGGCGCACAGC
PLY-T55A	T55A	GCGGATGCAGTATGCAAAAATCACGGCGCACA
PLY-C428A	C428A	CTTGTCAGTCAAAATACGTGAGGCTACGGGGCTGGCGTGGGAATG
PLY-S61A	S61A	AACACCTCTGATATTGCCGTCACCGCCACAAATGATAGC
PLY-N85L	N85L	GACGAGACCCTGCTGGAGCTGAACCCGACCTTGTTAGCG

Supplementary Table 3. Primer sequences used for the generation of PLY mutant variants.

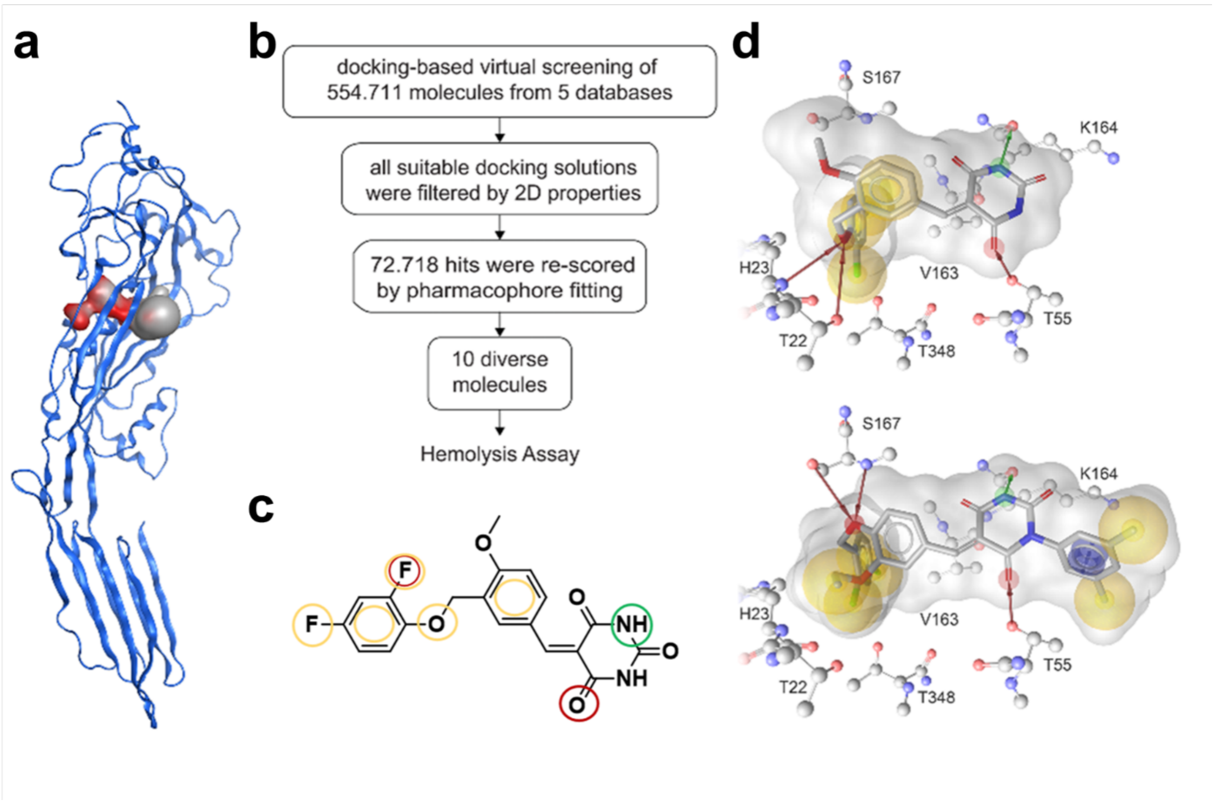

Supplementary Figure 1: Structure-based virtual screening for the identification of PLY inhibitors. (a) The identified potential binding site is shown as surface. **(b)** Virtual screening workflow resulting in 10 diverse hit molecules for testing in a hemolysis assay. **(c)** Molecular structure of **PB-1**, the primary hit compound, with binding interactions predicted by docking. Yellow indicates lipophilic contacts, hydrogen bond acceptors and donors are shown in red and green, respectively. **(d)** Proposed binding modes of **PB-1** (above) and **PB-2** (below) obtained by docking, the color code is the same as in (c), aromatic interactions are shown as purple disk.

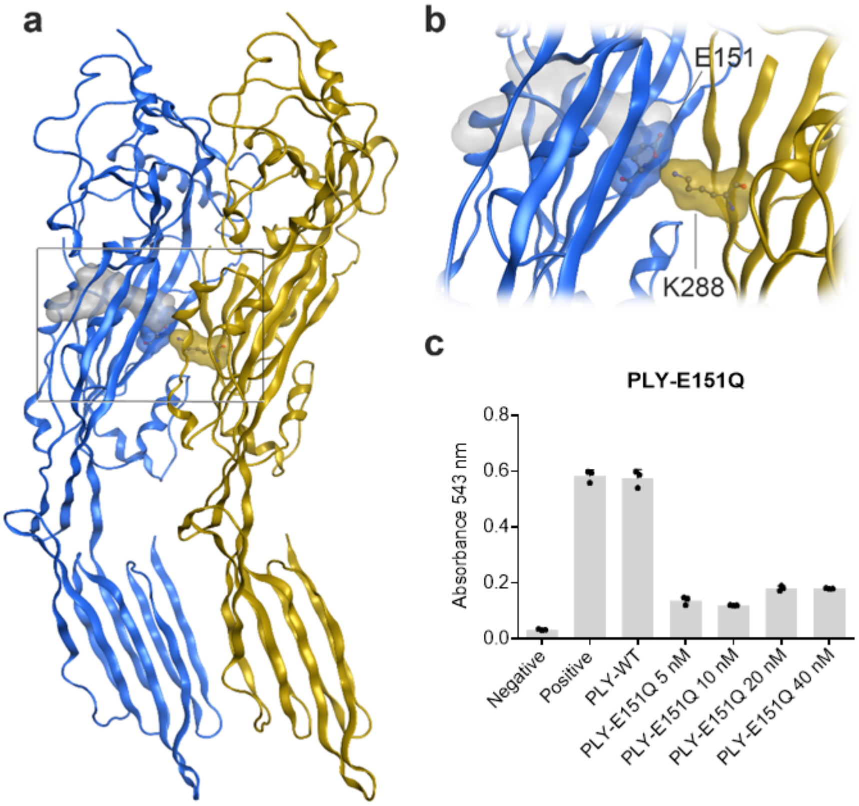

Supplementary Figure 2. (a) The PLY dimer model unveiled a potential binding pocket (grey surface) in close proximity to the dimer interface. **(b)** The salt bridge formed by E151 (blue surface) and K288 (gold surface) of the neighboring PLY monomer at the dimer interface. **(c)** Loss of hemolytic activity in E151 mutant of PLY (PLY-E151Q) proved that PLY-oligomerization is affected (n = 3 and error bars denote ± S.D.).

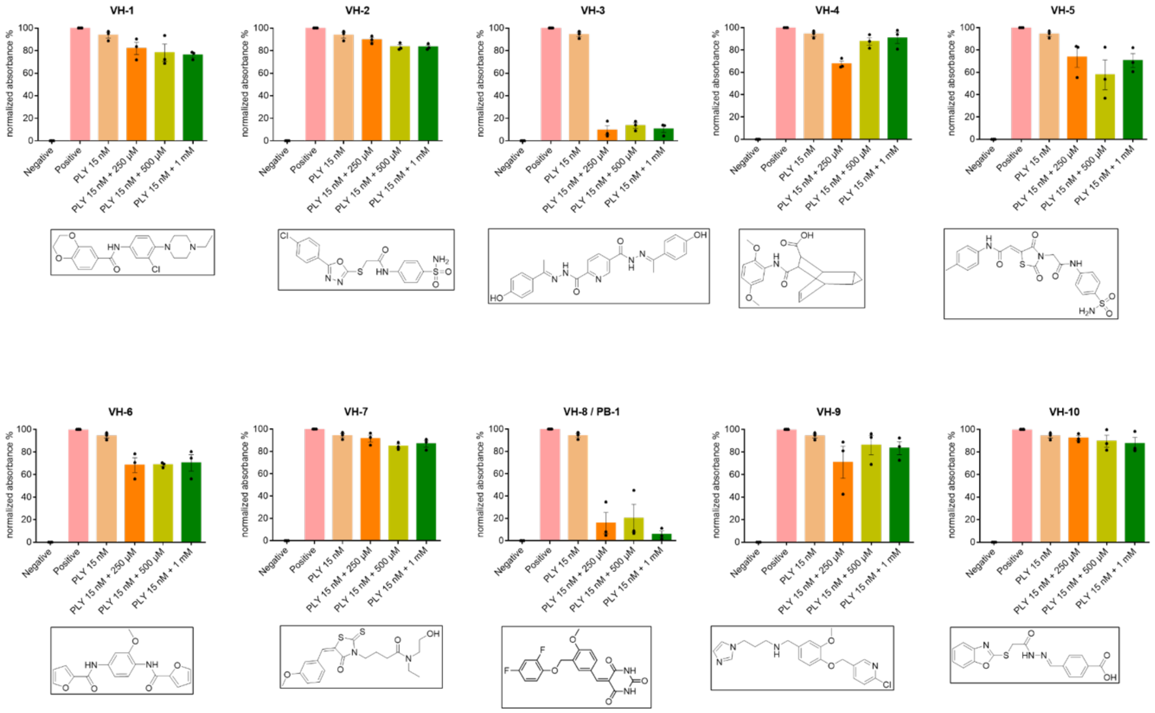

Supplementary Figure 3. Virtual Hits (**VH**) were analyzed at 250, 500 µM and 1 mM concentrations. **VH-3** and **VH-8** / **PB-1** displayed inhibition, though only **VH-8** / **PB-1** was further analyzed while **VH-3** was discontinued due to the poor solubility (n = 3 and error bars correspond to ± S.D.).

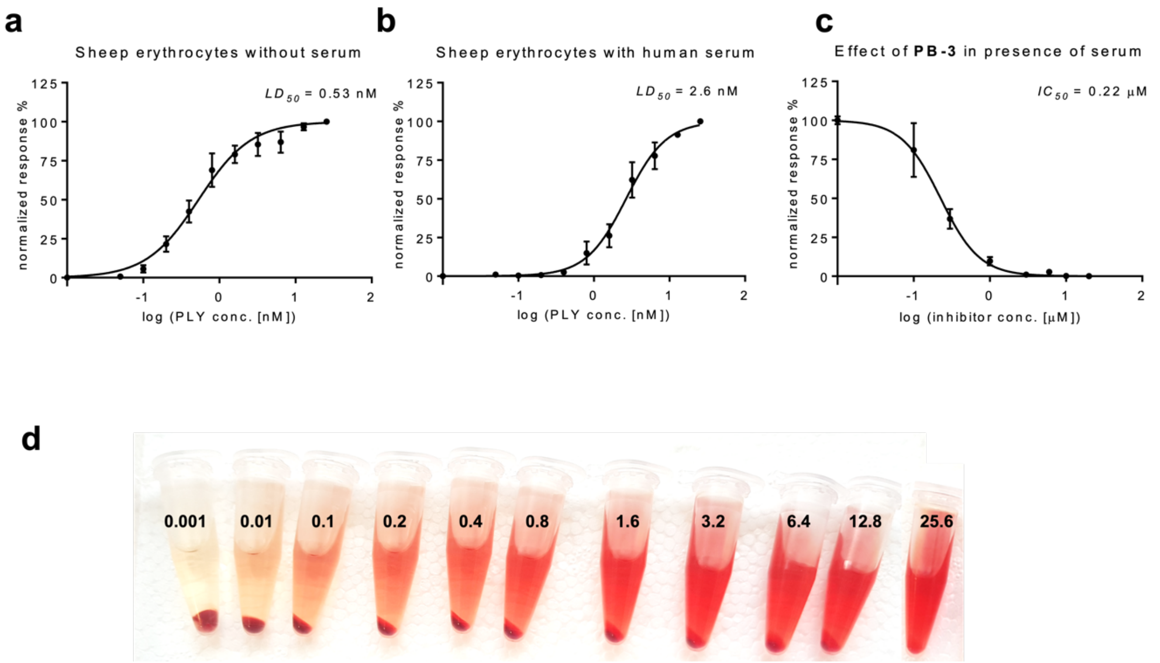

Supplementary Figure 4. Hemolytic activity of recombinant PLY. **(a)** LD_{50} of PLY toward isolated sheep erythrocytes without serum. **(b)** LD_{50} of PLY toward sheep erythrocytes in presence of 2.5% (v/v) serum. **(c)** Inhibition of PLY by **PB-3** in presence of serum. **(d)** The hemolysis of sheep erythrocytes with a gradual increase in concentration (nM) of PLY (n = 3, error bars represent ± S.D.).

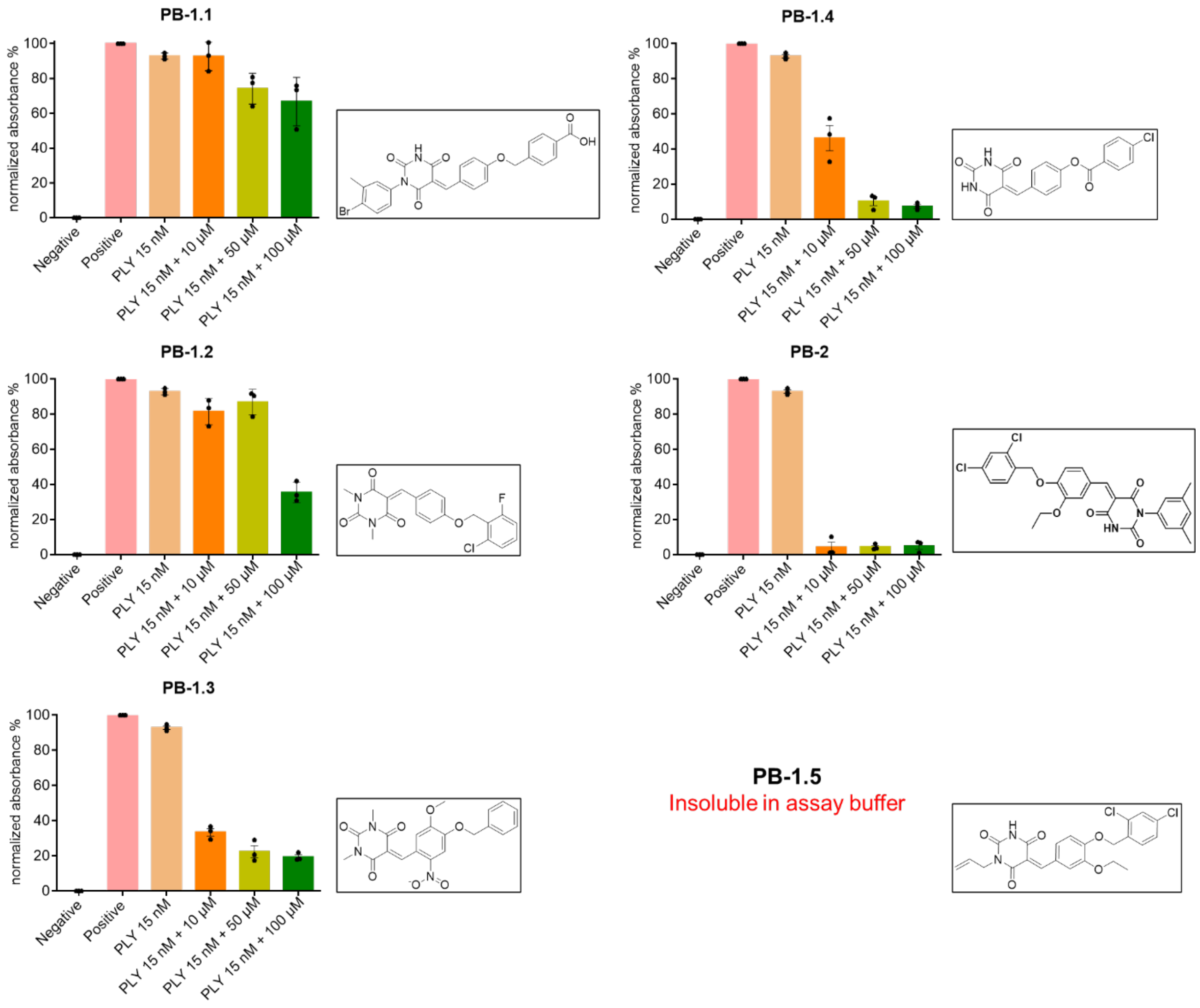

Supplementary Figure 5. Inhibition of PLY in the hemolysis assay using six structural analogs of **PB-1** at concentrations of 10, 50, and 100 μM. Only **PB-2** showed significant inhibition and relatively better solubility. All other molecules were poorly soluble in assay buffer with slight inhibition of PLY, and **PB-1.5** was insoluble (n = 3 and error bars correspond to ± S.D.).

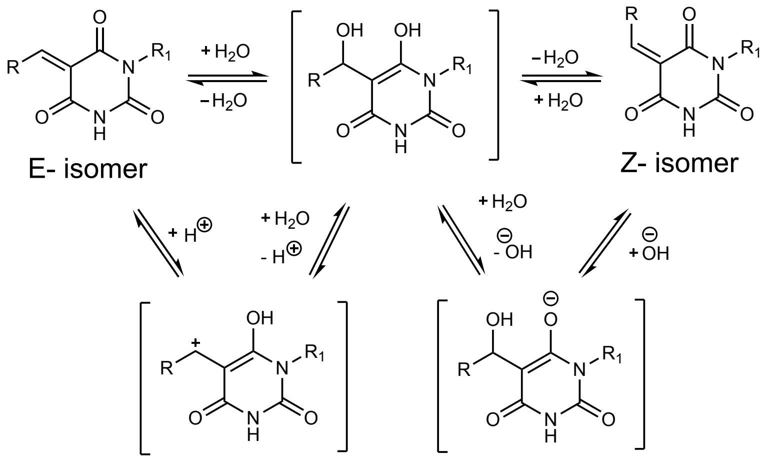

Supplementary Figure 6. Mechanism of the interconversion of E/Z isomers of PB-molecules in water, catalyzed either under acid or basic conditions.

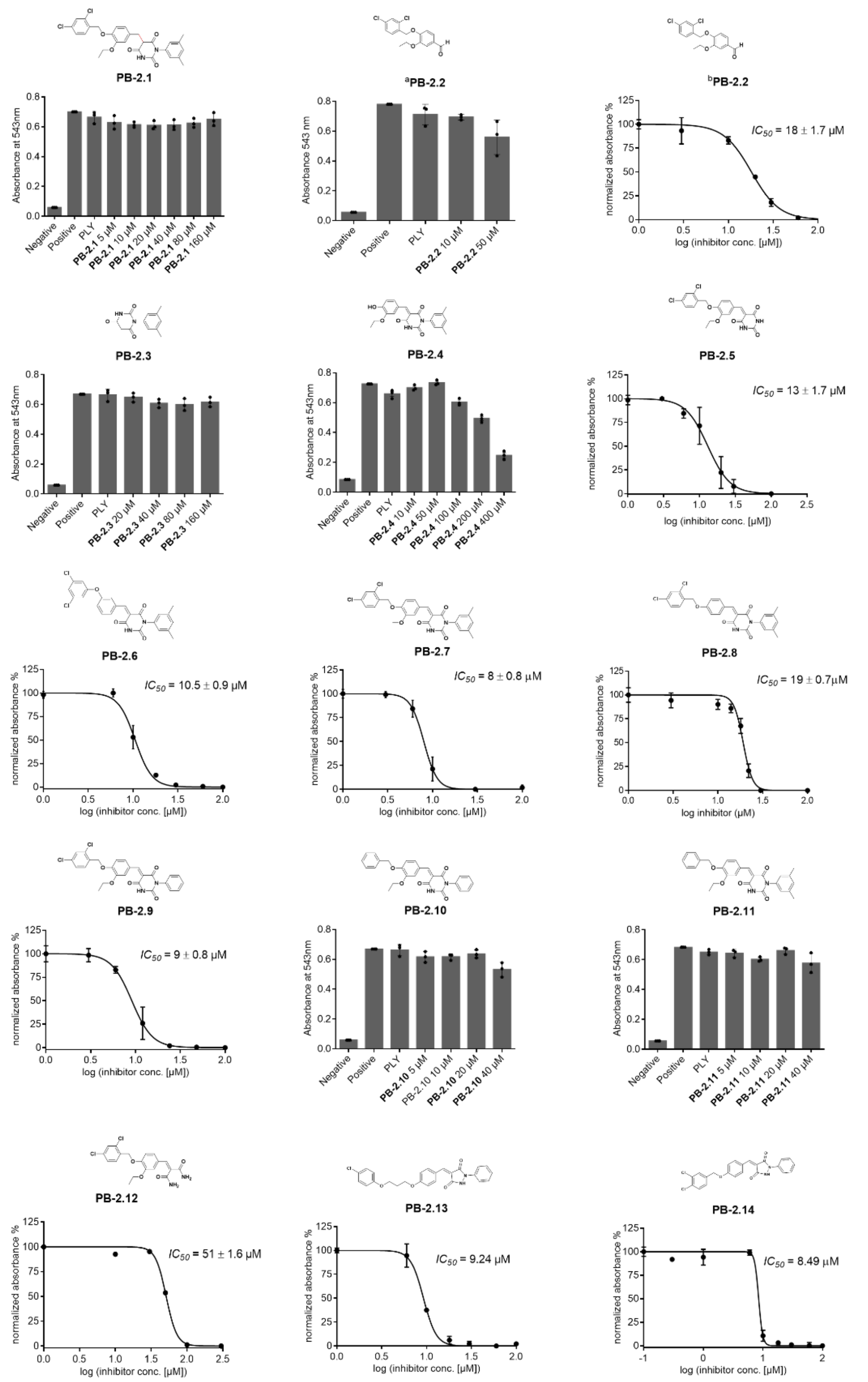

Supplementary Figure 7. Inhibition of PLY by 14 derivatives of **PB-2** in the hemolysis assay, IC_{50} (n = 3, 95% CI and error bars denote ± S.D.) values of active molecules are reported whereas, the effect of inactive molecules are shown as bar graphs (n = 3 and error bars correspond to ± S.D.). [a]**PB-2.2** is inactive after 10 min incubation and [b]**PB-2.2** is active after 1.5 h incubation. Negative controls are given by erythrocytes in assay buffer and positive controls are with 10% Triton X100.

a

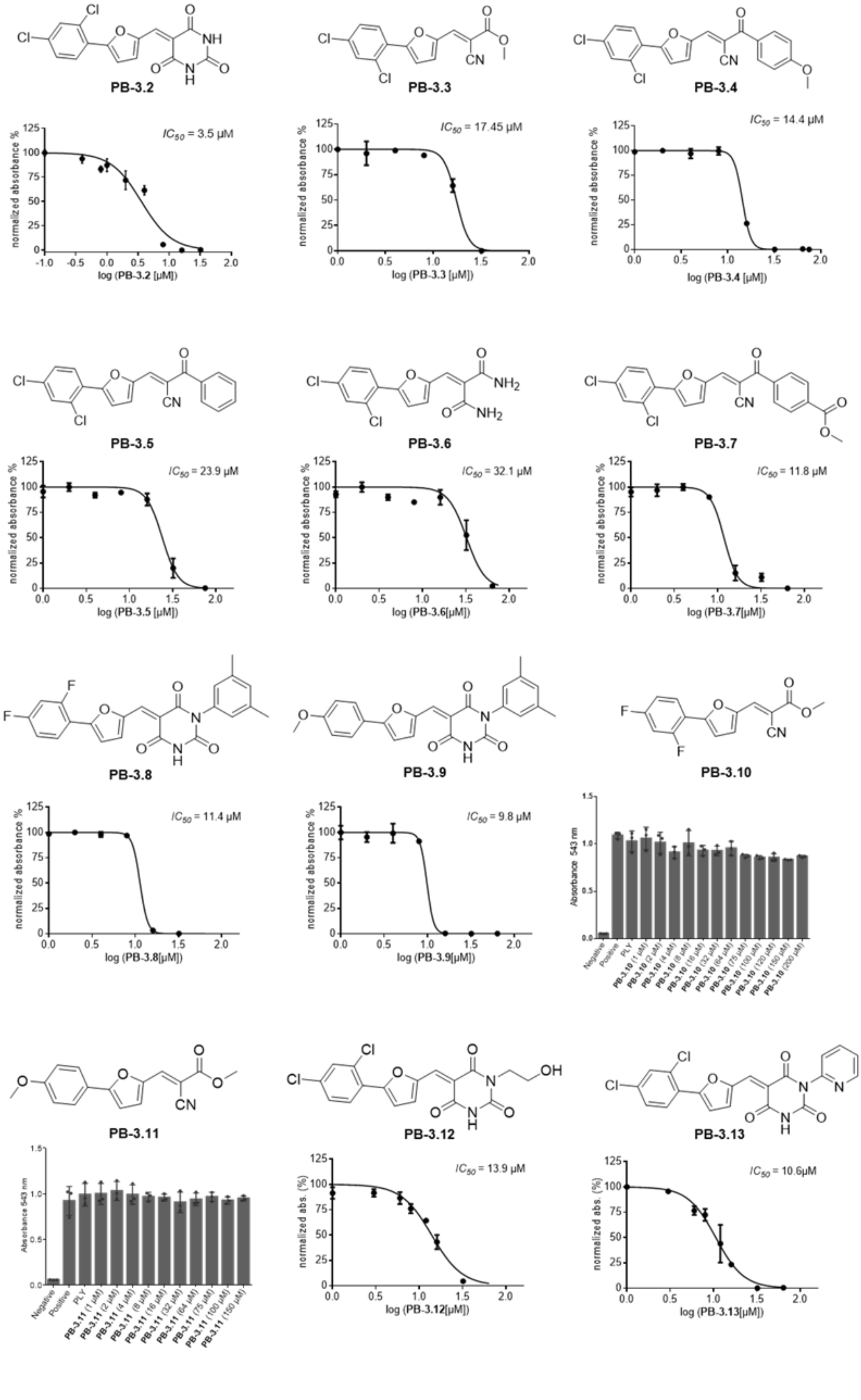
PB-3.2
IC50 = 3.5 µM
normalized absorbance %
log (PB-3.2 [µM])
PB-3.3
IC50 = 17.45 µM
log (PB-3.3 [µM])
PB-3.4
IC50 = 14.4 µM
log (PB-3.4 [µM])
PB-3.5
IC50 = 23.9 µM
log (PB-3.5 [µM])
PB-3.6
IC50 = 32.1 µM
log (PB-3.6[µM])
PB-3.7
IC50 = 11.8 µM
log (PB-3.7[µM])
PB-3.8
IC50 = 11.4 µM
log (PB-3.8[µM])
PB-3.9
IC50 = 9.8 µM
log (PB-3.9[µM])
PB-3.10
Absorbance 543 nm
PB-3.11
PB-3.12
IC50 = 13.9 µM
normalized abs. (%)
log (PB-3.12[µM])
PB-3.13
IC50 = 10.6µM
log (PB-3.13[µM])

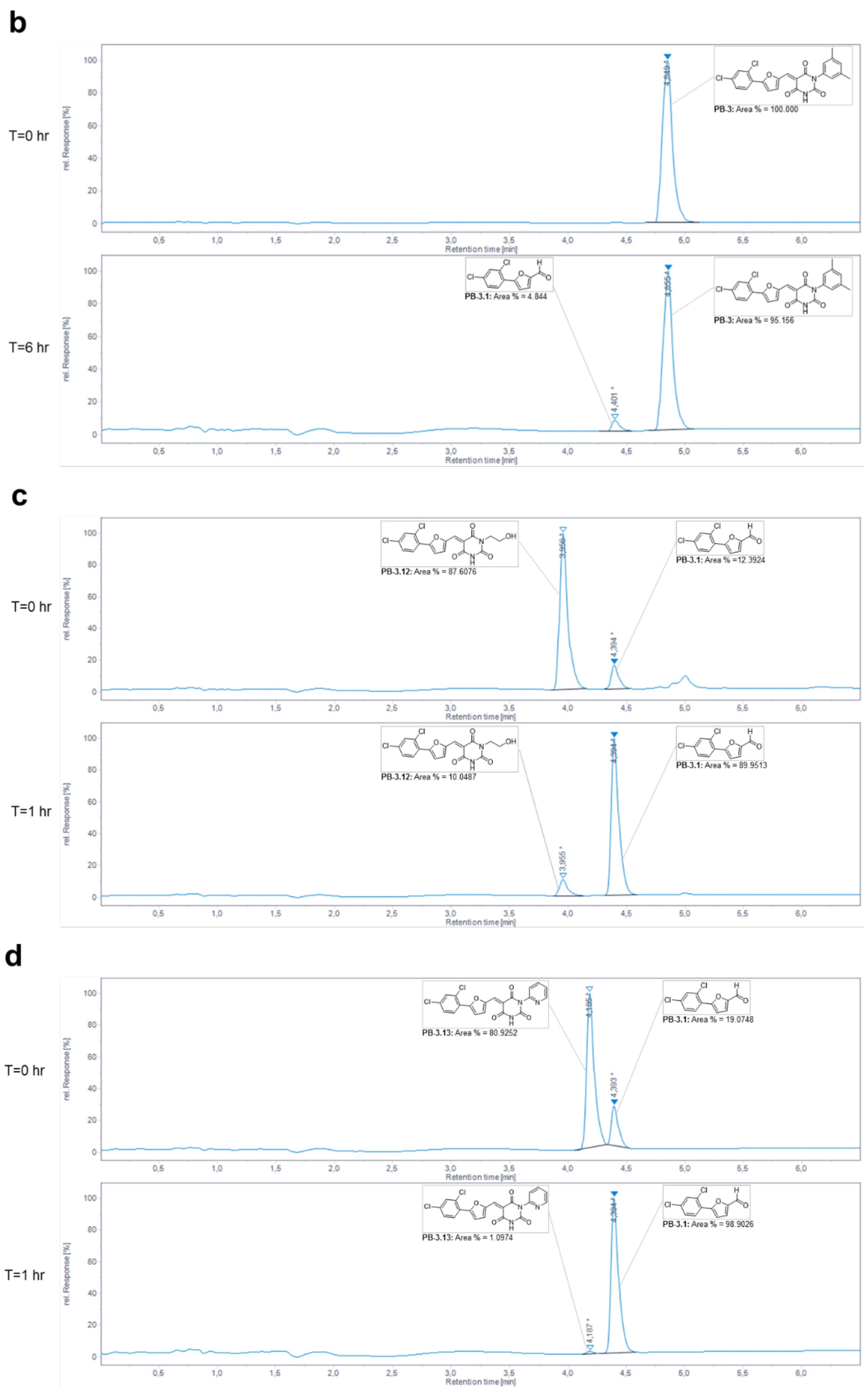

Supplementary Figure 8. (a) Inhibition of PLY by 13 derivatives of **PB-3** molecules in the hemolysis assay, IC_{50} (n = 3, 95% CI and error bars denote ± S.D.). **(b, c, d)** Chemical stability of **PB-3**, **PB-3.12**, and **PB-3.13**, respectively. Stability was determined by HPLC at detector wavelength of 356 nm after incubating the inhibitor at 37° C in PBS for a specified time interval (0 and 6 h for **PB-3**) and (0 and 1 h for **PB-3.12** and **PB-3.13**).

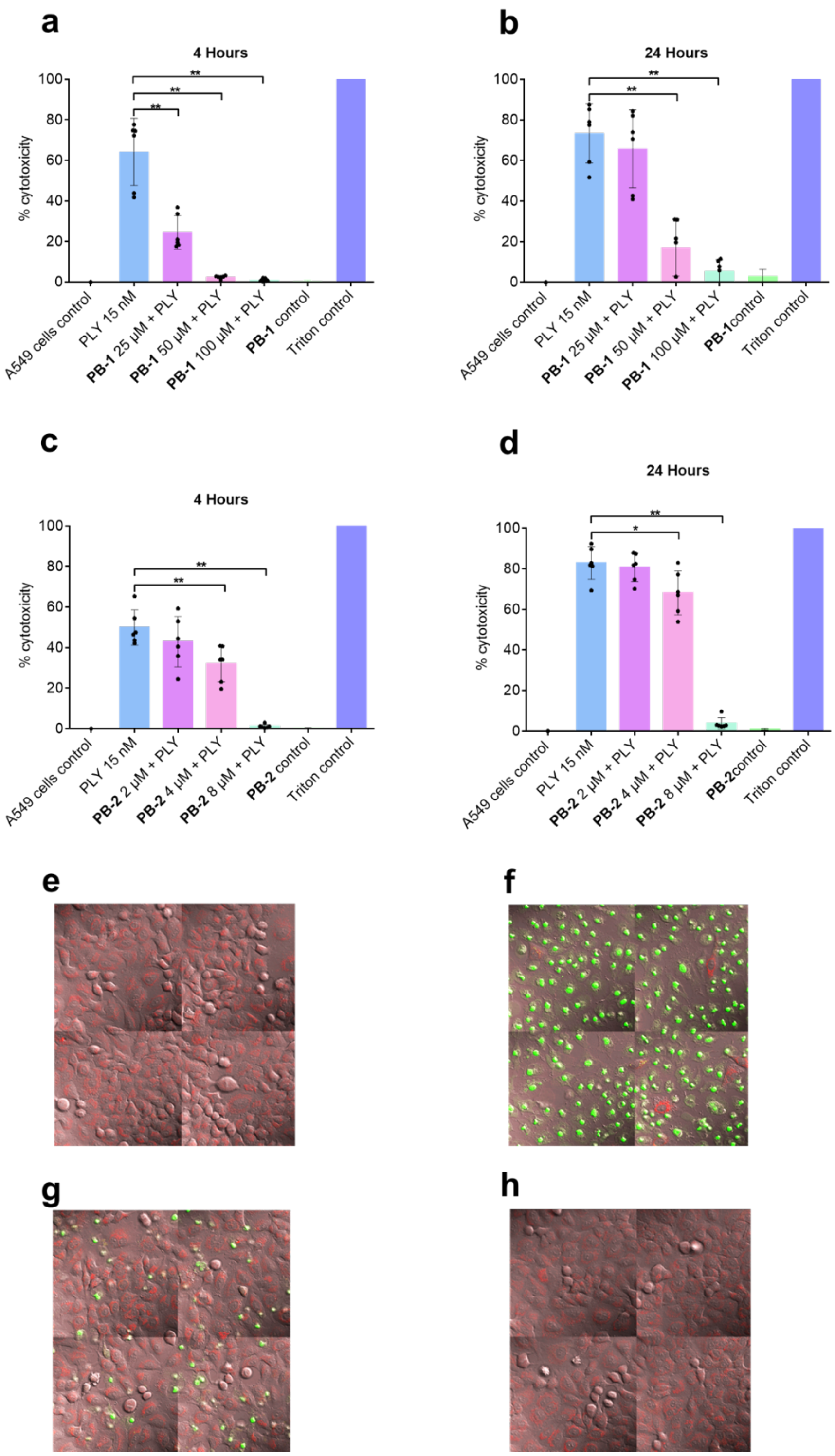

Supplementary Figure 9. LDH assays and Microscopy: (a, b, c & d) PB-1 and **PB-2** prevented human alveolar epithelial cells from PLY-associated impairment. Samples included controls, PLY with three concentrations of inhibitors and PLY alone. Cells were incubated with samples and the cytotoxicity was calculated at two time points (4 h and 24 h). Cytotoxicity of samples was quantified by the LDH release of each respective sample. (n ≥ 5, *p < 0.05, **p < 0.001 and error bars represents ± S.D.). **PB-2** impeded PLY-mediated cellular injury in A549 cells (human alveolar epithelial cells). Cells were grown in Ham's F12 culture medium with inclusion of a red mitochondrial dye

(TMRE: tetramethyl rhodamine, ethyl ester), as indicative of healthy cells and a green fluorescent substrate which highlighted caspase-3/7 activity in damaged cells. **(e)** Healthy cells as a negative control (24 h). **(f)** Cells stimulated with PLY 15 nM (24 h). **(g)** Cells treated with PLY 15 nM + **PB-2** 8 μM (24 h). **(h)** Cells with only **PB-2** as a control (24 h). All images were recorded using a Zeiss LSM780 confocal laser-scanning microscope after 24 h (data shown in e, f, g and h are one of n = 3).

a

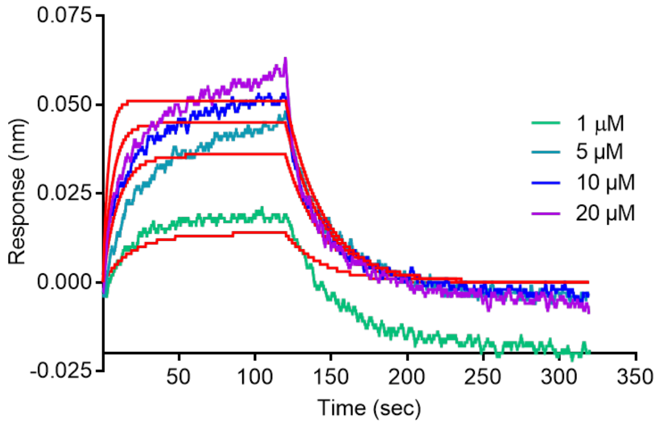

b

Inhibitor	k_{on} ($M^{-1}s^{-1}$)	k_{on} Error	k_{off} (s^{-1})	k_{off} Error	K_D (M)	K_D Error	*Chi*^{2}	Full R^2
**PB-2**	1.19×10^4	7.16×10^2	4×10^{-2}	1.47×10^{-3}	3.37×10^{-6}	2.38×10^{-7}	0.0744	0.8789

Supplementary Figure 10. (a, b) Affinity between **PB-2** and PLY-WT was calculated using four concentrations in the BLI assay. The reference-subtracted data are presented in the graph, which shows association and dissociation of **PB-2** with PLY-WT (data shown is one of n = 3). For quantitative analysis, the one-to-one kinetic fitting model was applied.

Particle Sizing Systems, Inc.
Santa Barbara, Calif., USA

DOPC: CHOL 70:30

INTENSITY-Weighted GAUSSIAN DISTRIBUTION Analysis (Solid Particle)

GAUSSIAN SUMMARY:

Mean Diameter	*= 101.7 nm*	*Variance (P.I.)*	*= 0.074*
Stnd. Deviation	*= 27.7 nm (27.2%)*	*Chi Squared*	*= 0.552*
Norm. Stnd. Dev.	*= 0.272*	*Baseline Adj.*	*= 0.038 %*
(Coeff. of Var'n)		*Z-Avg. Diff. Coeff.*	*= 4.57E-008 cm2/s*

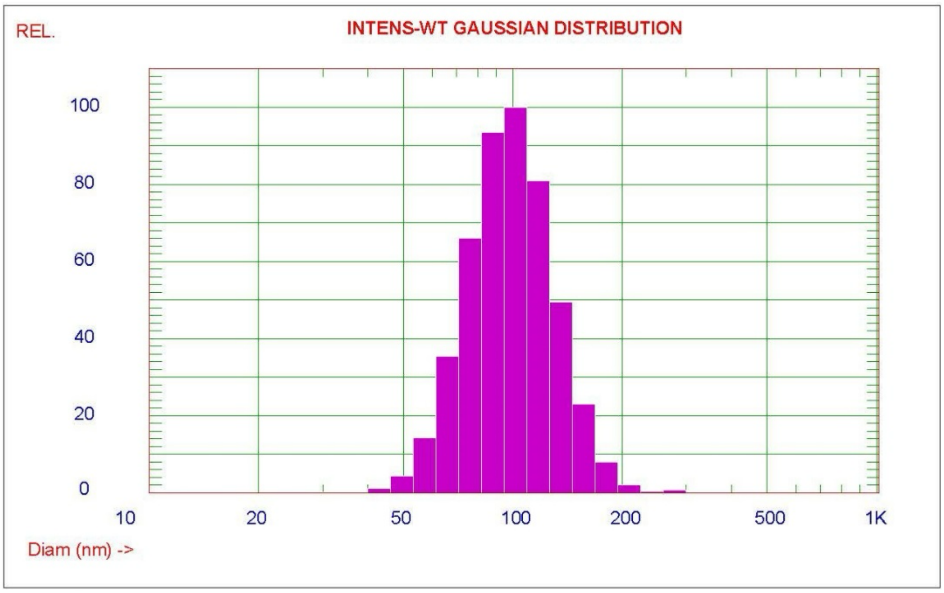

Run_Sample

Cumulative Result:

25 % of distribution < 83.4 nm
50 % of distribution < 101.7 nm
75 % of distribution < 120.5 nm
90 % of distribution < 141.3 nm
99 % of distribution < 186.6 nm
80 % of distribution < 125.9 nm

Run Time	*= 0 Hr 9 Min 27 Sec*	*Wavelength*	*= 632.8 nm*
Count Rate	*= 304 KHz*	*Temperature*	*= 23 deg C*
Channel #1	*= 523.0 K*	*Viscosity*	*= 0.933 cp*
Channel Width	*= 12.0 uSec*	*Index of Ref.*	*= 1.333*

Supplementary Figure 11. Dynamic light scattering (DLS) analysis of liposomes (data here is one representative of n = 3).

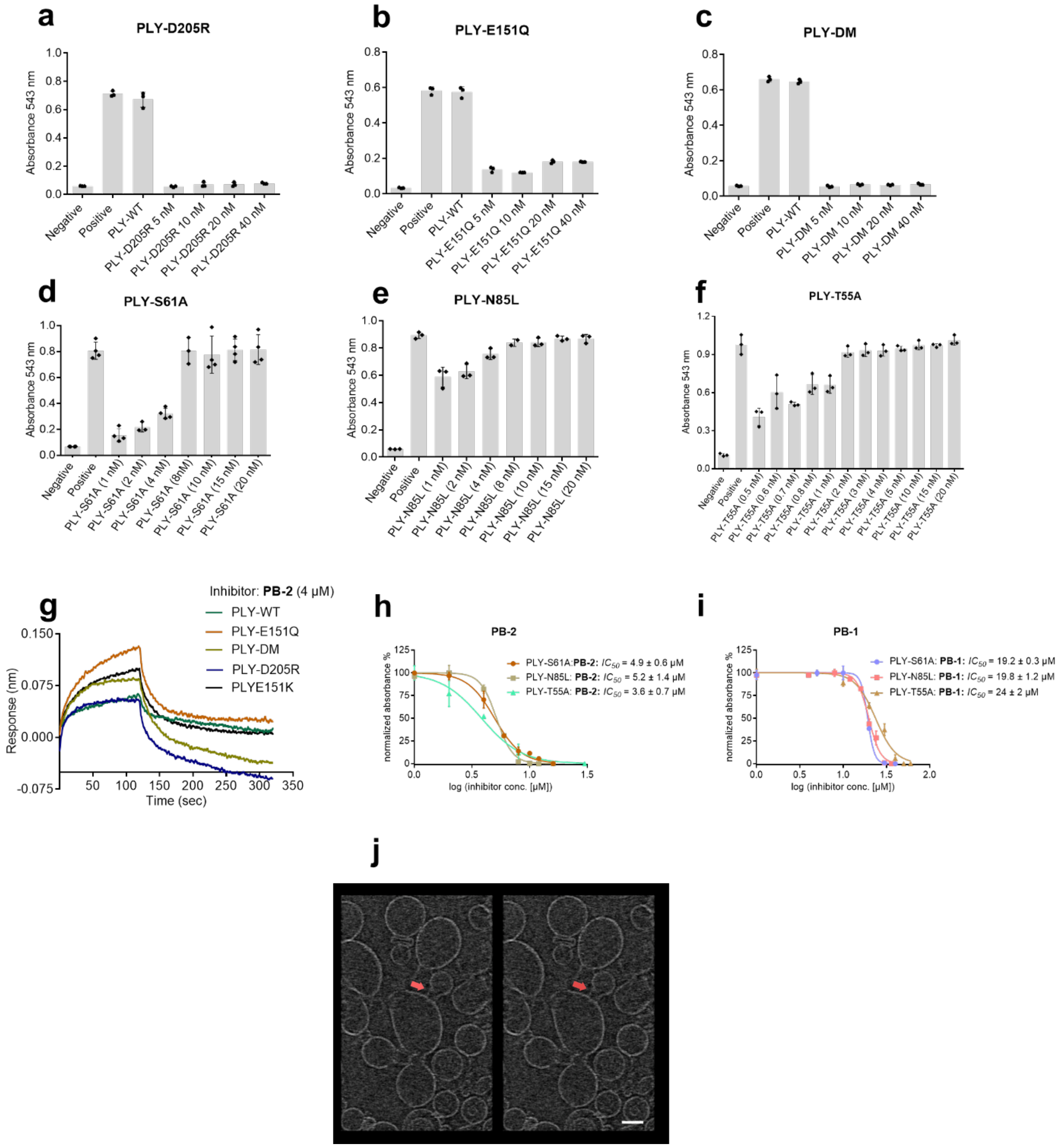

Supplementary Figure 12. Hemolytic analysis of PLY mutants in comparison to wild type PLY. **(a)** PLY-D205R, **(b)** PLY-E151Q, and **(c)** PLY-DM all displayed minimal hemolytic activity. **(d)** PLY-S61A, **(e)** PLY-N85L, and **(f)** PLY-T55A all exhibited hemolytic activity. (**a** to **f**, n = 3 and error bars represents ± S.D.) **(g)** Binding of **PB-2** (4 μM) to PLY-WT, PLY-E151Q, PLY-E151K, PLY-DM and PLY-D205R as recorded in the bio-layer interferometry assay (data shown is one of n = 3). The association of **PB-2** (4 μM) with all variants of PLY confirmed that the activity mechanism of **PB-2** inhibition did not depend on these mutated amino acids. **(h)** IC_{50} of **PB-2** against PLY-S61A, PLY-N85L, and PLY-T55A. **(i)** IC_{50} of **PB-1** against PLY-S61A, PLY-N85L, and PLY-T55A. (**h** and **i**, n = 3 and error bars represents ± S.D.) **(j)** Reconstructed 3D volume of a cryo-electron tomography of liposomes in the presence of PLY-WT, shown in *voltex* presentation as a side-by-side stereogram. The arrows mark examples of PLY-induced pre-pore and pore-formation.

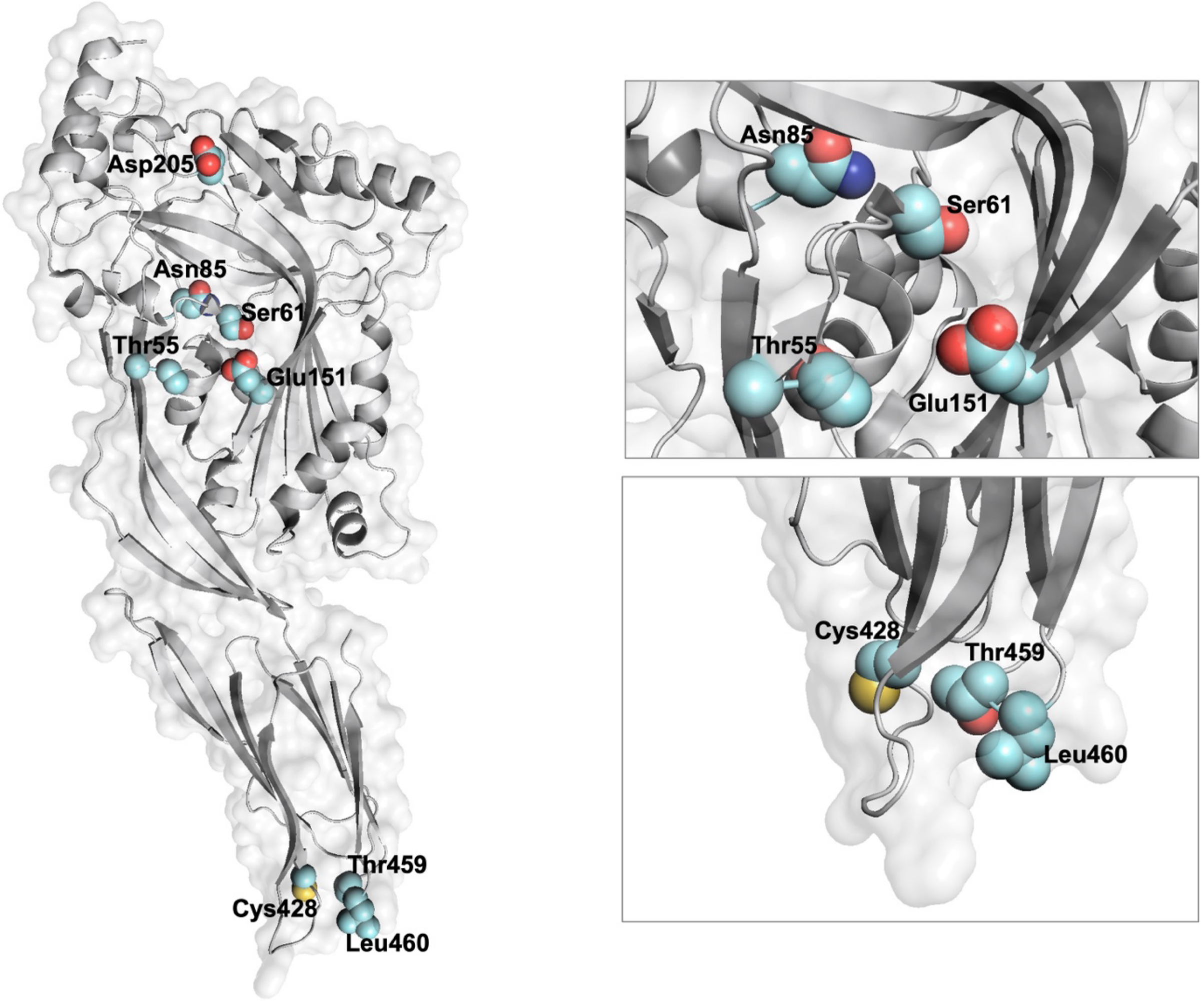

Supplementary Figure 13. For identification of the binding site of **PB-1-3**, eight mutants of PLY were generated by site-directed mutagenesis involving the eight amino acids indicated here, in the oligomerization domain (Thr55, Ser61, Asn85, Glu 151, and Asp205) or in the cholesterol binding domain (Cys428, Thr459, and Leu 460.

a

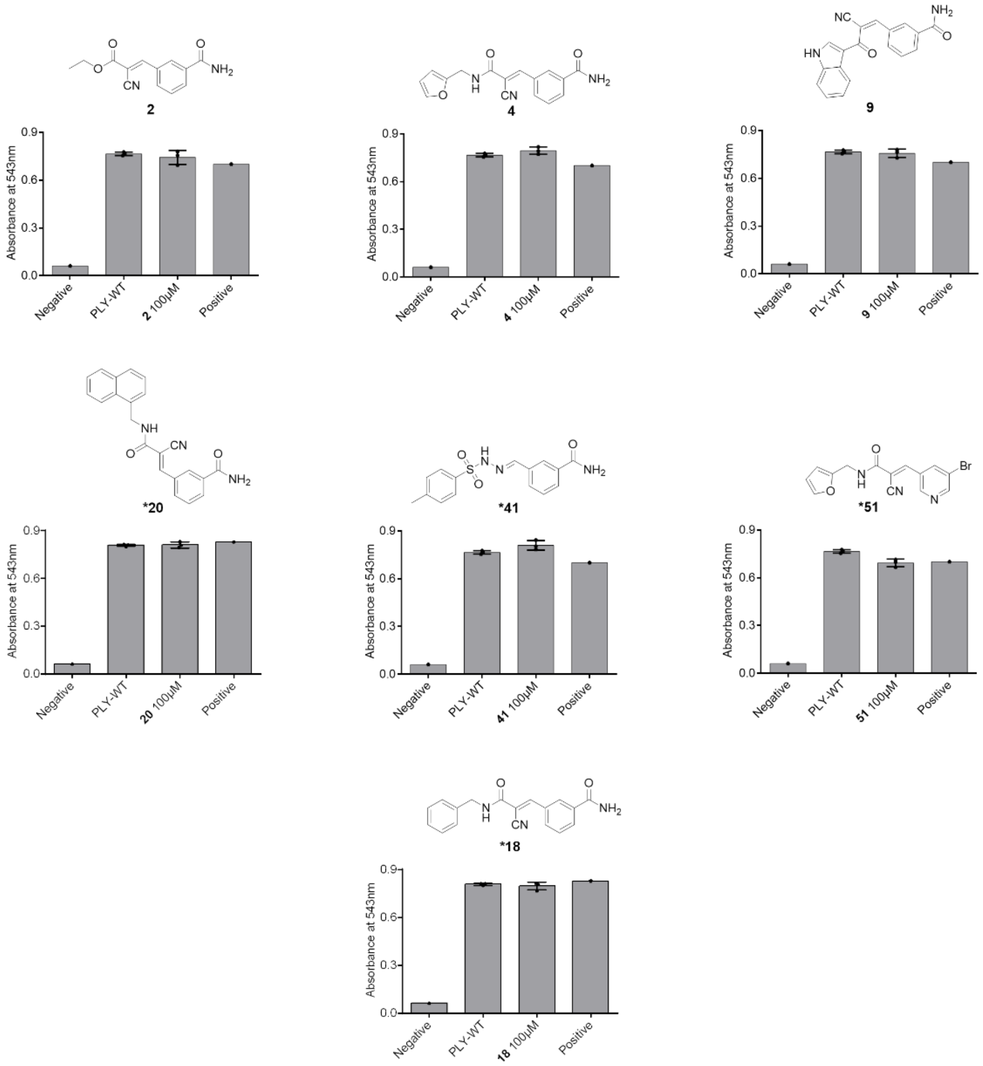
2
4
9
*20
*41
*51
*18
Absorbance at 543nm
0.0
0.3
0.6
0.9
Negative
PLY-WT
2 100μM
4 100μM
9 100μM
20 100μM
41 100μM
51 100μM
18 100μM
Positive

b

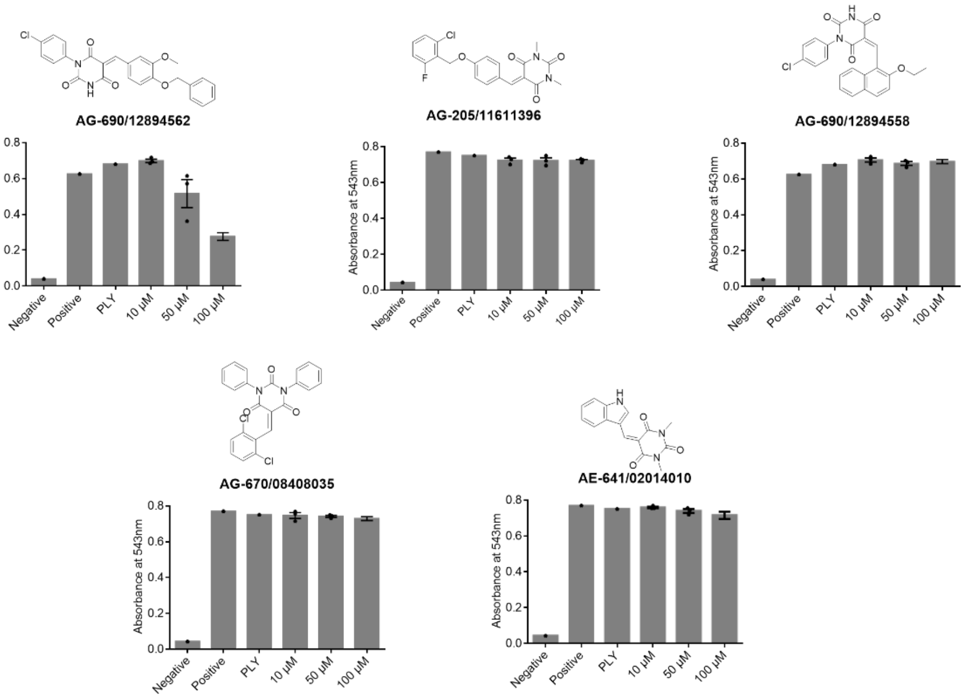
AG-690/12894562
AG-205/11611396
AG-690/12894558
AG-670/08408035
AE-641/02014010
Absorbance at 543nm
0.0
0.2
0.4
0.6
0.8
Negative
Positive
PLY
10 μM
50 μM
100 μM

c

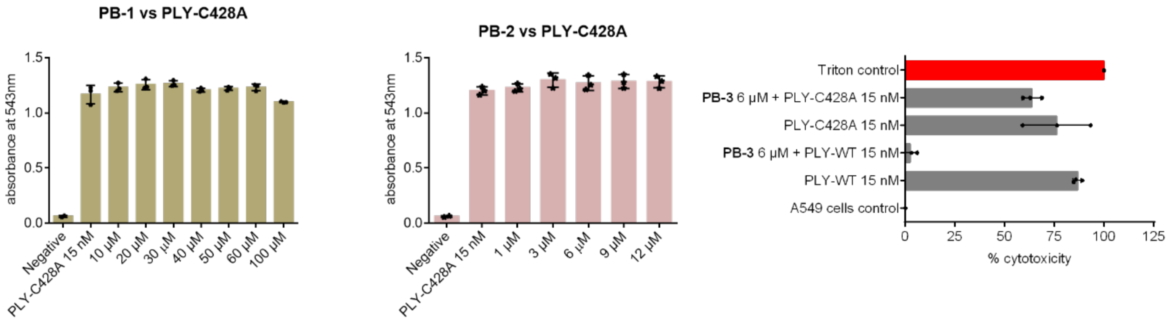

d

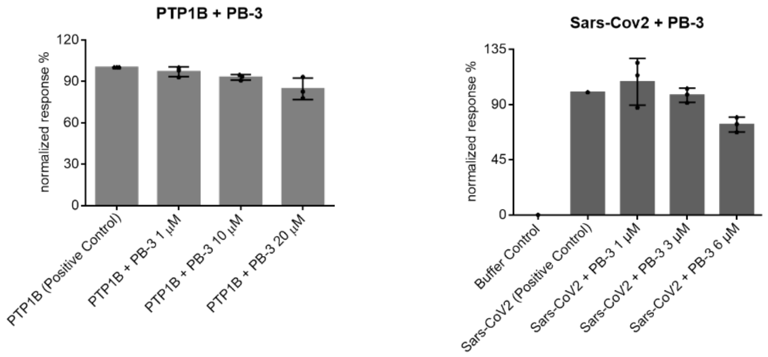

Supplementary Figure 14. Specificity of **PB-3** for PLY. **(a)** Seven Michael acceptors reported as inhibitors of EV D68 protease do not inhibit PLY (n ≥ 3, error bars represent ± S.D, * means not published yet). **(b)** Five alternative and commercially available Michael acceptors are unable to block PLY (n = 3, error bars represent ± S.D, molecules purchased from Specs and compound ID is the title of graph). **(c) PB-1** and **2** are inactive against PLY-C428A in the hemolysis assay. **PB-3** (6 µM) is inactive against PLY-C428A in LDH assay (n ≥ 3 for all experiments, error bars represent ± S.D). **(d) PB-3** is inactive against the cysteine protease of SARS-CoV-2 and the protein tyrosine phosphatase PTP1B, **PB-3** is soluble in assay buffer up to measured concentrations beyond that **PB-3** precipitates (n = 3, error bars represent ± S.D).

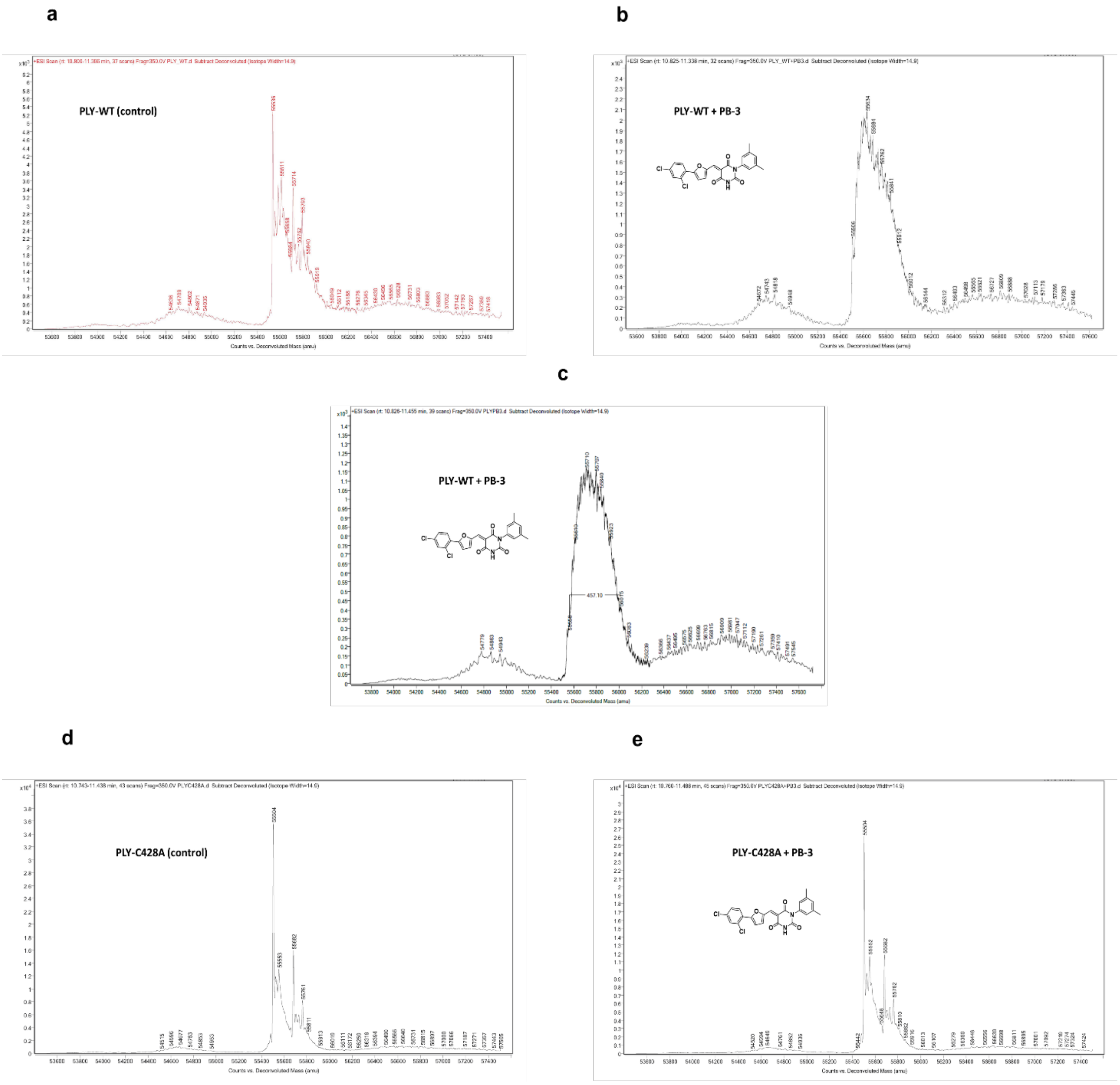

Supplementary Figure 15. MS spectra of PLY-WT and PLY-C428A with and without **PB-3** in denaturing conditions (data shown are one representative of n = 3). **(a)** PLY-WT alone sample, the main peak matches protein mass. **(b)** PLY-WT with **PB-3** exhibited broadening of the protein-peak. **(c)** The width of broad protein-ligand peak is in the range of inhibitor mass. **(d, e)** PLY-C428A control and PLY-C428A with **PB-3** exhibited identical spectra, which suggested no binding interaction between PLY-C428A and **PB-3**

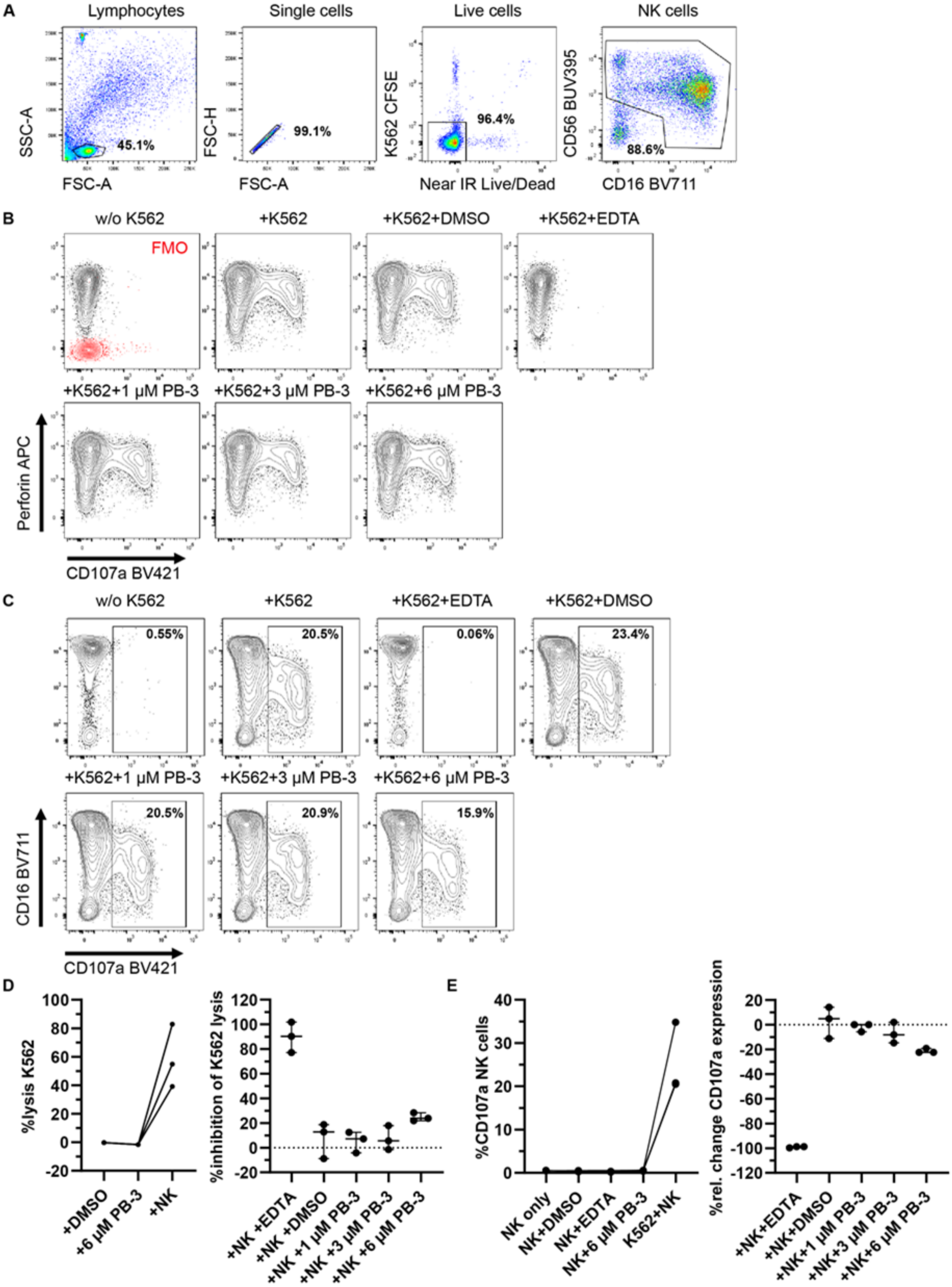
A
Lymphocytes
Single cells
Live cells
NK cells
SSC-A
FSC-A
45.1%
FSC-H
FSC-A
99.1%
K562 CFSE
Near IR Live/Dead
96.4%
CD56 BUV395
CD16 BV711
88.6%
B
w/o K562
FMO
+K562
+K562+DMSO
+K562+EDTA
+K562+1 µM PB-3
+K562+3 µM PB-3
+K562+6 µM PB-3
Perforin APC
CD107a BV421
C
w/o K562
0.55%
+K562
20.5%
+K562+EDTA
0.06%
+K562+DMSO
23.4%
+K562+1 µM PB-3
20.5%
+K562+3 µM PB-3
20.9%
+K562+6 µM PB-3
15.9%
CD16 BV711
CD107a BV421
D
%lysis K562
+DMSO
+6 µM PB-3
+NK
%inhibition of K562 lysis
+NK +EDTA
+NK +DMSO
+NK +1 µM PB-3
+NK +3 µM PB-3
+NK +6 µM PB-3
E
%CD107a NK cells
NK only
NK+DMSO
NK+EDTA
NK+6 µM PB-3
K562+NK
%rel. change CD107a expression
+NK+EDTA
+NK+DMSO
+NK+1 µM PB-3
+NK+3 µM PB-3
+NK+6 µM PB-3

Supplementary Figure 16. NK cell cytotoxicity assay against K562 to test effect of **PB-3** on human perforin. Flow cytometric assessment of NK cell degranulation and killing of K562 target cells after 3h co-culture at E:T ratio of 2:1. **(a)** Gating strategy to identify NK cells. Gate was set on lymphocytes and doublets were excluded. Live cells, which were negative for CFSE, were gated to exclude K562 cells. CD56 and CD16 were used to identify NK cells. **(b)** Exemplary dot plots depict intracellular perforin content (y-axis) in degranulating NK cells (as measured by CD107a, x-axis) with or without indicated concentrations of **PB-3**. As vehicle control, DMSO was used; 10 mM of EDTA served as positive control of NK cell inhibition. Fluorescence-minus-one (FMO) control for perforin staining overlaid in red. **(c)** Exemplary dot plots depict NK cell degranulation in absence and presence of K562 cells with or without indicated concentrations of **PB-3**. As vehicle control, DMSO was used; 10 mM of EDTA served as positive control of NK cell inhibition. **(d)** The percentage of lysed K562 cells is plotted following co-incubation with only DMSO, in the presence of 6 µM of **PB-3** or with NK cells (left). %lysis is calculated as: 100- (K562 survived (absolute count)/K562 alone (absolute count) x 100). Scatter plot illustrates the percentage of inhibition of K562 lysis by tested concentrations of **PB -3** and controls compared to untreated NK cells (right). Dots represent mean values of two technical replicates per donor for lysis (left). **(e)** The percentage of CD107a+ cells of bulk NK cells without K562, with the respective controls or in the presence of K562 cells (left). Scatter plots display the relative change in degranulation in the presence of **PB-3** at indicated concentrations compared to untreated NK cells (right). The percentage of inhibition was calculated using the formula [(K562 + NK cells) - (K562 Condition + NK cells)] / (K562 + NK cells) x 100. Relative degranulation was calculated using the formula [(K562 Condition + NK cells) - (K562 + NK cells)] / (K562 + NK cells) x 100. Black bar represents median with interquartile range. N = 3 individual NK cell donors.

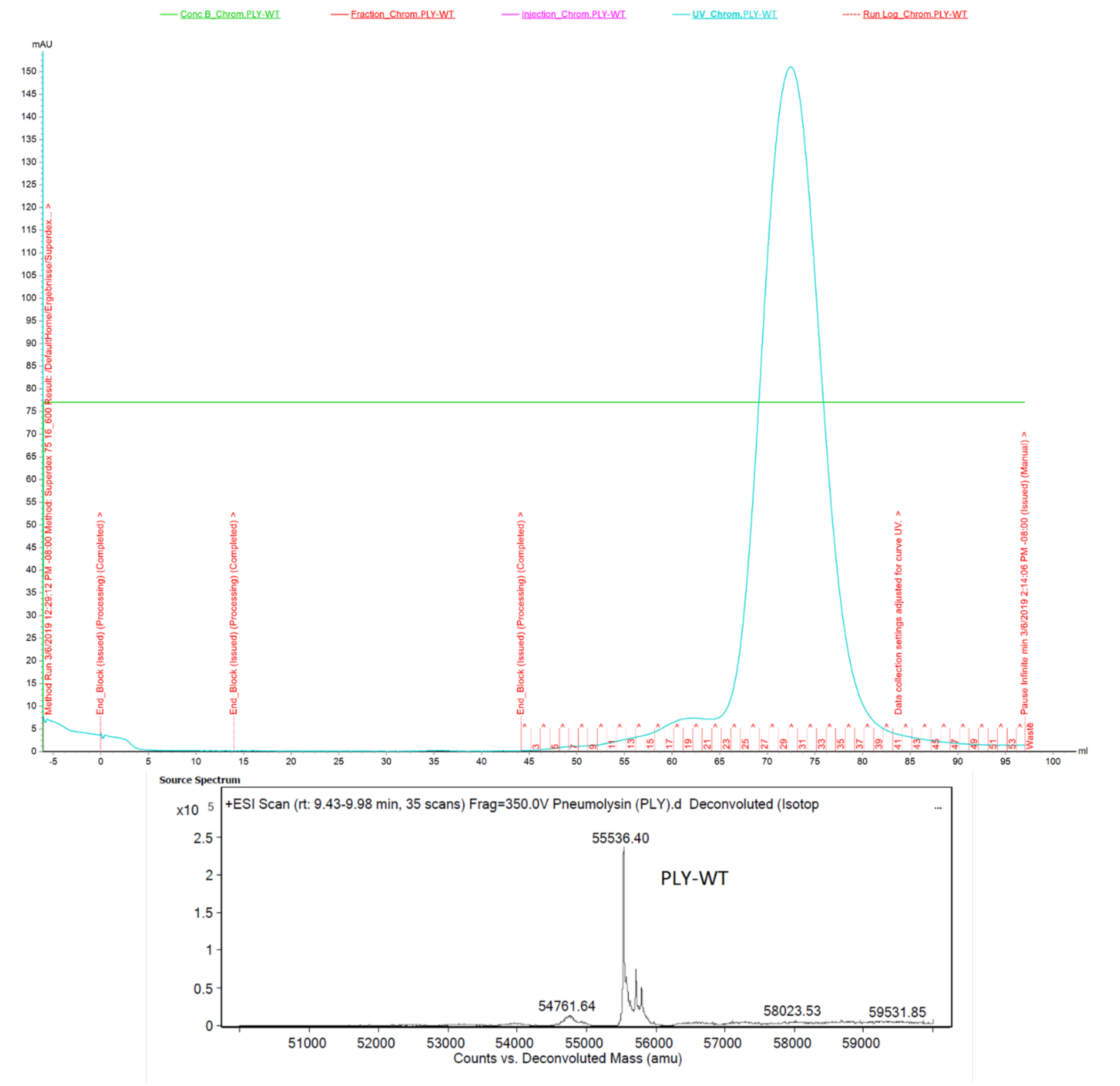

Supplementary Figure 17. Size exclusion chromatogram of recombinant pneumolysin (PLY) on HiLoad 16/600 Superdex 75 pg column and deconvoluted protein mass spectrum displaying the protein mass peak.

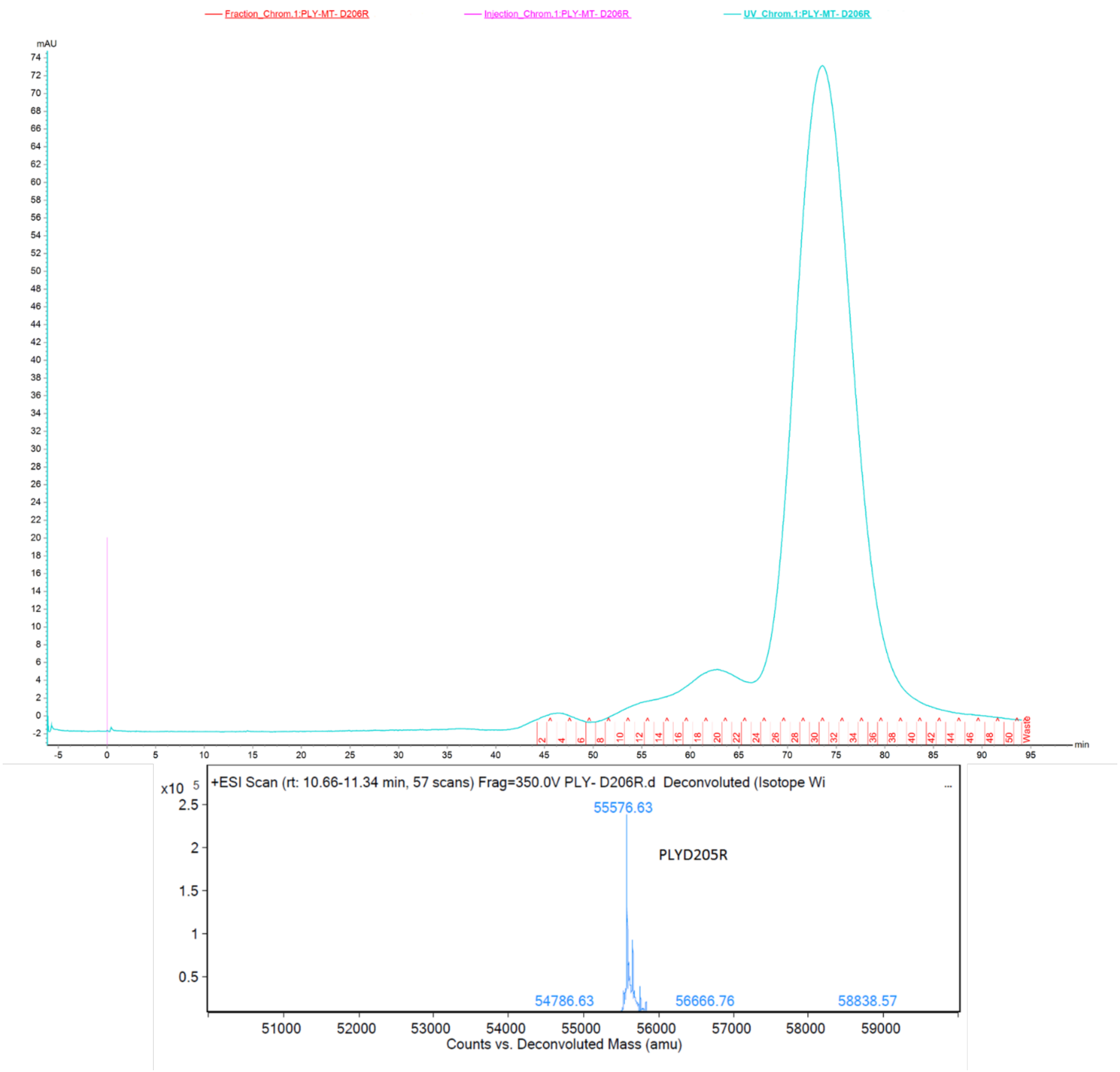

Supplementary Figure 18. Size exclusion chromatogram of PLY-D205R on HiLoad 16/600 Superdex 75 pg column and deconvoluted protein mass spectrum displaying the protein mass peak.

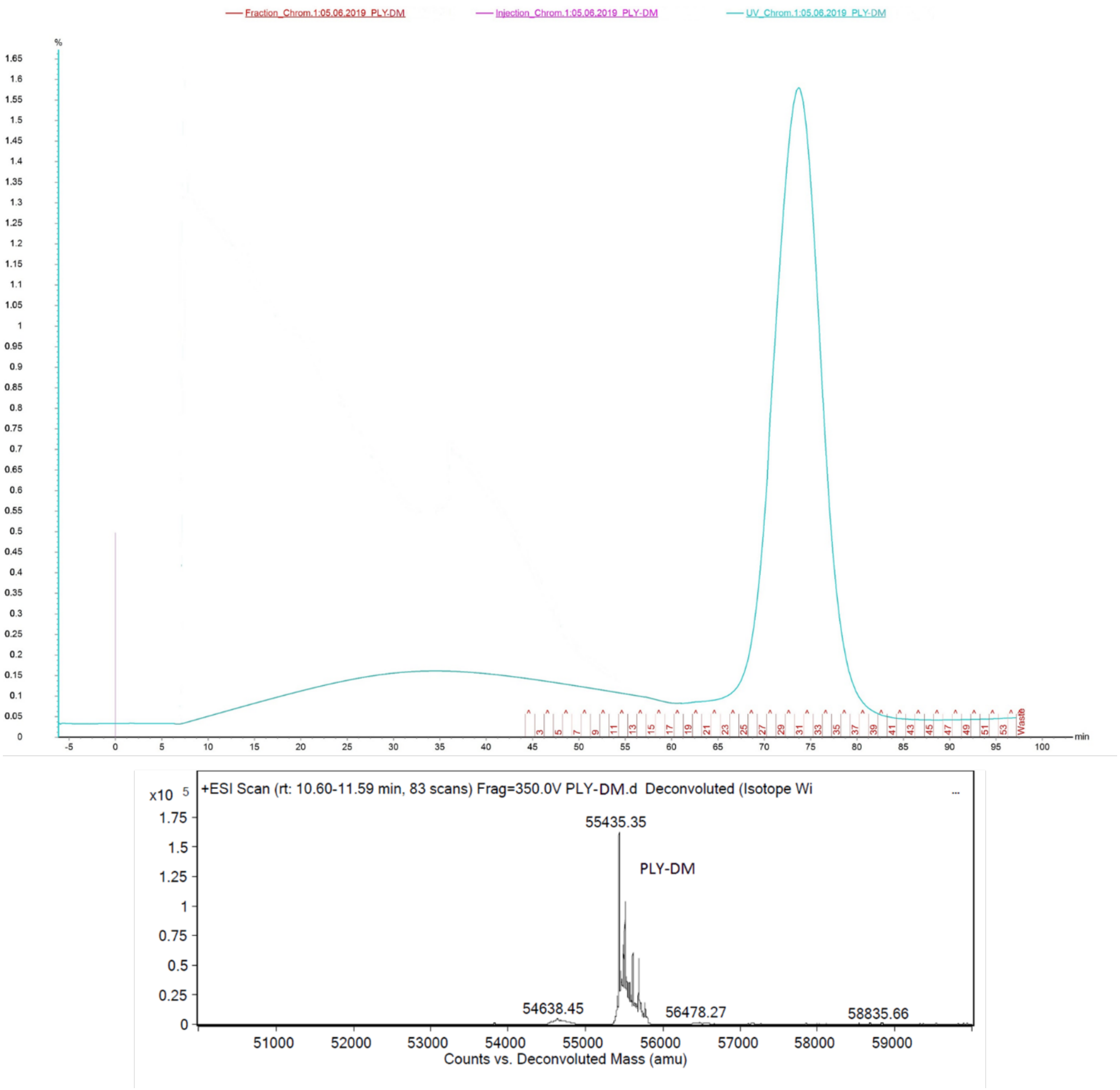

Supplementary Figure 19. Size exclusion chromatogram of pneumolysin double mutant (PLY-DM) on HiLoad 16/600 Superdex 75 pg column and deconvoluted protein mass spectrum displaying the protein mass peak.

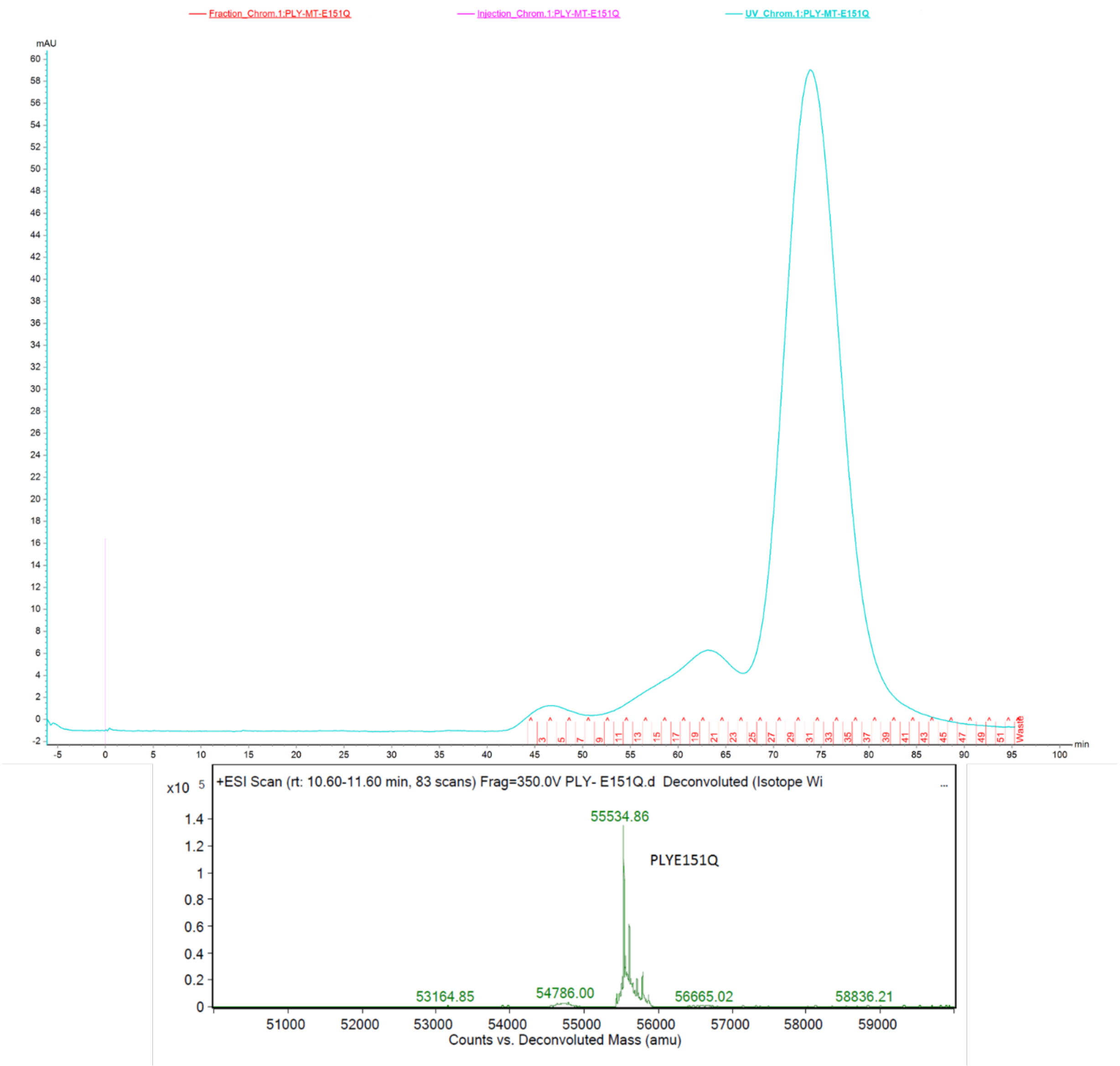

Supplementary Figure 20. Size exclusion chromatogram of PLY-E151Q on HiLoad 16/600 Superdex 75 pg column and deconvoluted protein mass spectrum displaying the protein mass peak.

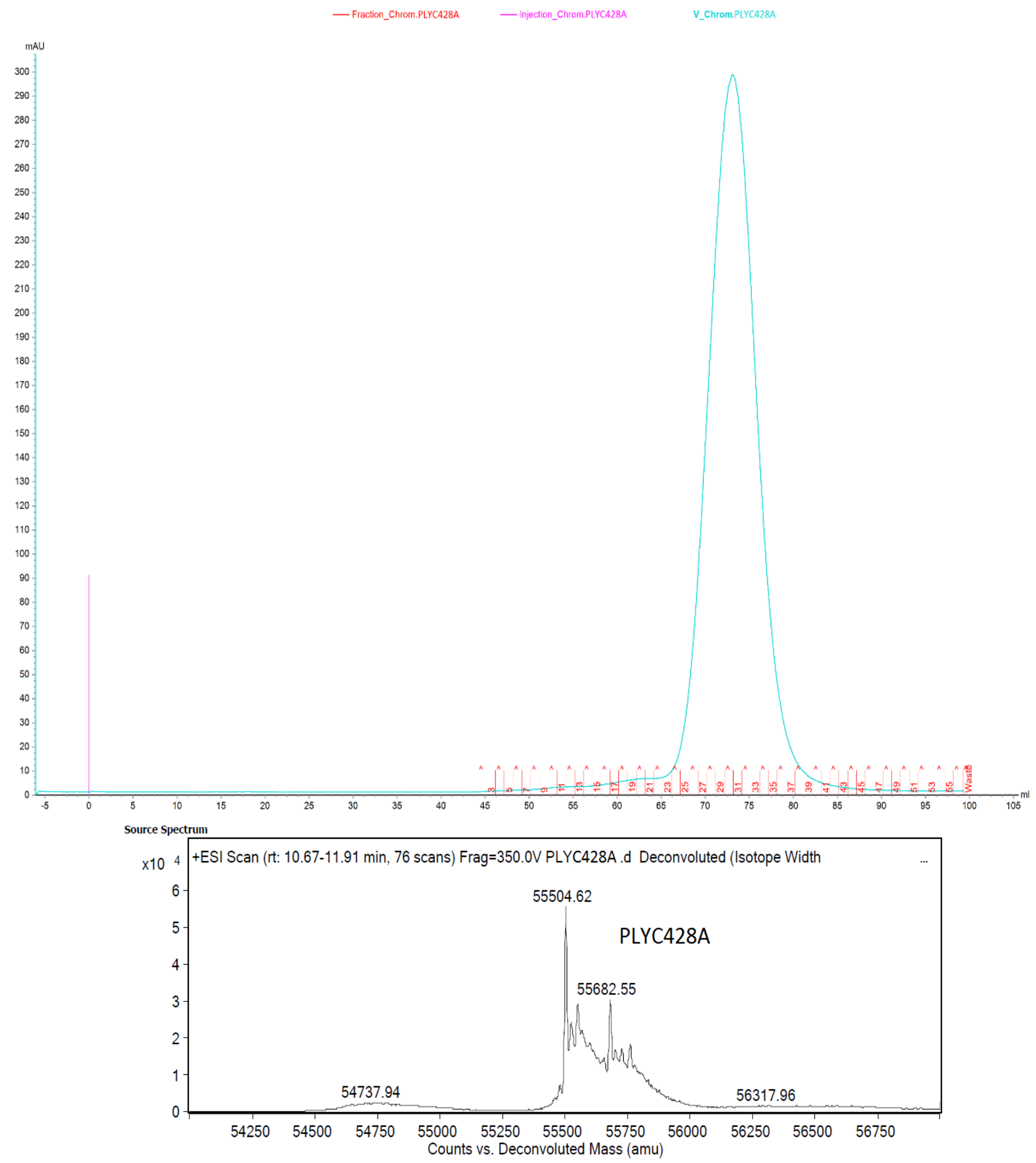

Supplementary Figure 21. Size exclusion chromatogram of PLY-C428A on HiLoad 16/600 Superdex 75 pg column and deconvoluted protein mass spectrum displaying the protein mass peak.

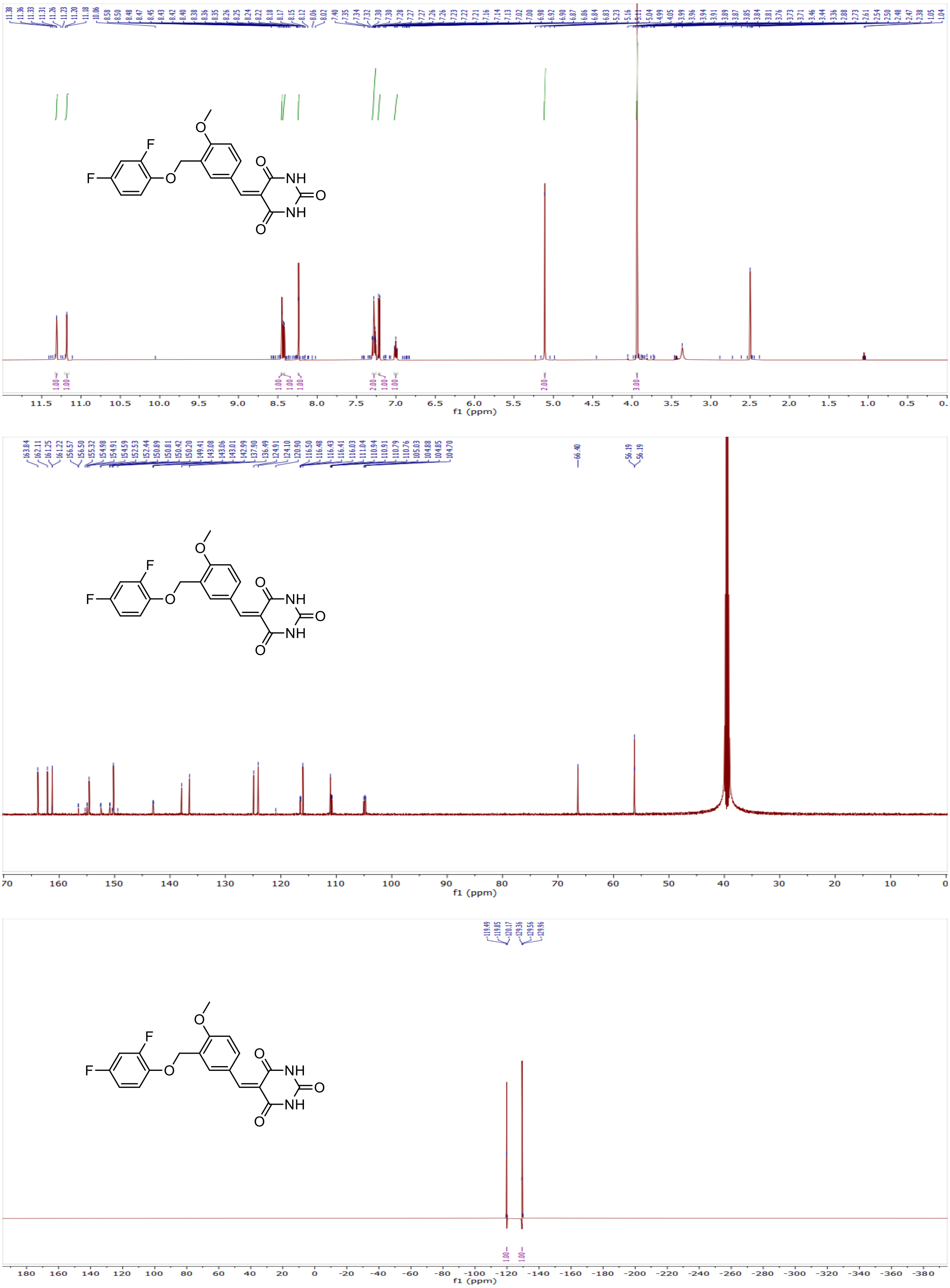

Supplementary Figure 22. ^{1}H-NMR (600 MHz, DMSO-d$_6$) (top), ^{13}C-NMR (151 MHz, DMSO-*d*$_6$) (middle) and ^{19}F-NMR (565 MHz, DMSO-d$_6$) (bottom) spectrum of **PB-1**.

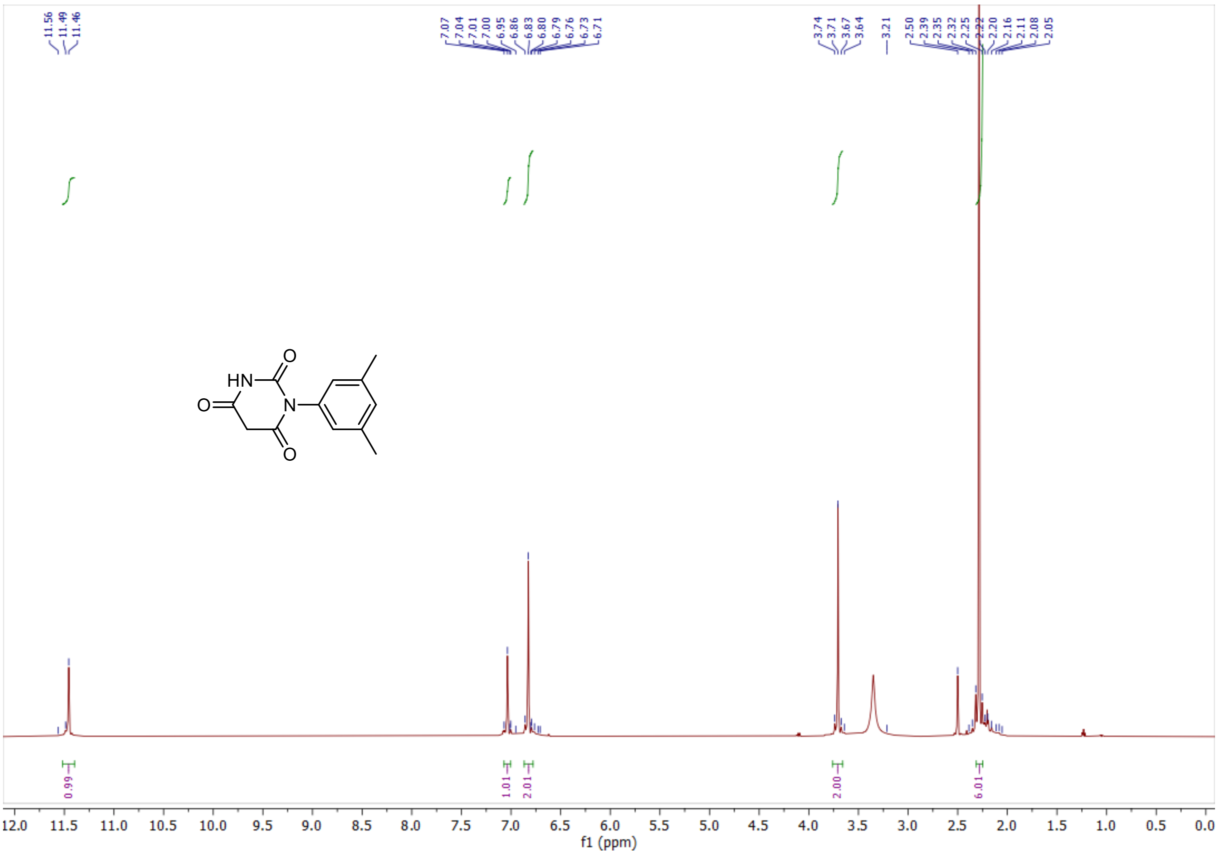

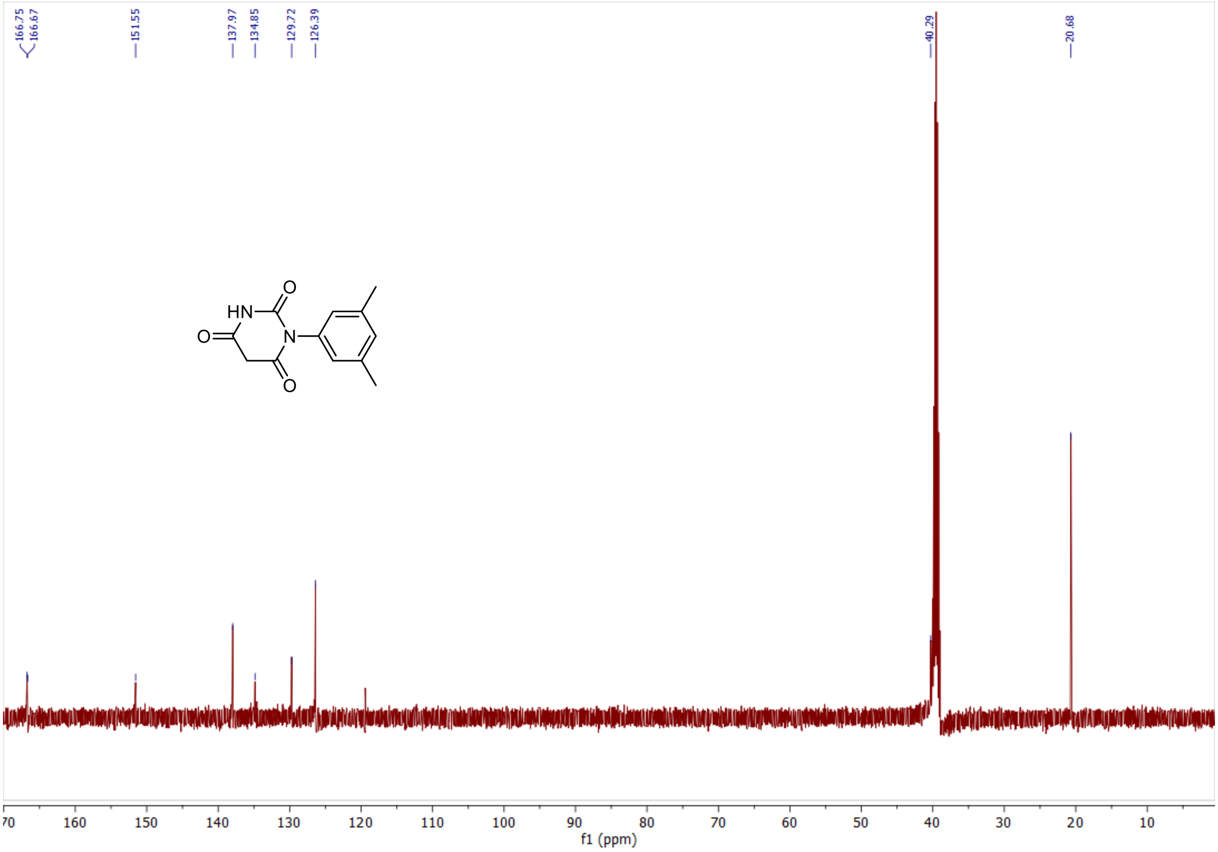

Supplementary Figure 23. ^{1}H-NMR (500 MHz, DMSO-d$_6$) and ^{13}C-NMR (126 MHz, DMSO-*d*$_6$) spectrum of **PB-2.3**.

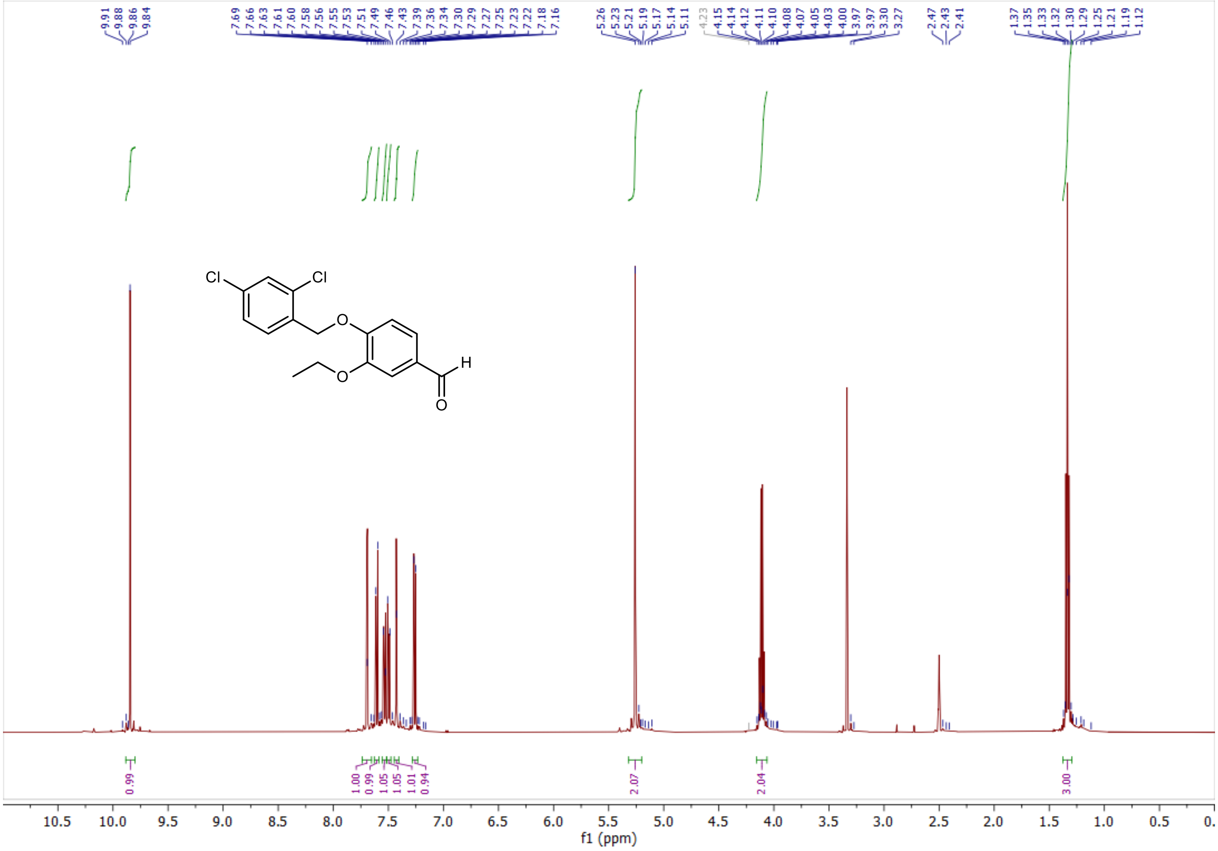

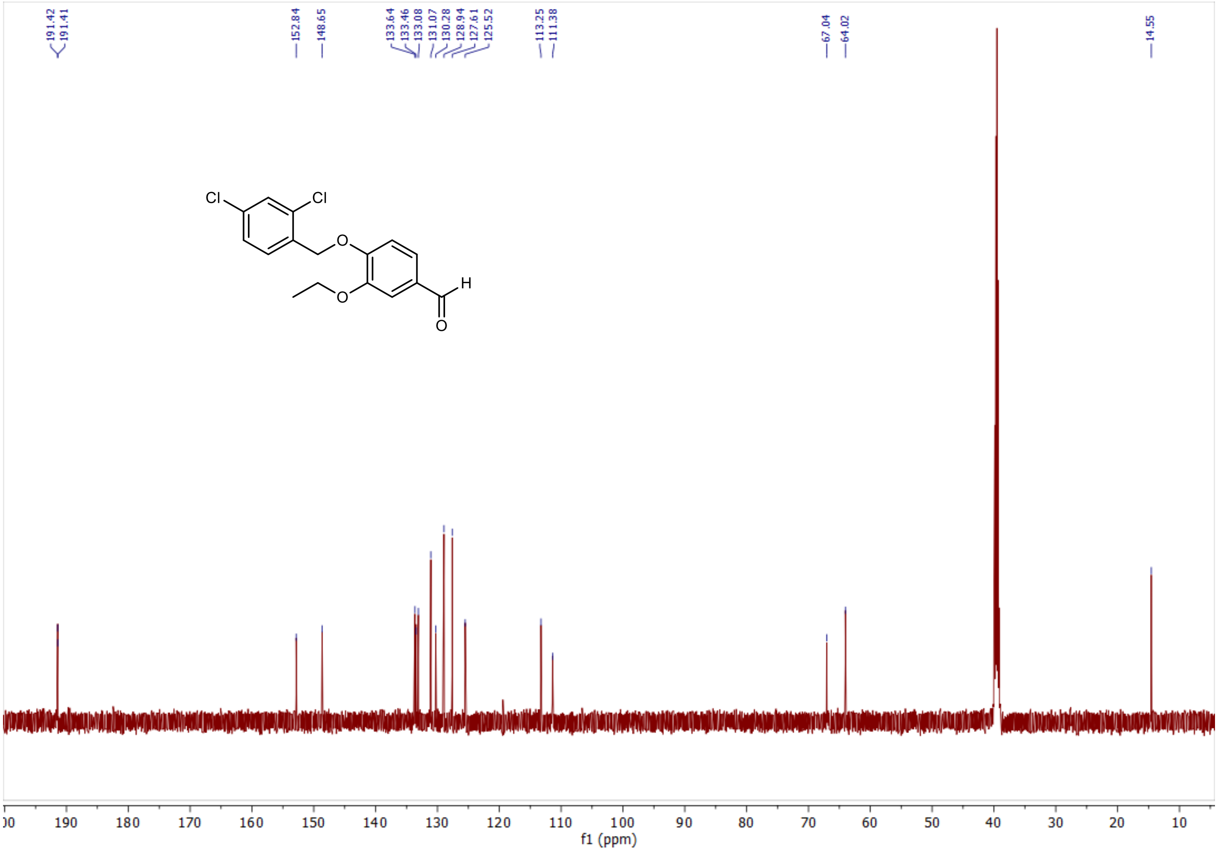

Supplementary Figure 24. ^{1}H-NMR (500 MHz, DMSO-d$_6$) and ^{13}C-NMR (126 MHz, DMSO-d_6) spectrum of **PB-2.2**.

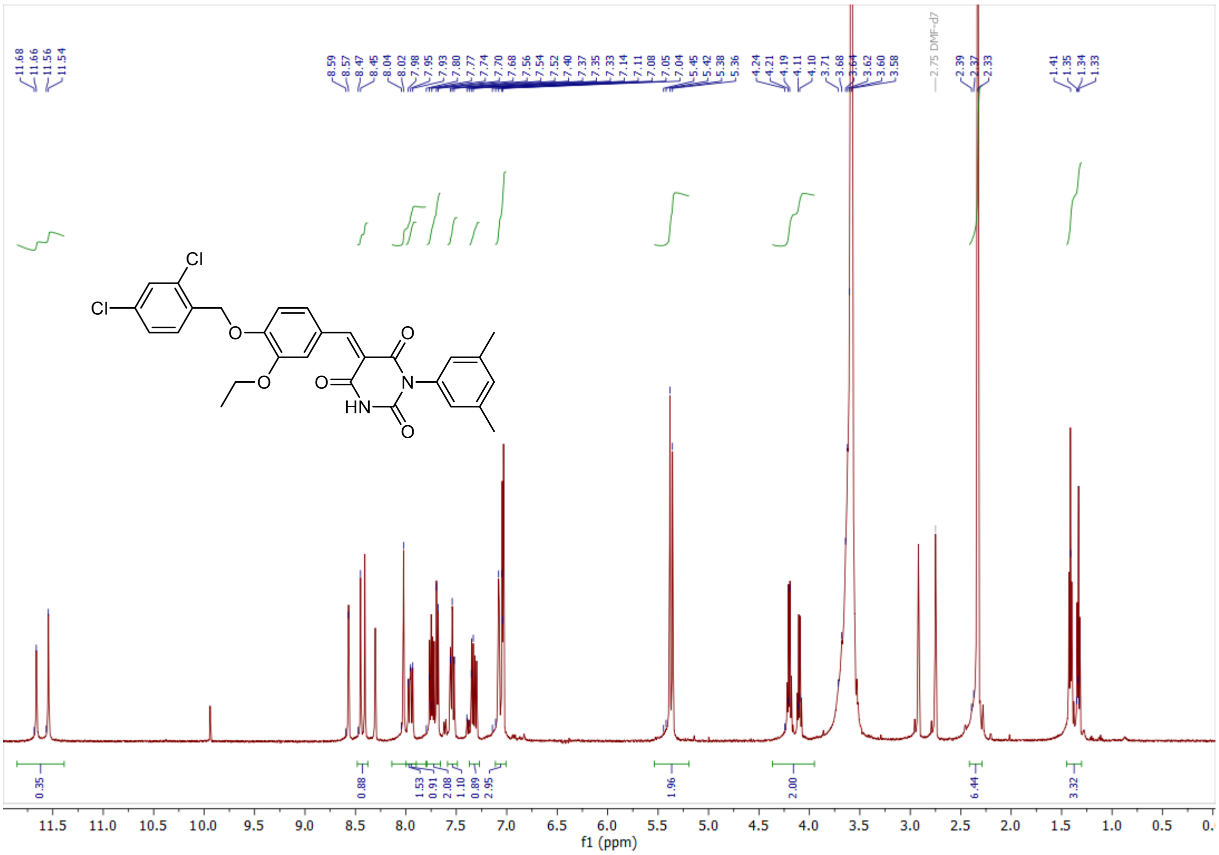

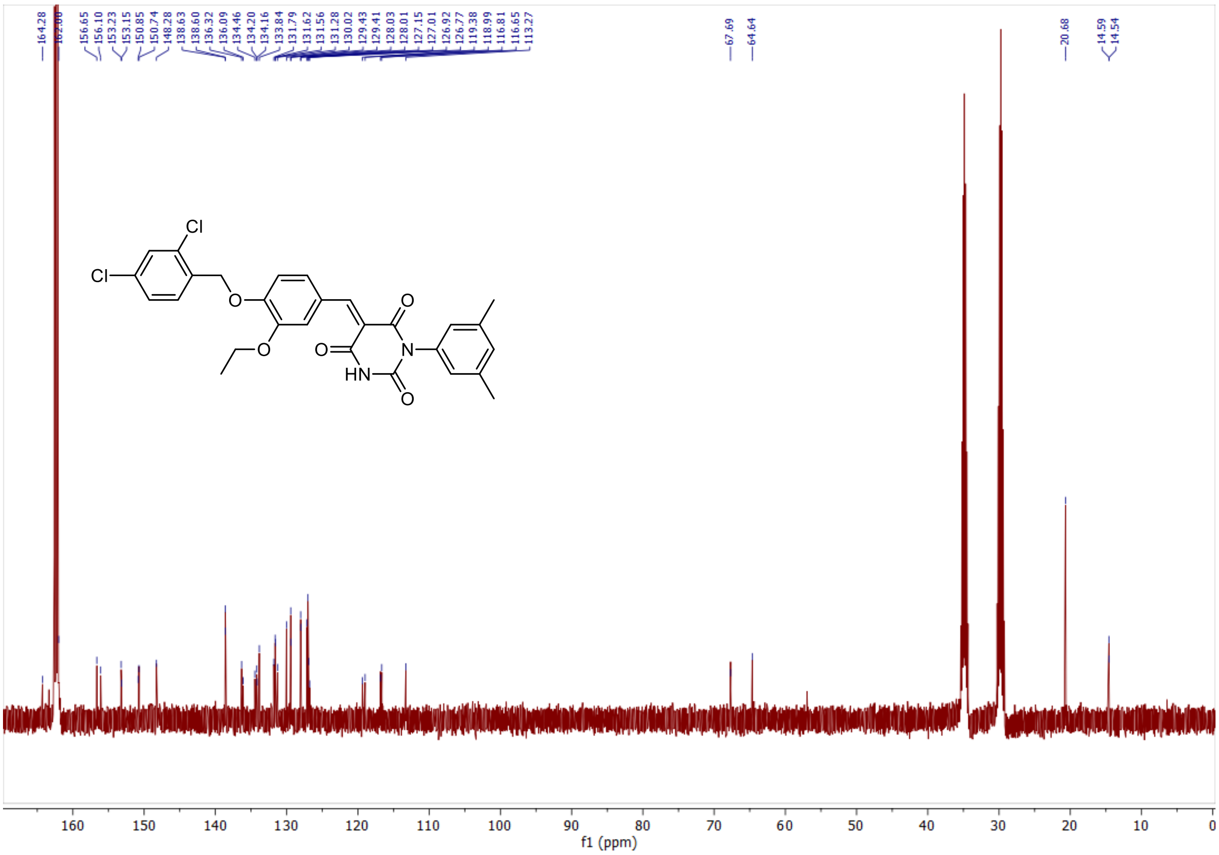

Supplementary Figure 25. ^{1}H-NMR (500 MHz, DMF-d_7) and ^{13}C-NMR (126 MHz, DMF-d_7) spectrum of **PB-2**.

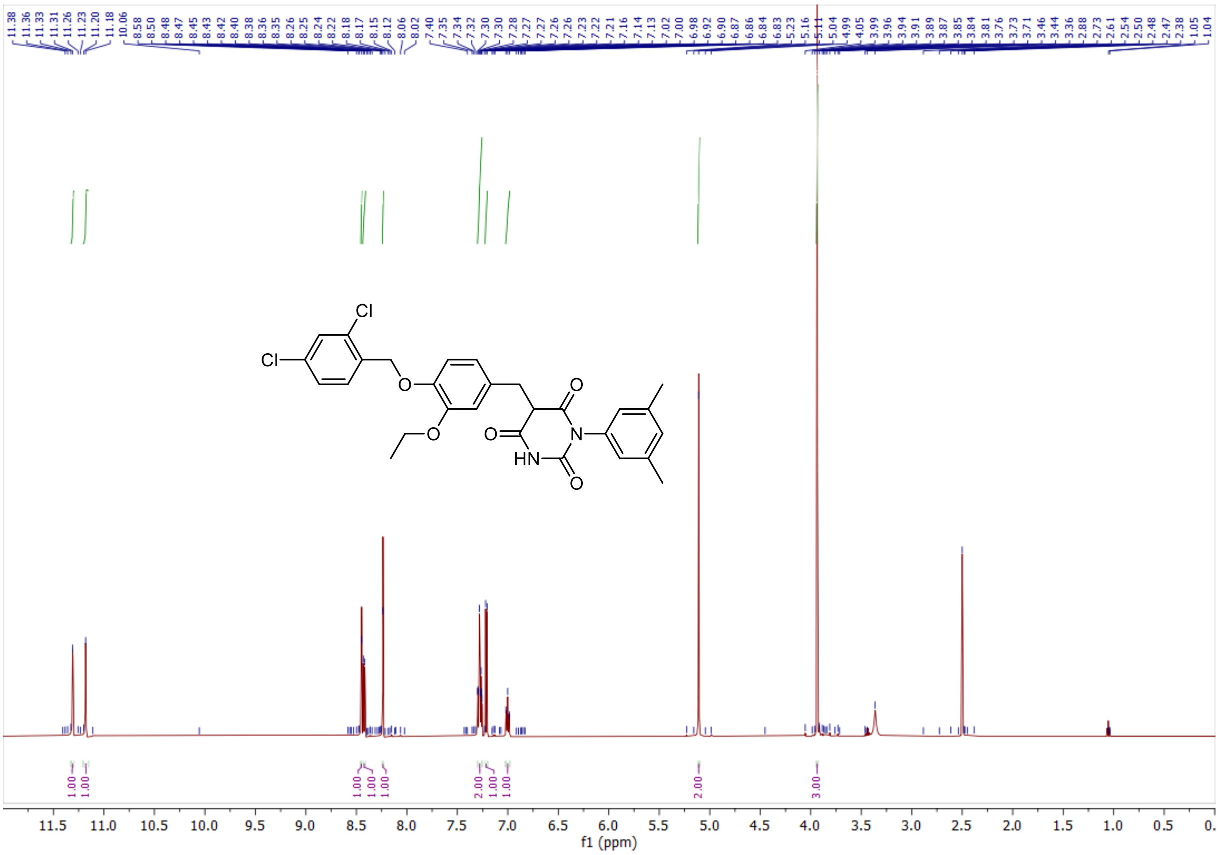

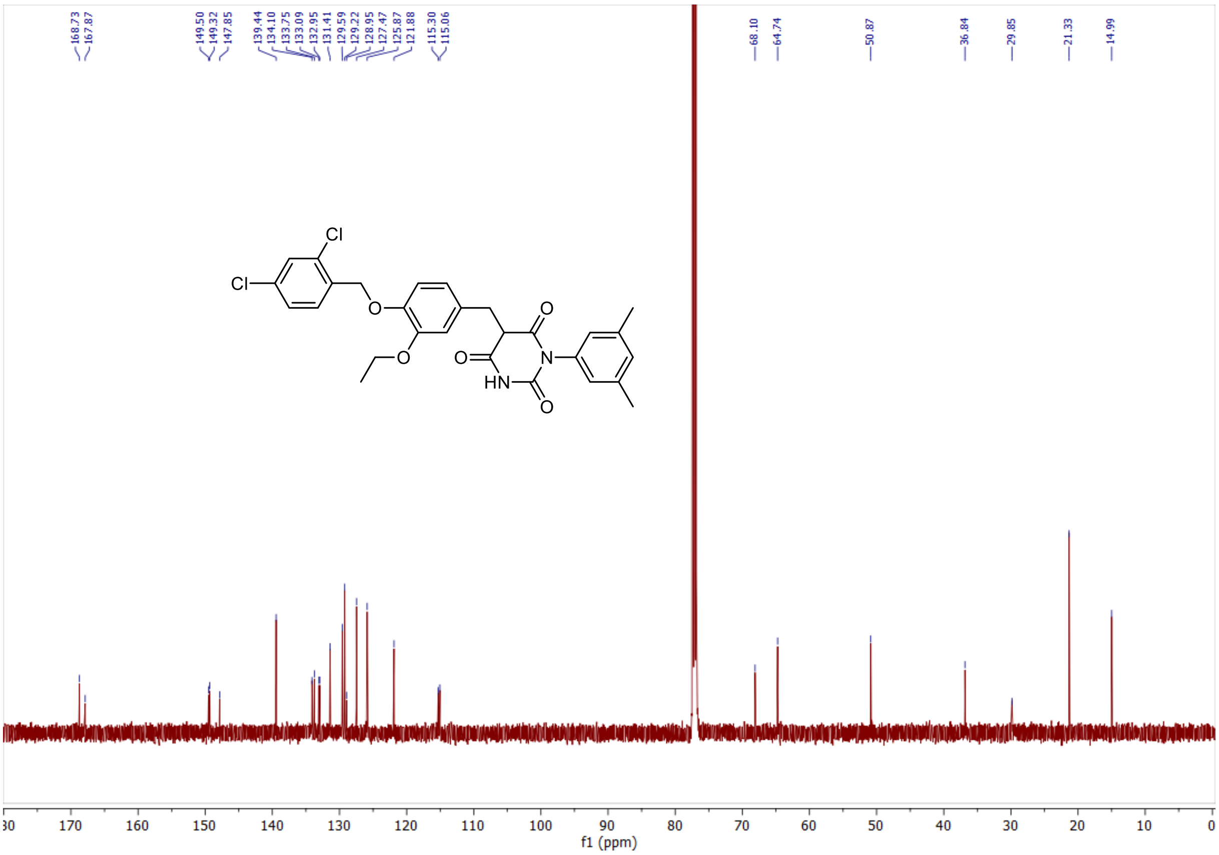

Supplementary Figure 26. ^{1}H-NMR (500 MHz, Chloroform-d) and ^{13}C-NMR (126 MHz, Chloroform-*d*) spectrum of **PB-2.1**.

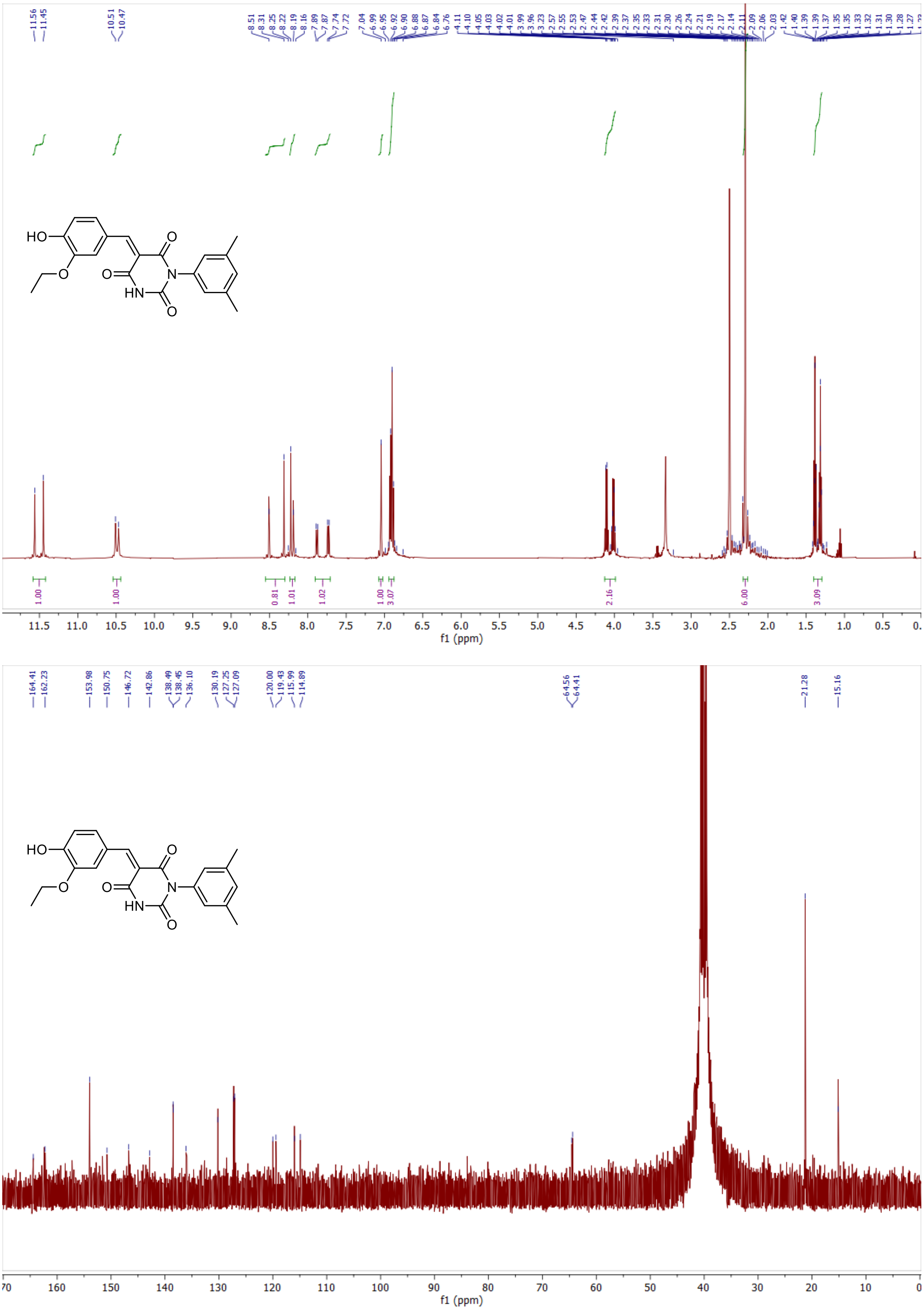

Supplementary Figure 27. ^{1}H-NMR (500 MHz, DMSO-d$_6$) and ^{13}C-NMR (126 MHz, DMSO-d_6) spectrum of **PB-2.4**.

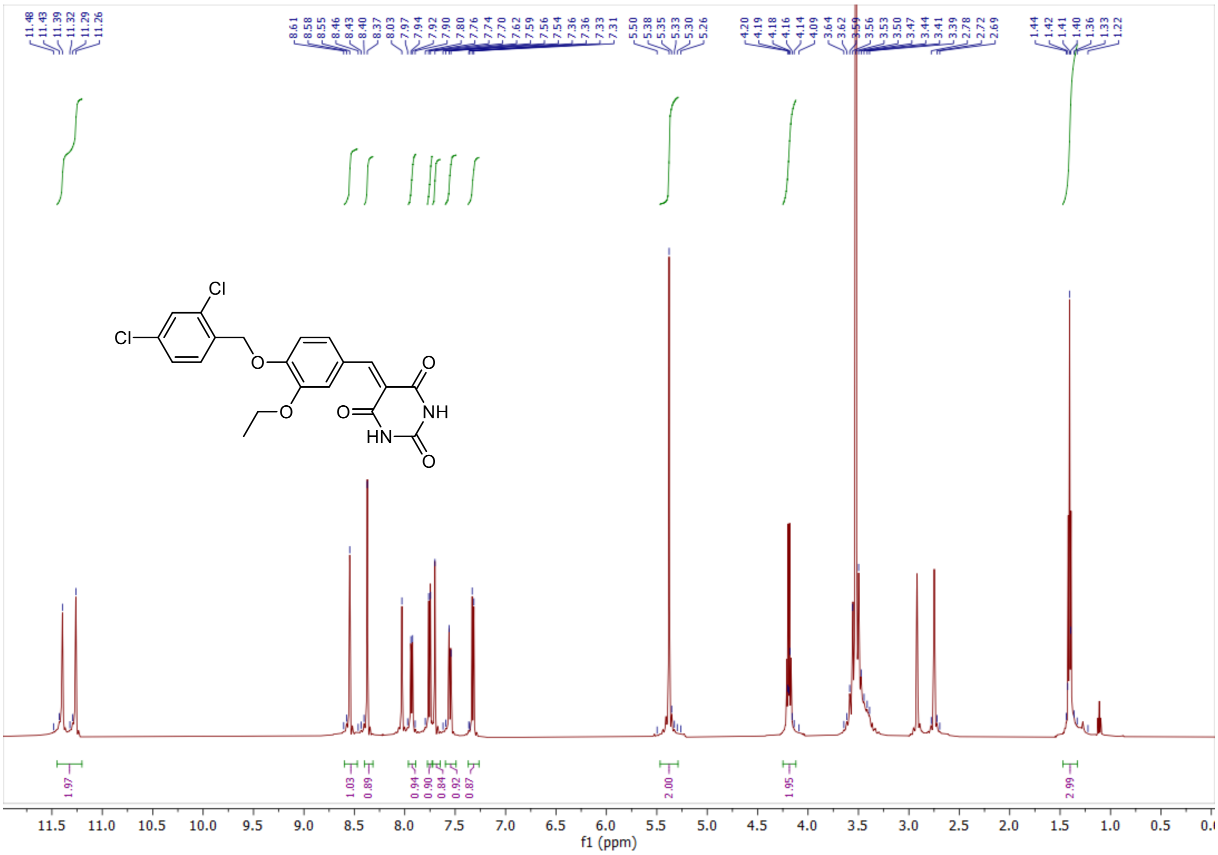

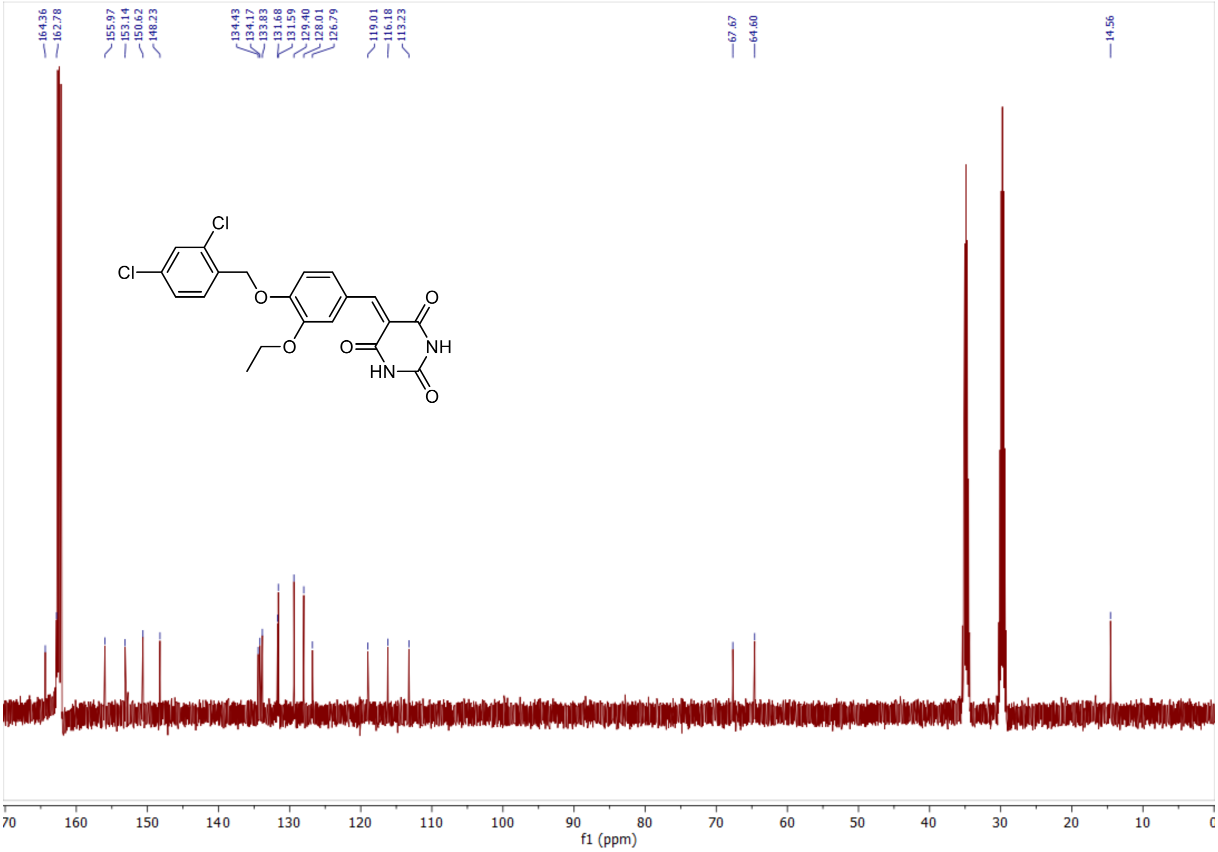

Supplementary Figure 28. ^{1}H-NMR (500 MHz, DMF-d$_7$) and ^{13}C-NMR (126 MHz, DMF-*d*$_7$) spectrum of **PB-2.5**.

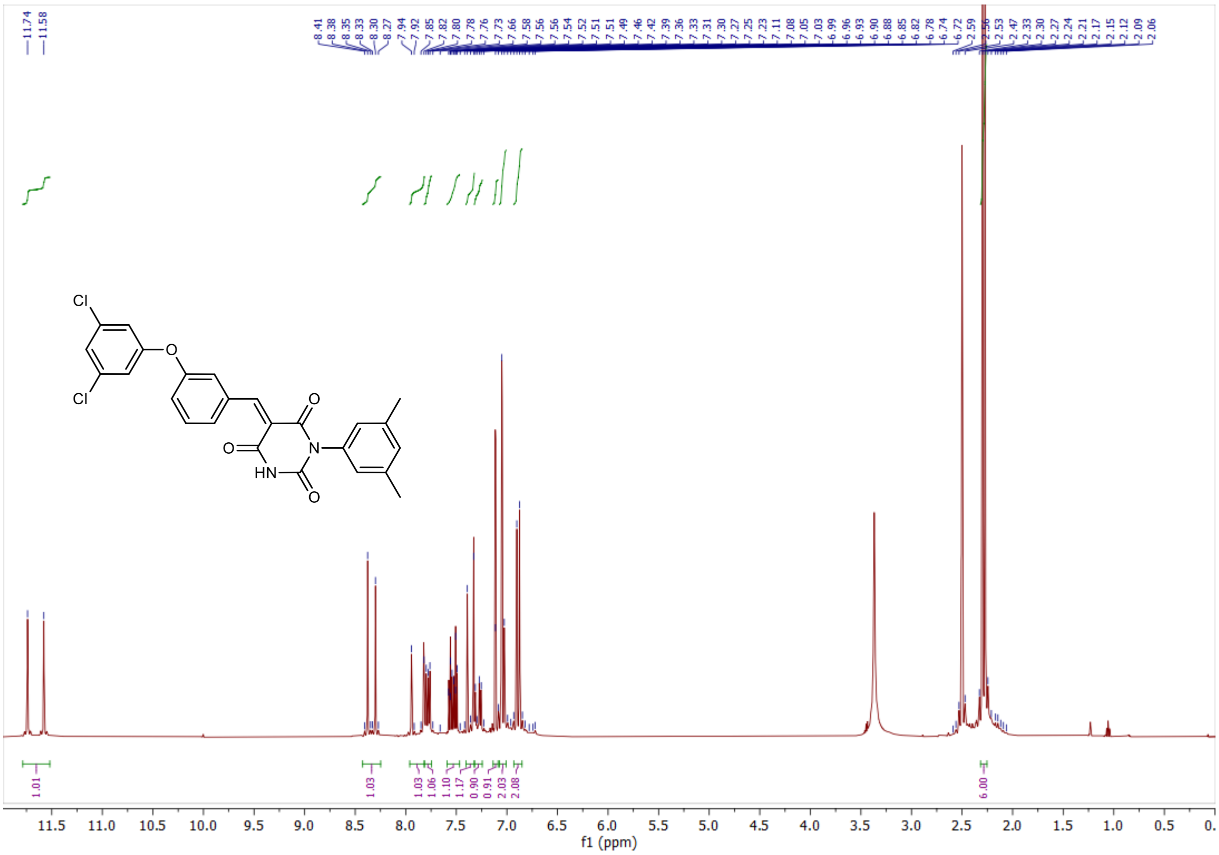

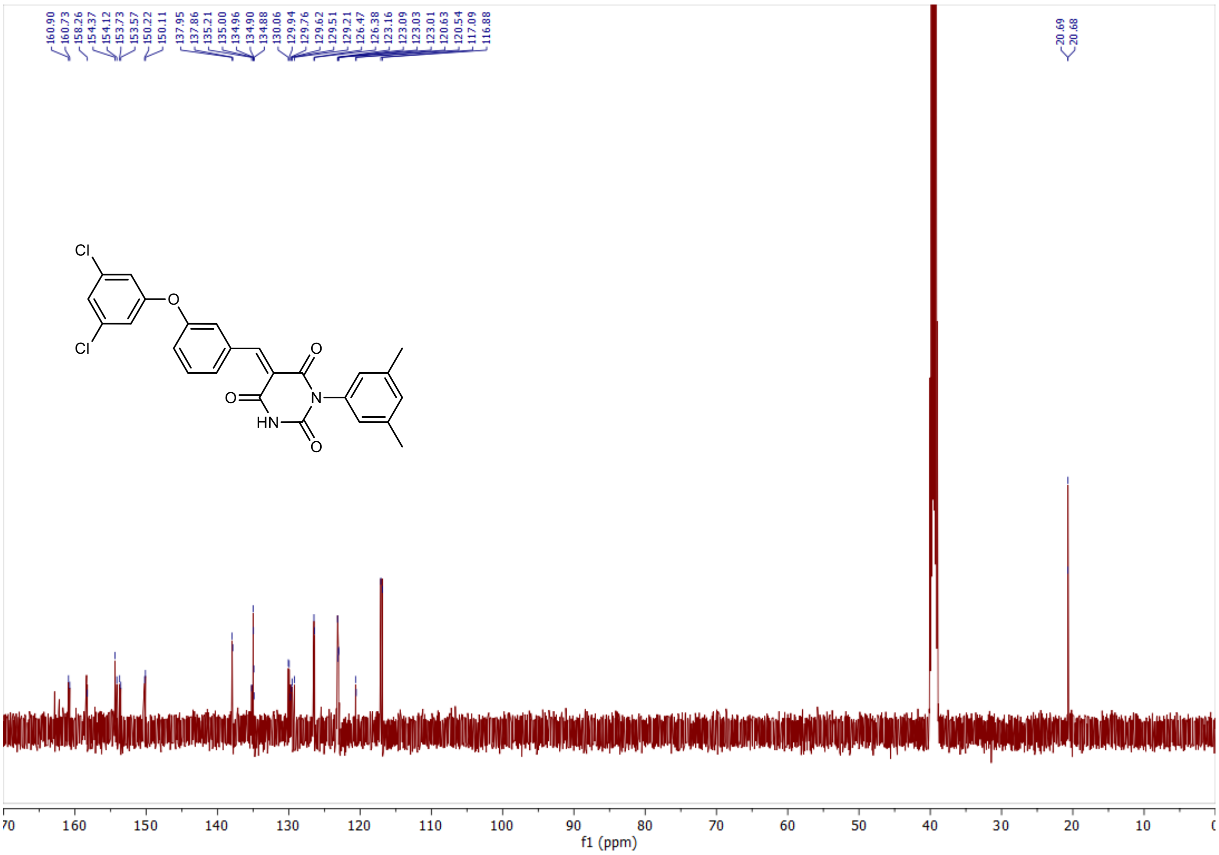

Supplementary Figure 29. ^{1}H-NMR (500 MHz, DMSO-d$_6$) and ^{13}C-NMR (126 MHz, DMSO-d_6) spectrum of **PB-2.6**.

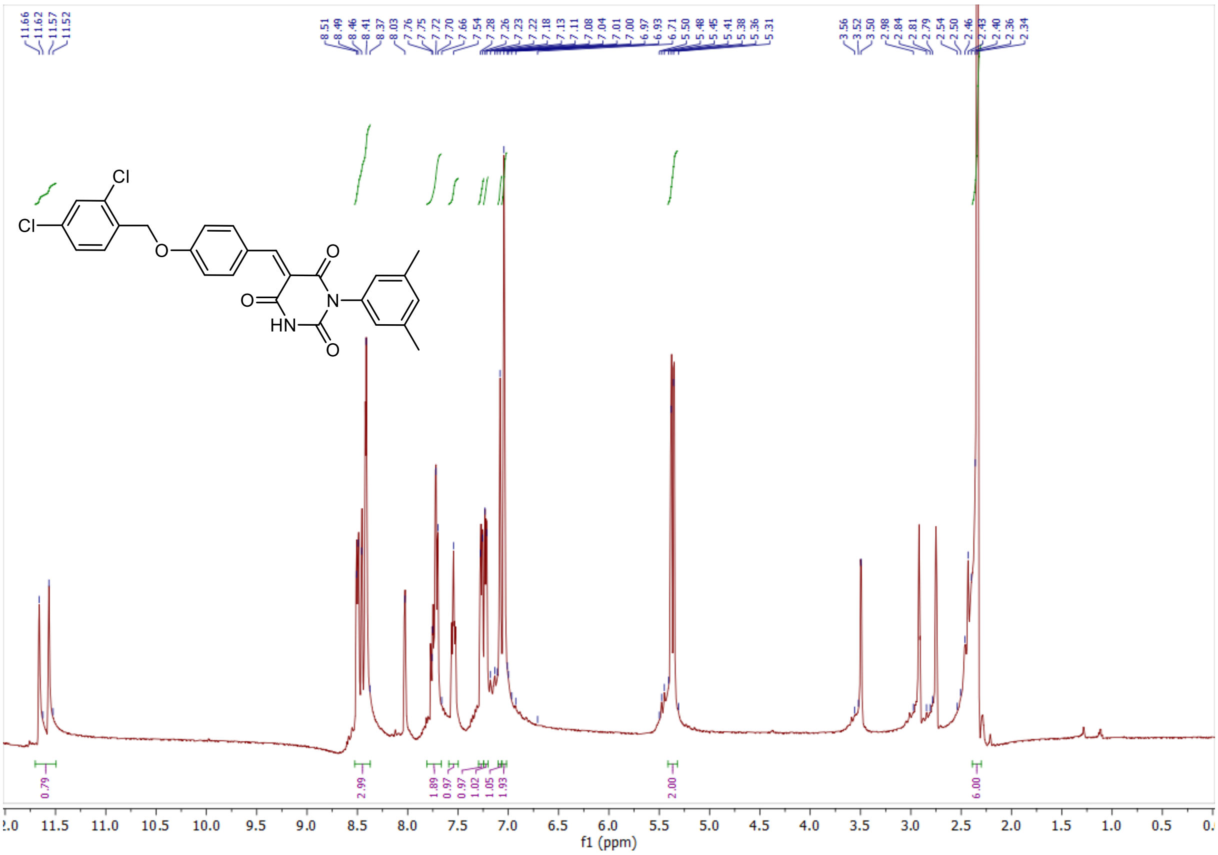

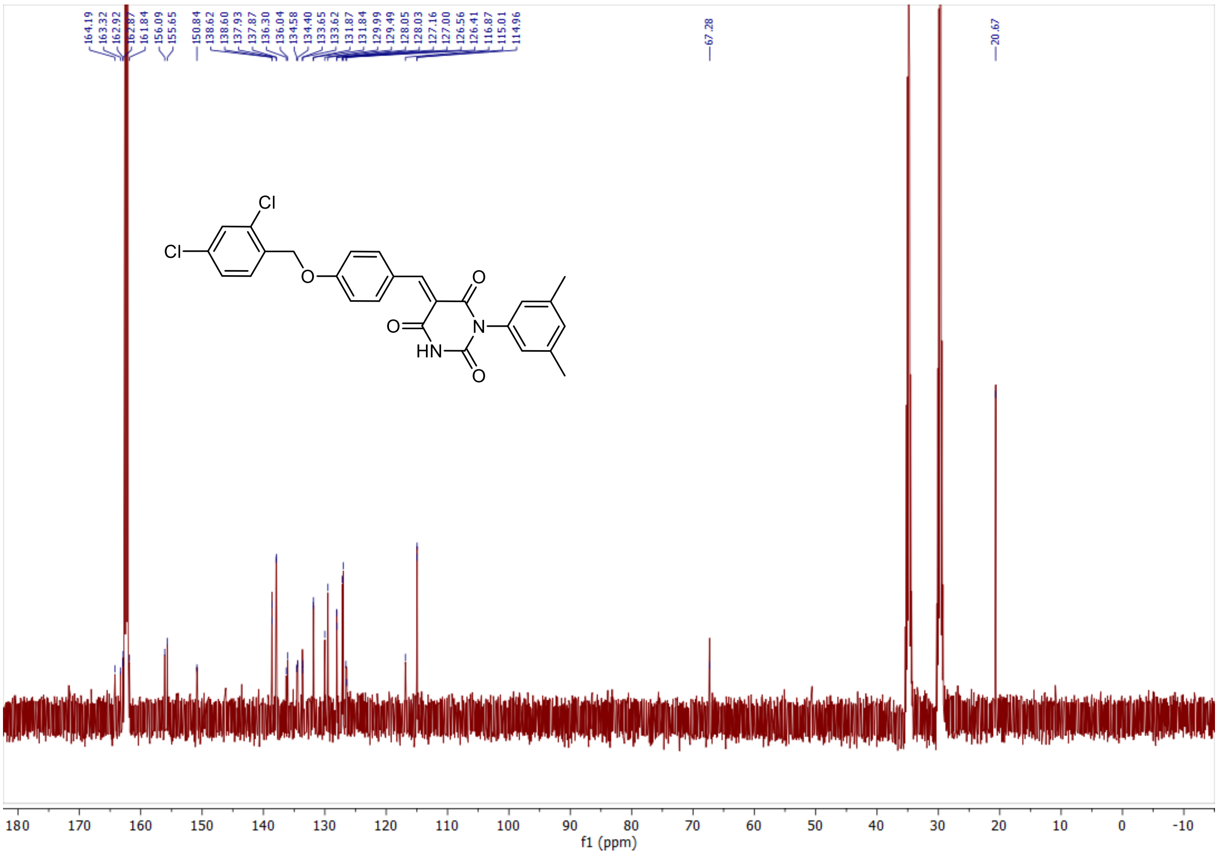

Supplementary Figure 30. ^{1}H-NMR (500 MHz, DMF-d_7) and ^{13}C-NMR (126 MHz, DMF-d_7) spectrum of **PB-2.8**.

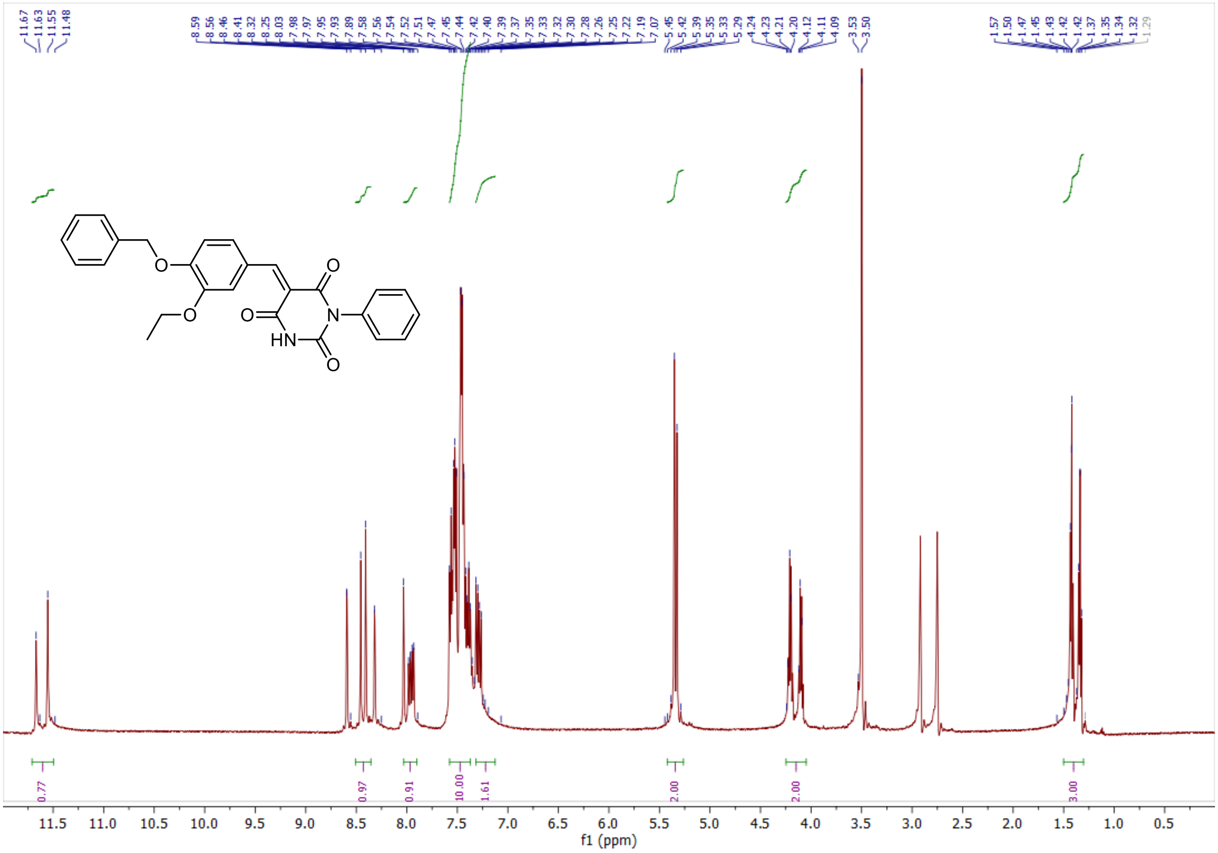

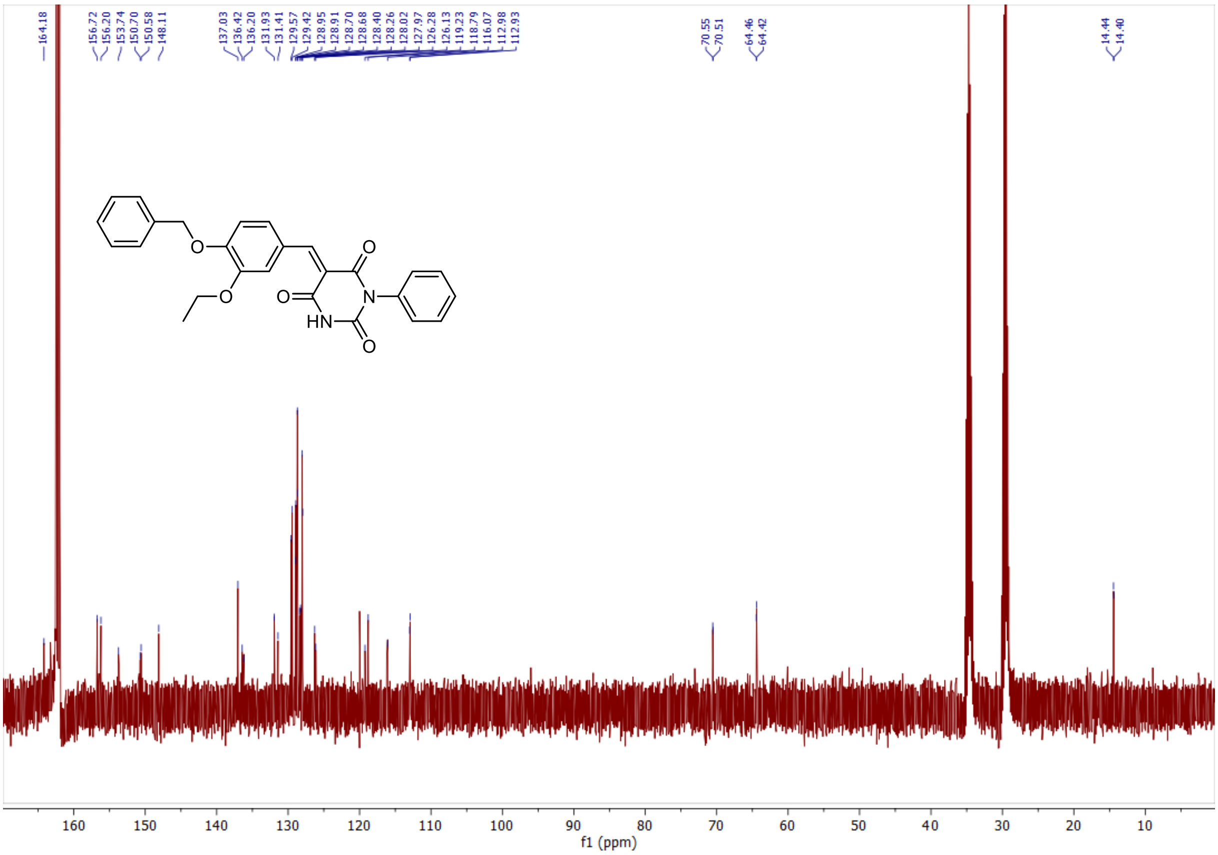

Supplementary Figure 31. ^{1}H-NMR (500 MHz, DMF-d$_7$) and ^{13}C-NMR (126 MHz, DMF-*d*$_7$) spectrum of **PB-2.10**.

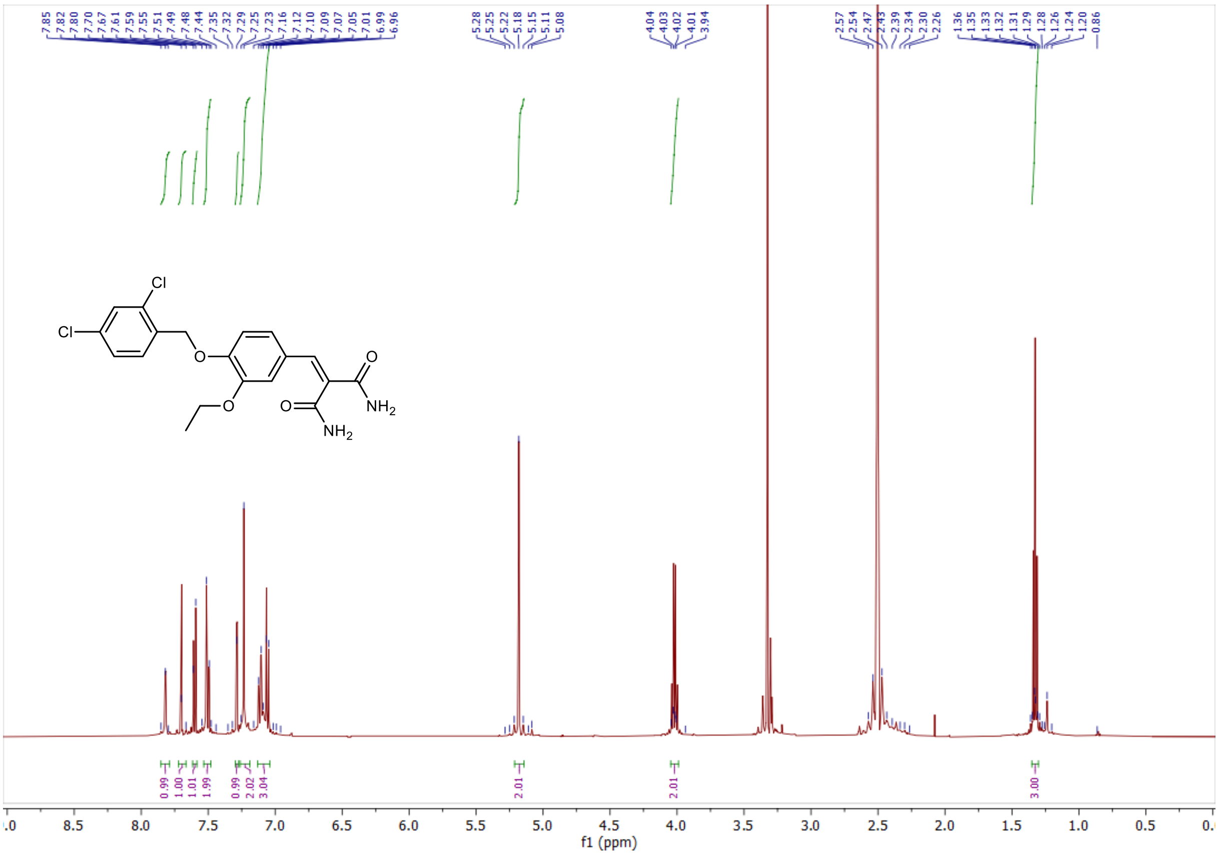

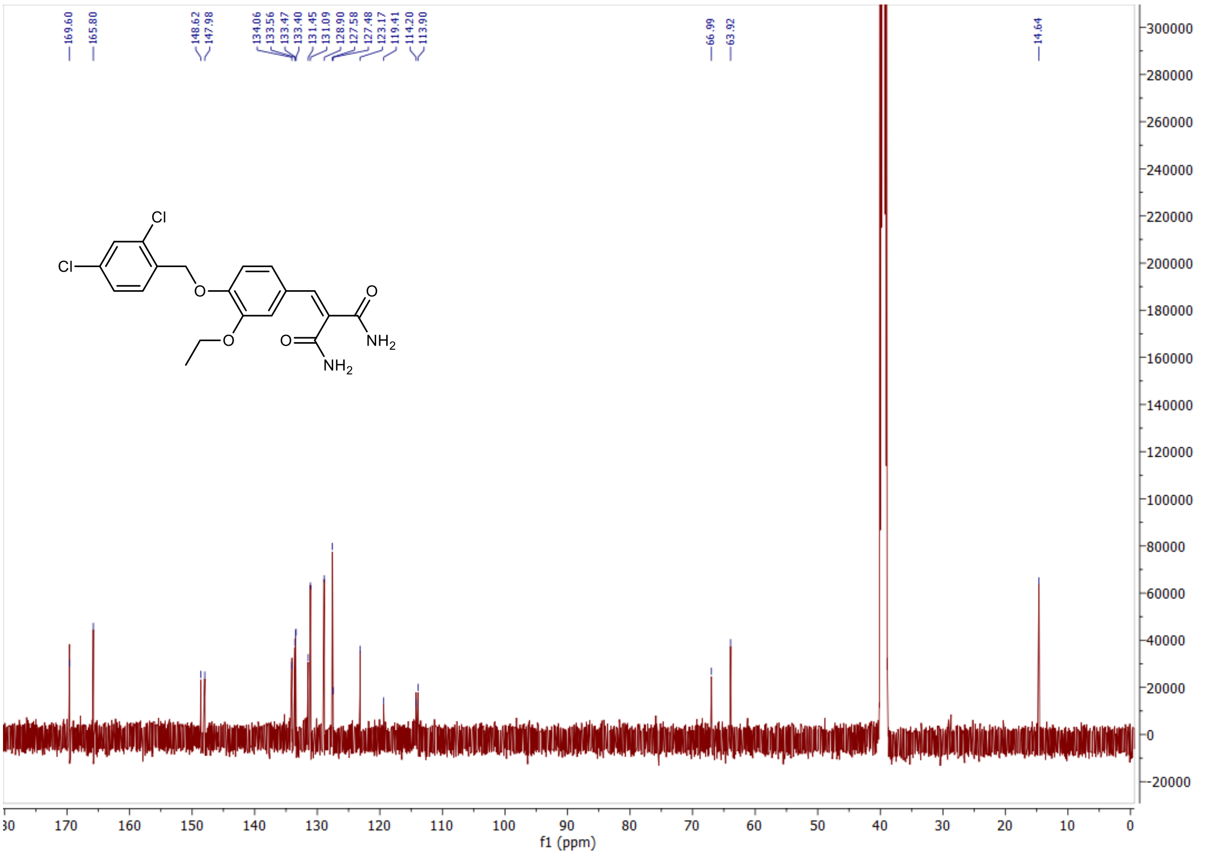

Supplementary Figure 32. ^{1}H-NMR (500 MHz, DMSO-d$_6$) and ^{13}C-NMR (126 MHz, DMSO-d_6) spectrum of **PB-2.12**.

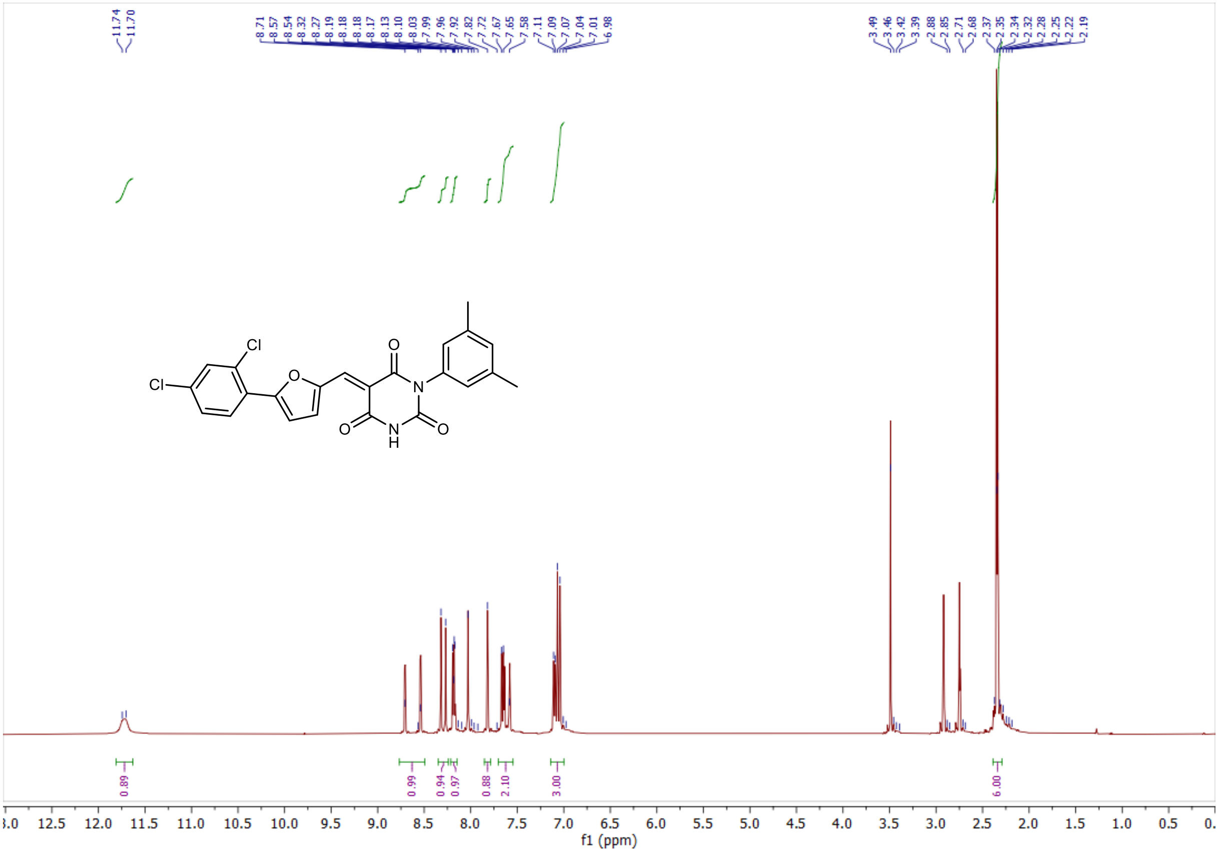

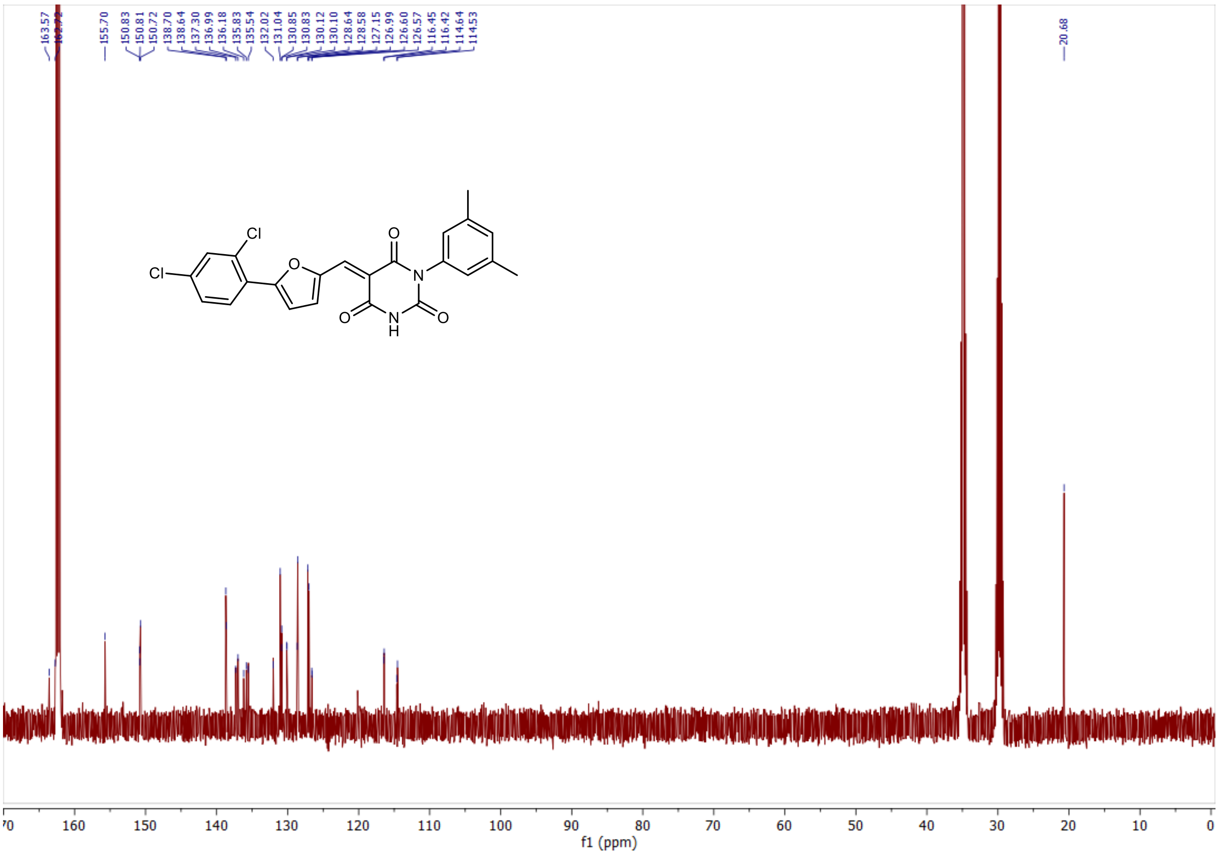

Supplementary Figure 33. ^{1}H-NMR (500 MHz, DMF-d$_7$) and ^{13}C-NMR (126 MHz, DMF-d_7) spectrum of **PB-3**.

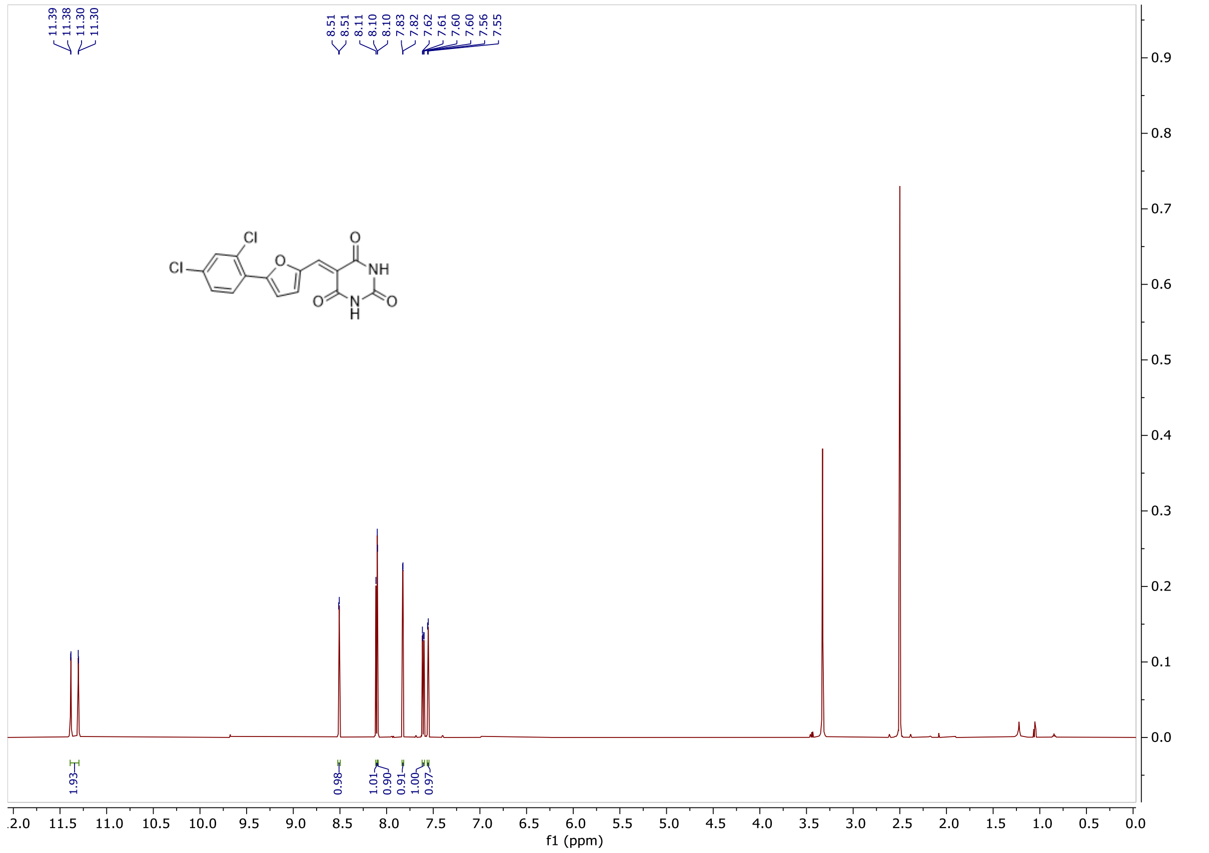

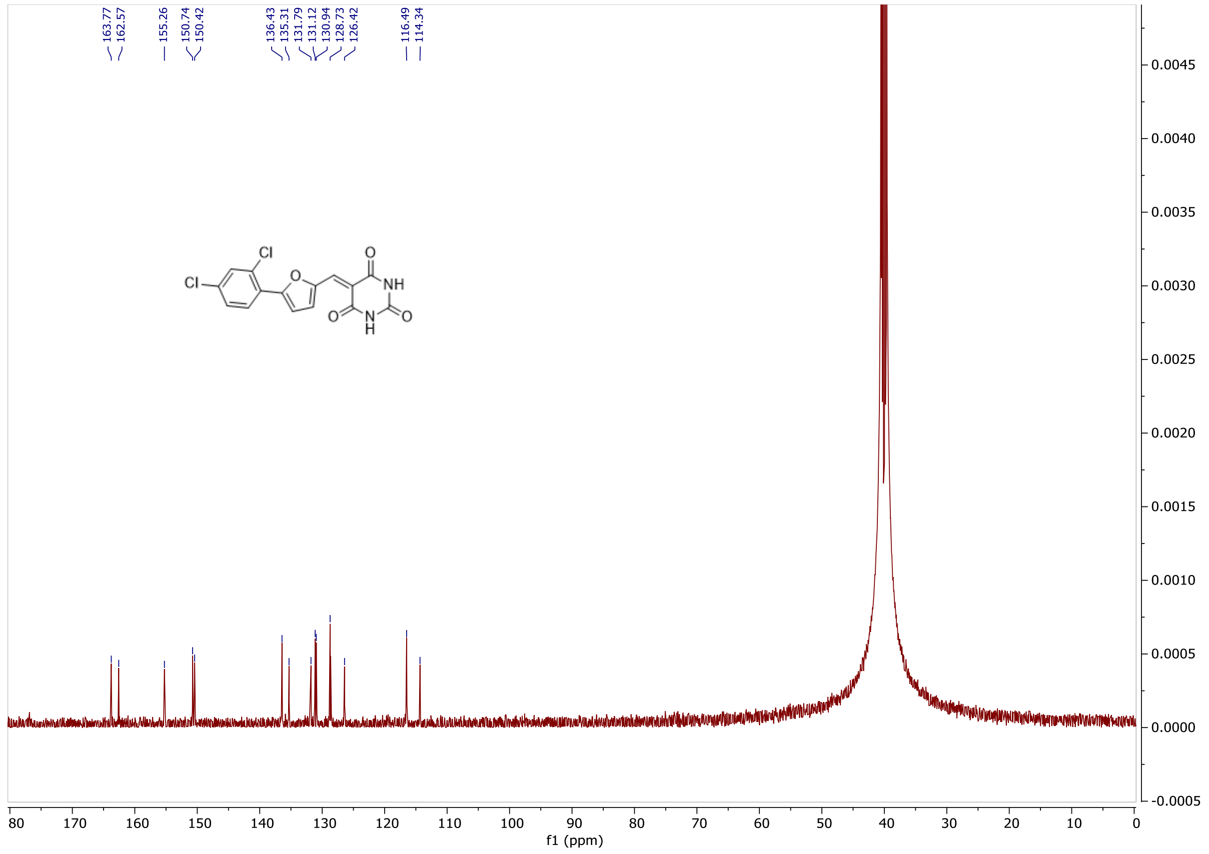

Supplementary Figure 34. ^{1}H-NMR (600 MHz, DMSO-d$_6$) and ^{13}C-NMR (151 MHz, DMSO-*d*$_6$) spectrum of **PB-3.2**.

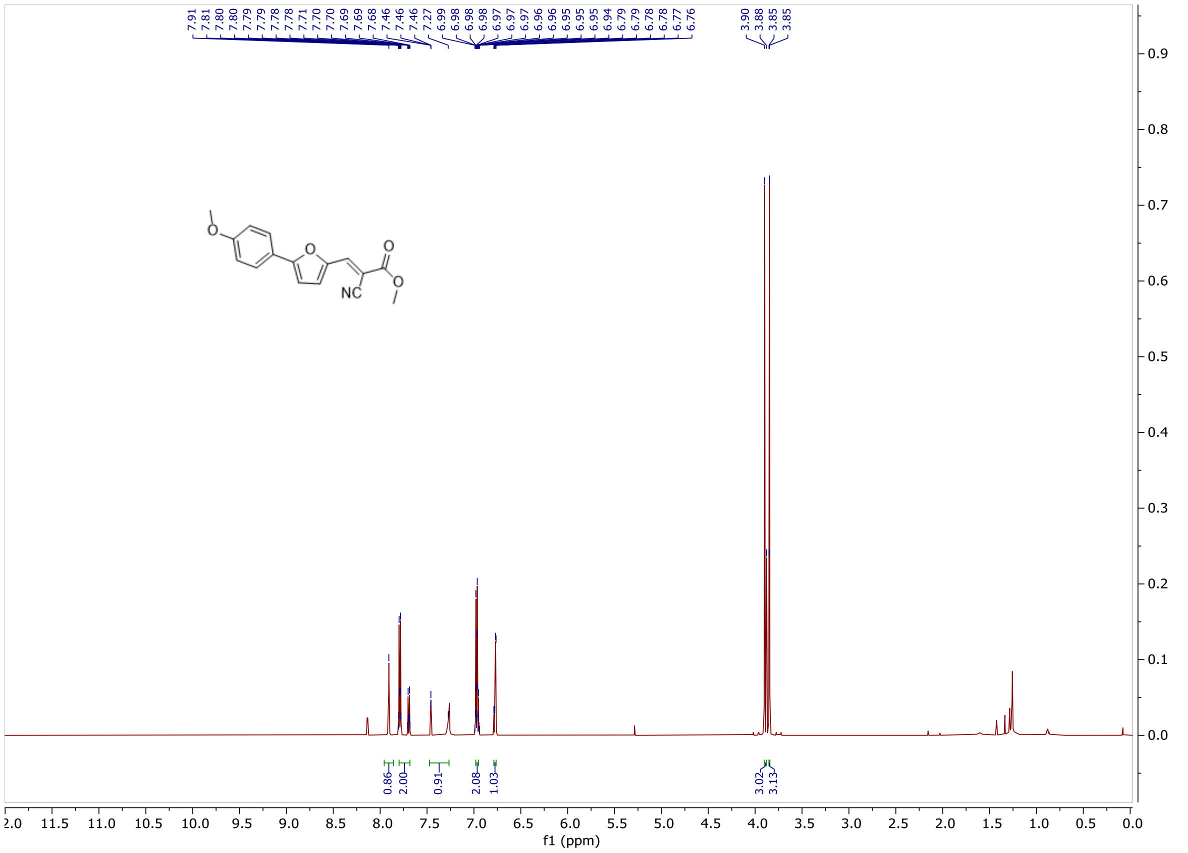

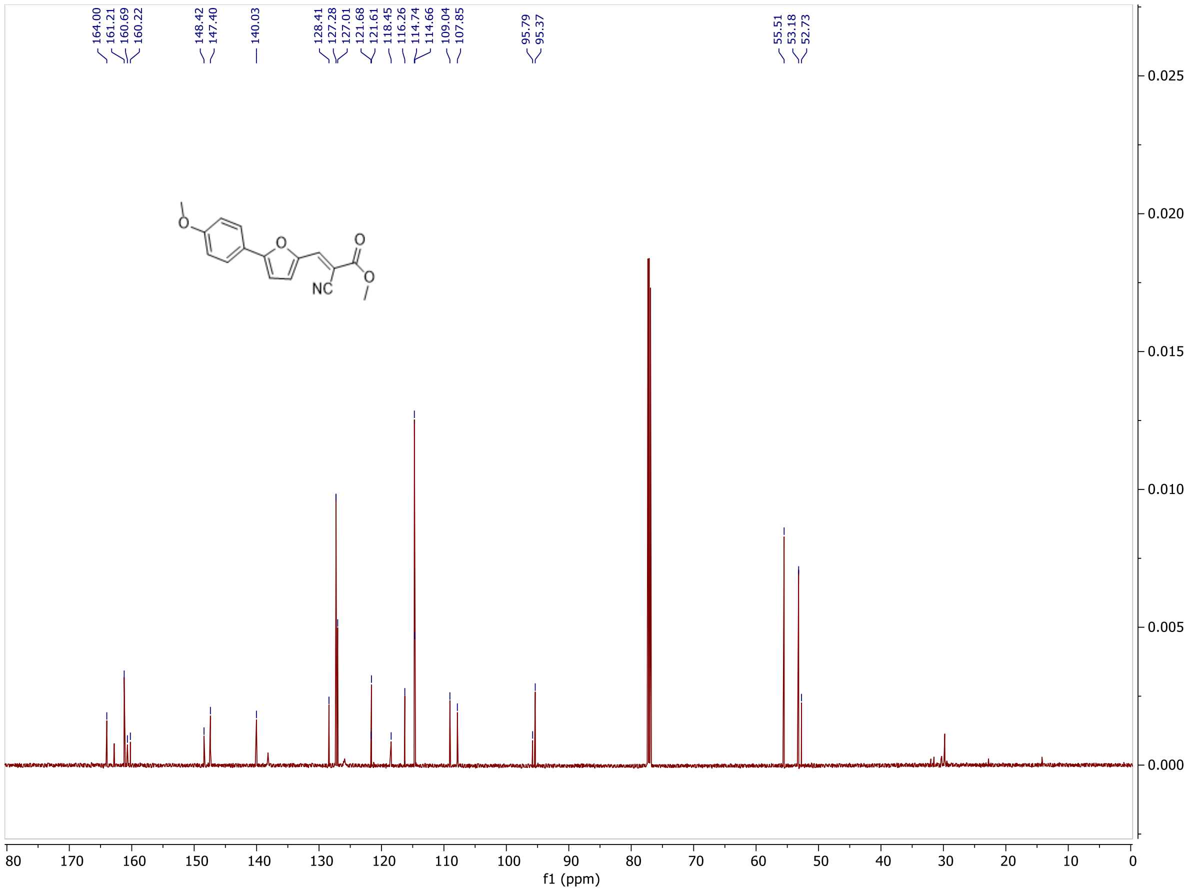

Supplementary Figure 35. ^{1}H-NMR (500 MHz, $CDCl_3$) and ^{13}C-NMR (151 MHz, $CDCl_3$) spectrum of **PB-3.3.**

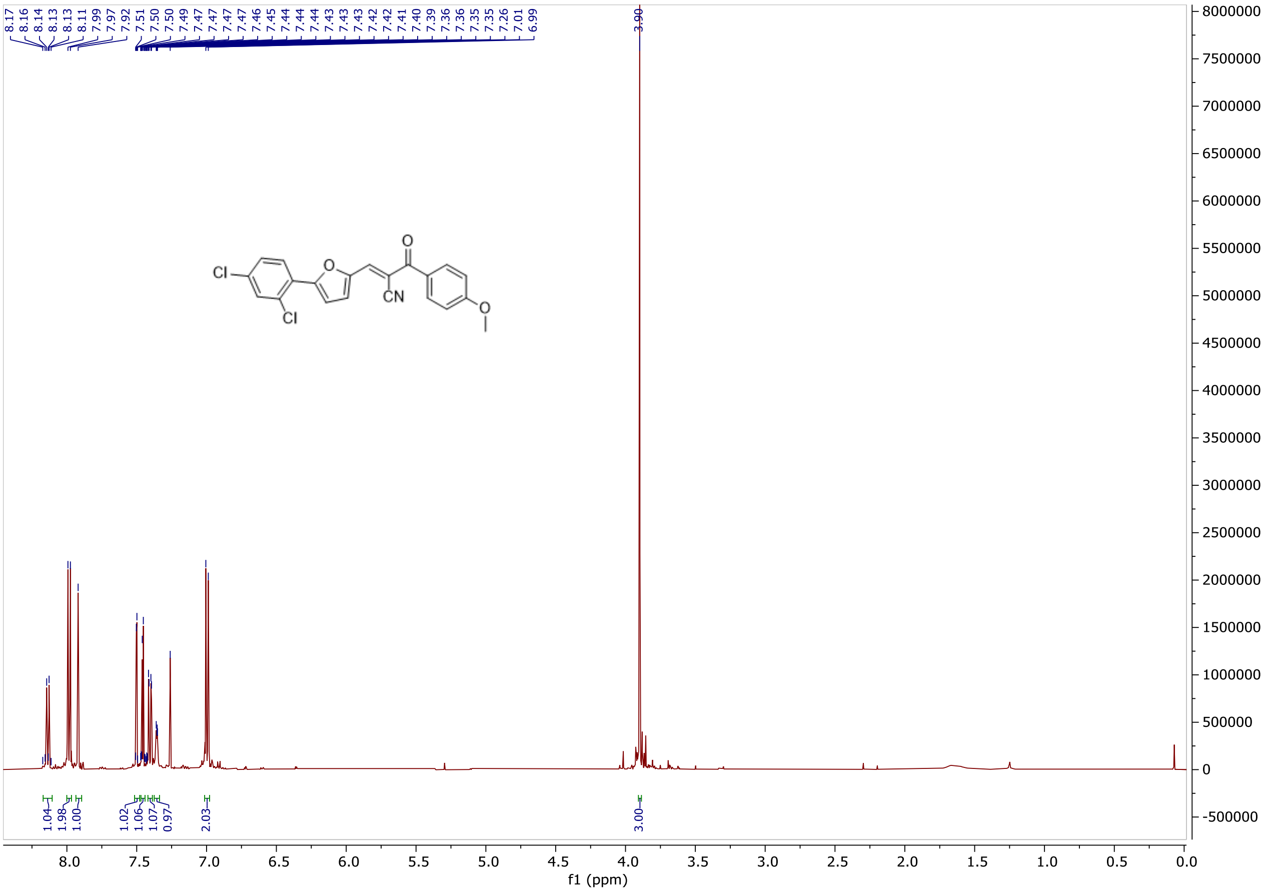

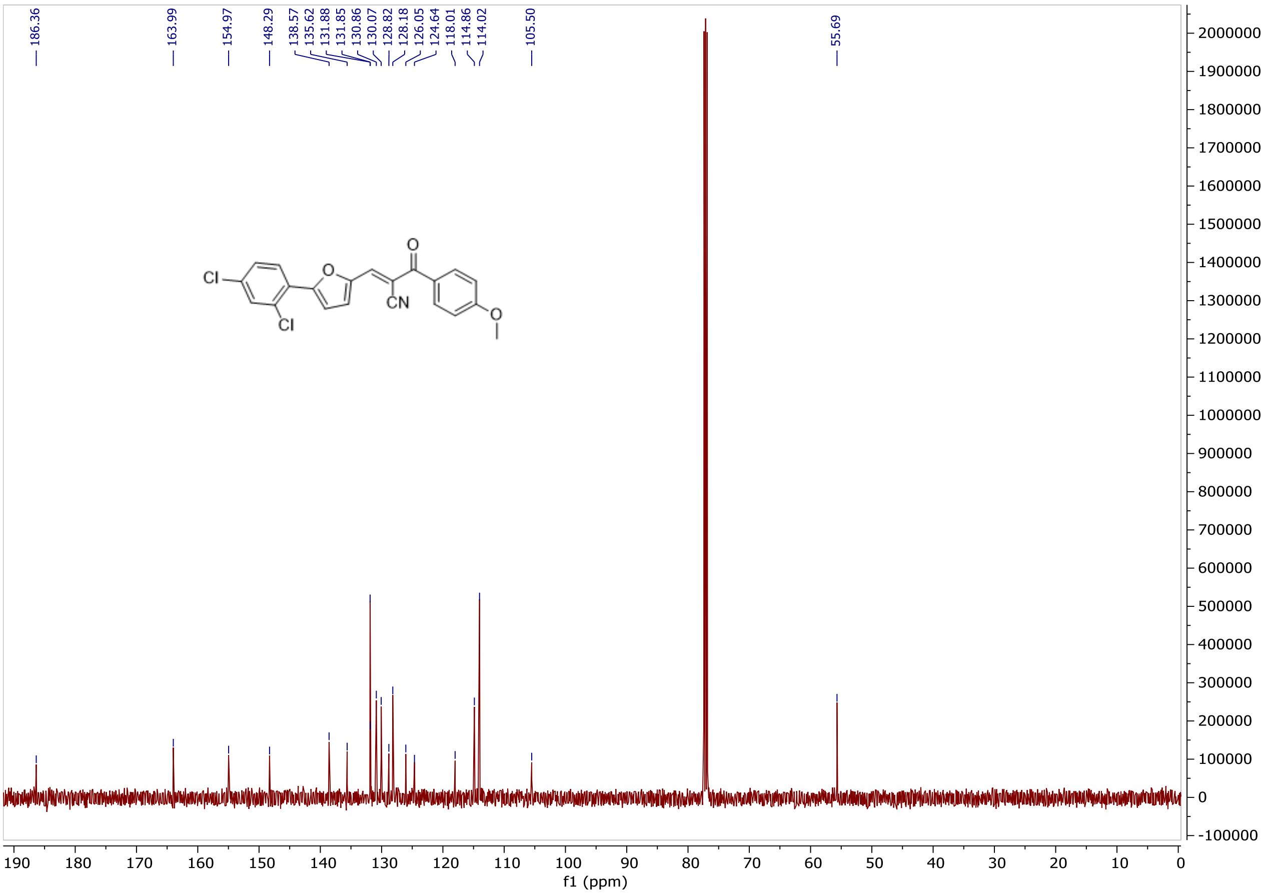

Supplementary Figure 36. ^{1}H-NMR (500 MHz, $CDCl_3$) and ^{13}C-NMR (126 MHz, $CDCl_3$) spectrum of **PB-3.4**.

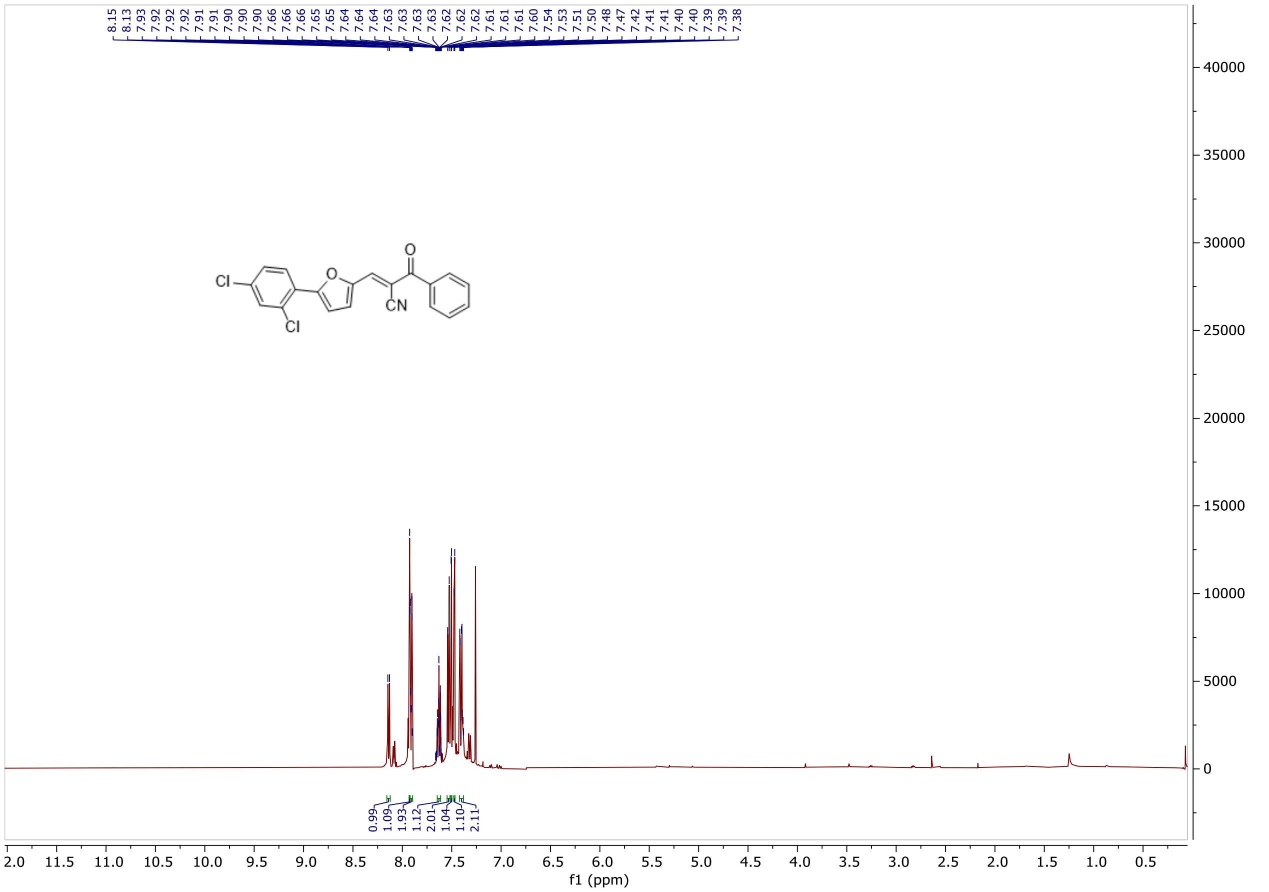

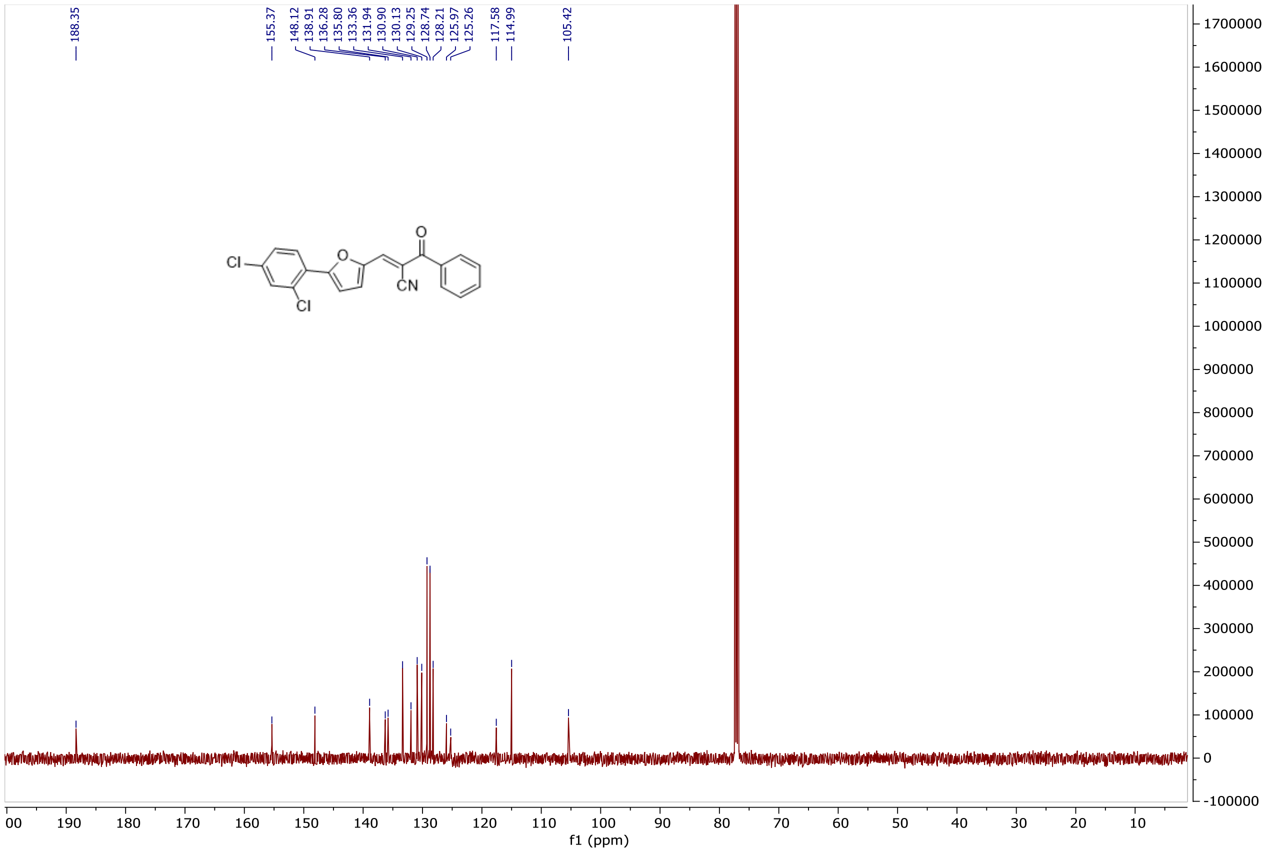

Supplementary Figure 37. ^{1}H-NMR (500 MHz, $CDCl_3$) and ^{13}C-NMR (126 MHz, $CDCl_3$) spectrum of **PB-3.5**.

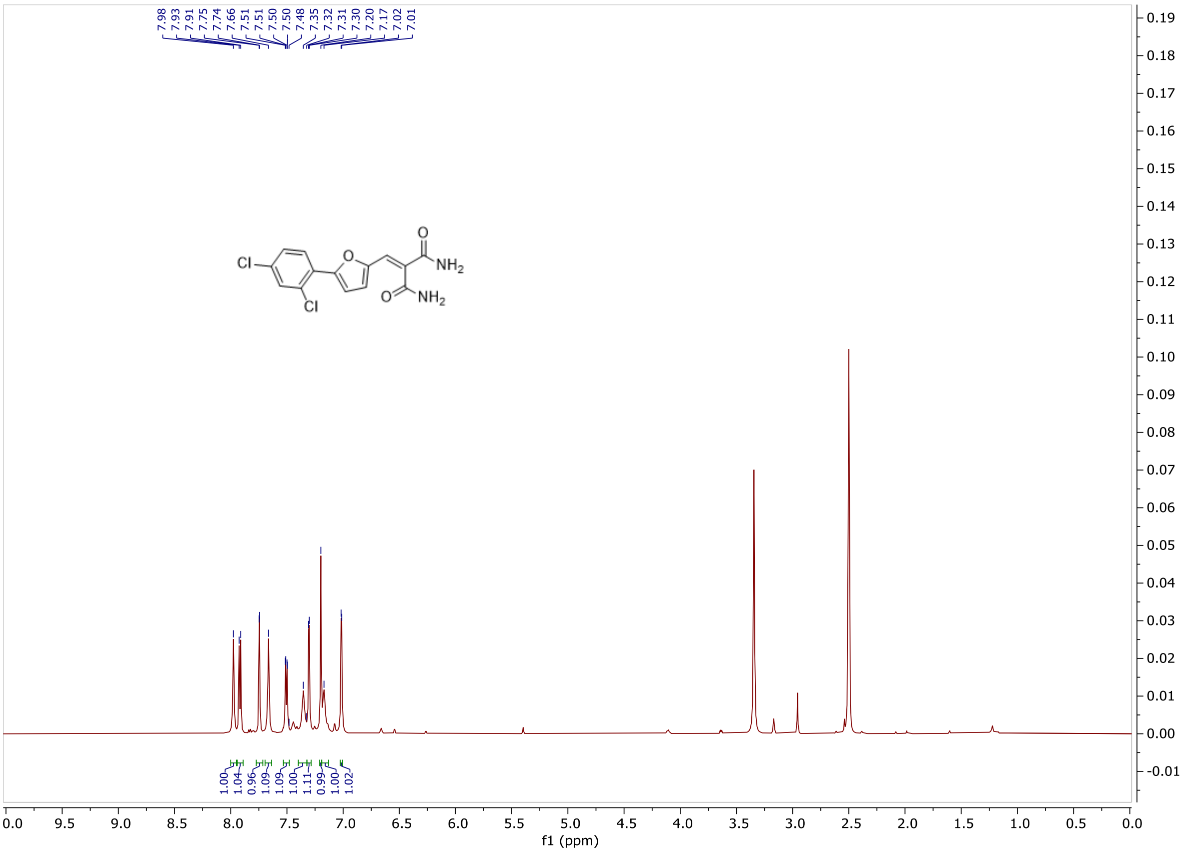

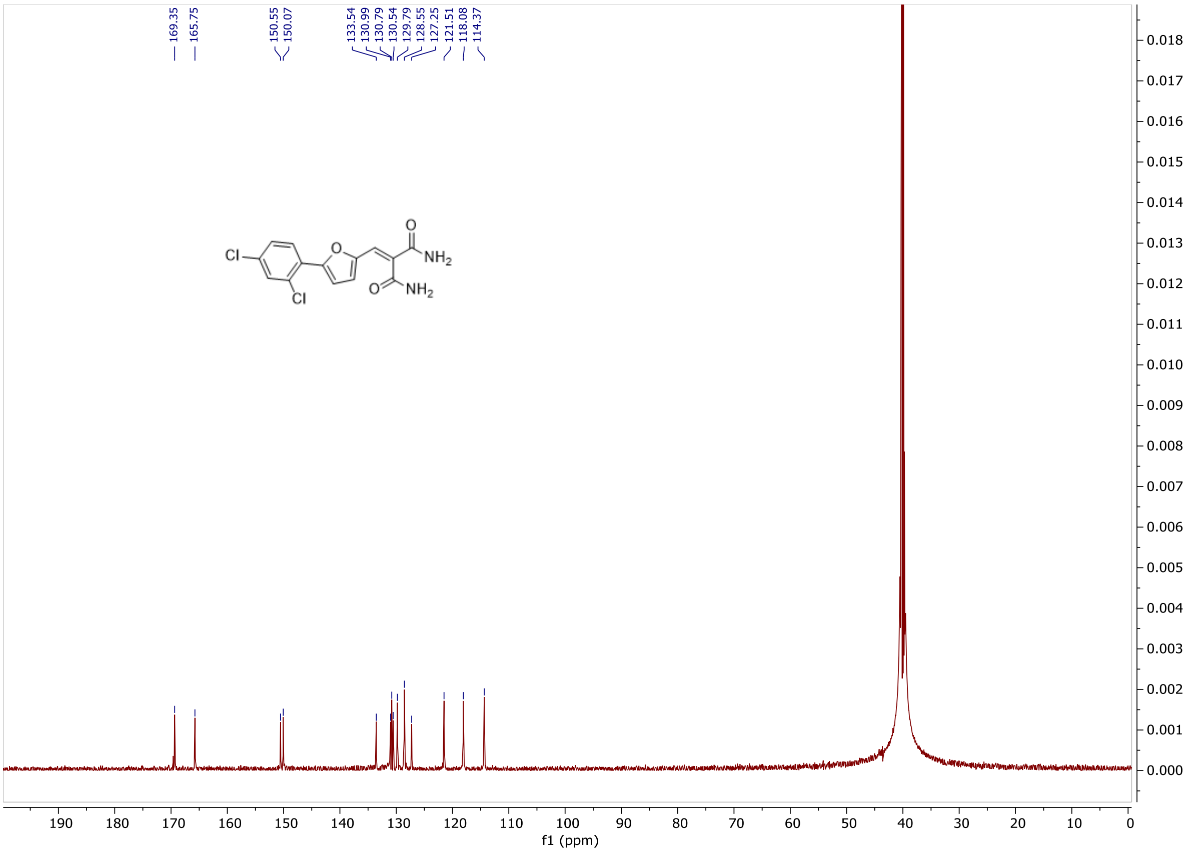

Supplementary Figure 38. ^{1}H-NMR (600 MHz, DMSO-d_6) and ^{13}C-NMR (151 MHz, DMSO-d_6) spectrum of **PB-3.6**.

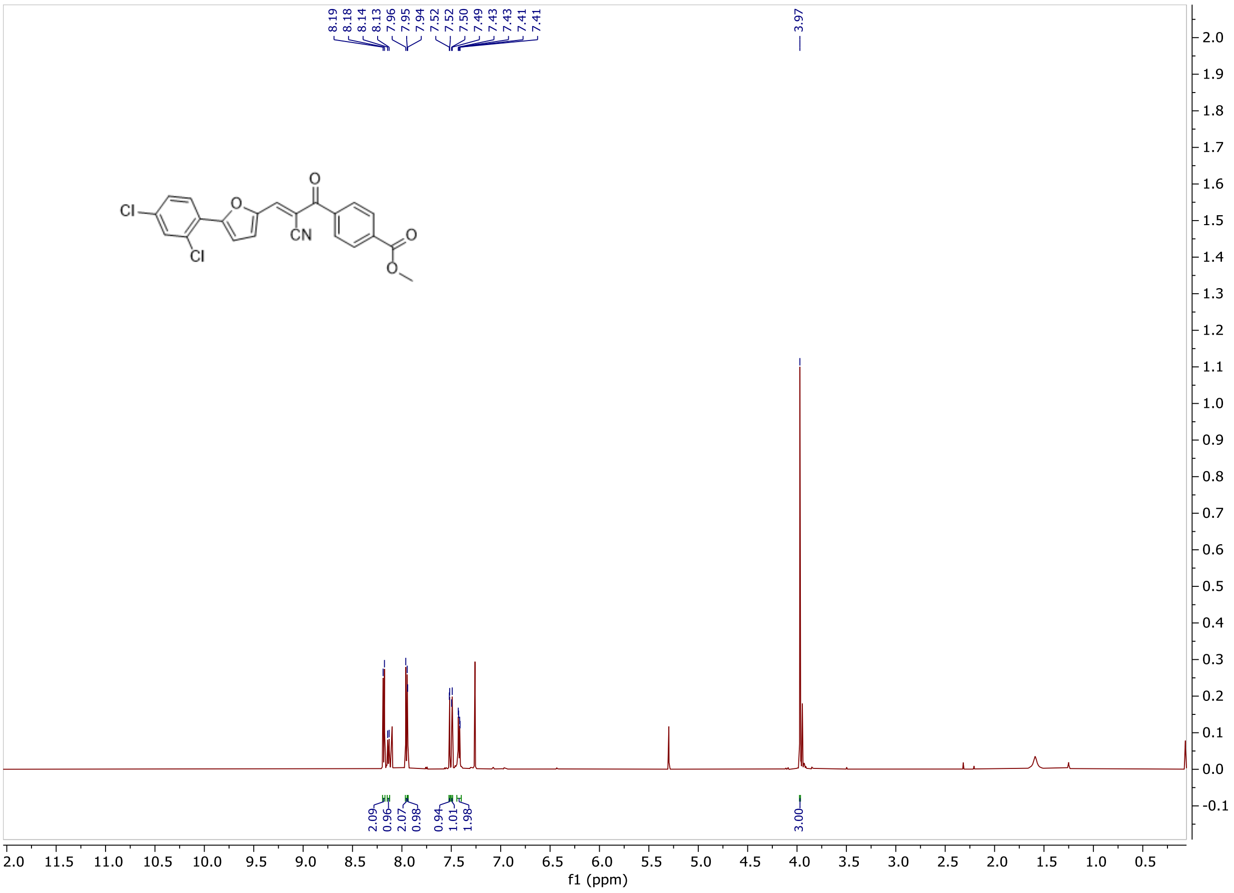

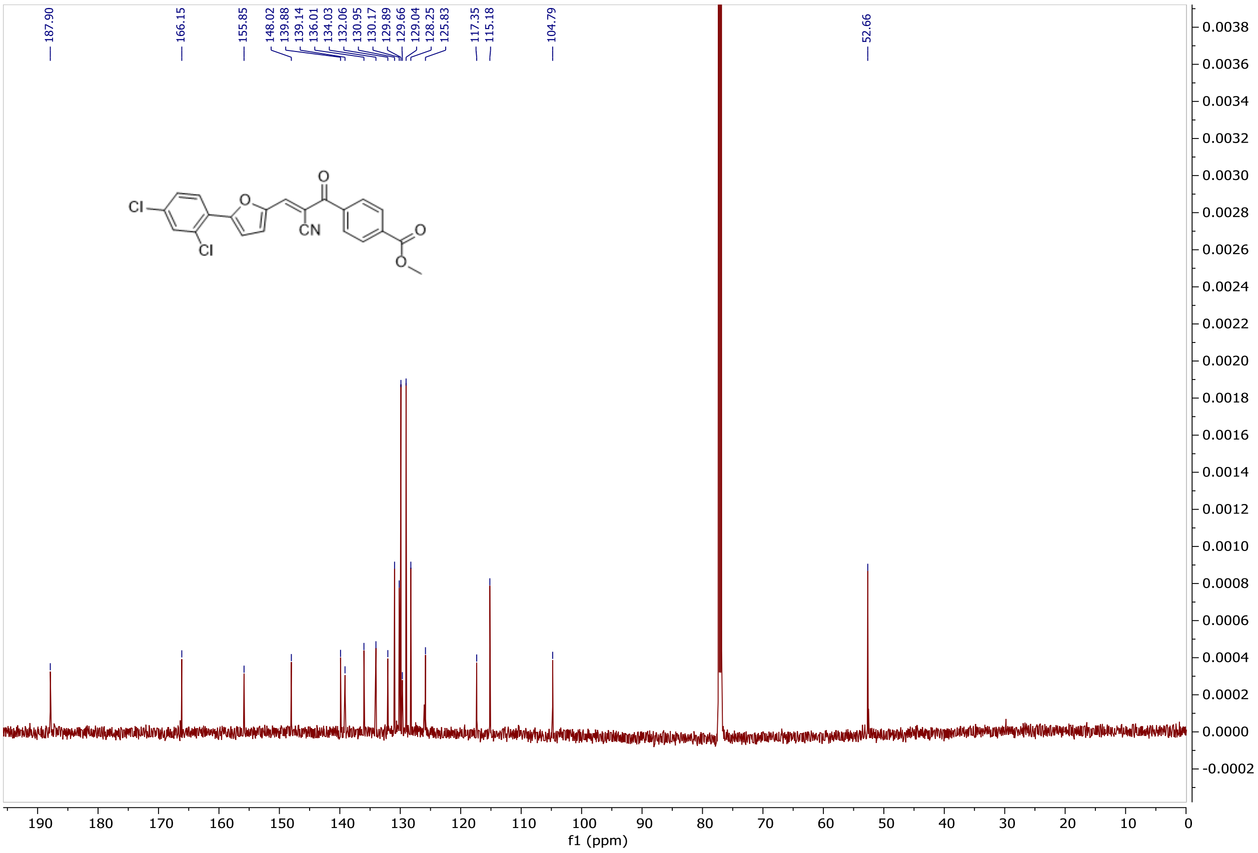

Supplementary Figure 39. ^{1}H-NMR (600 MHz, $CDCl_3$) and ^{13}C-NMR (151 MHz, $CDCl_3$) spectrum of **PB-3.7**.

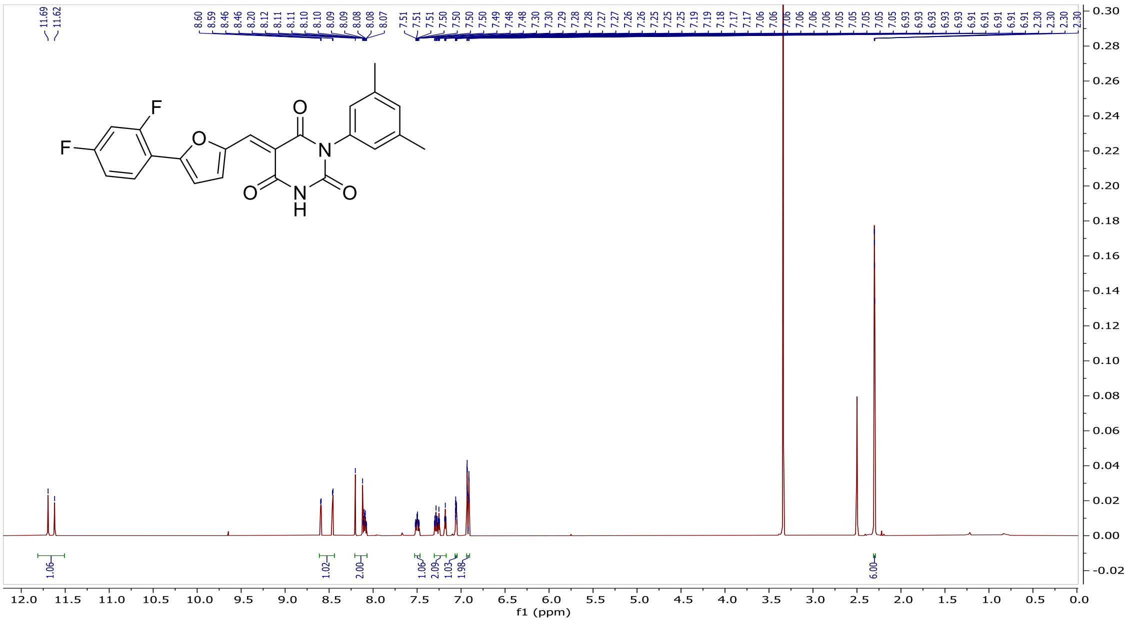

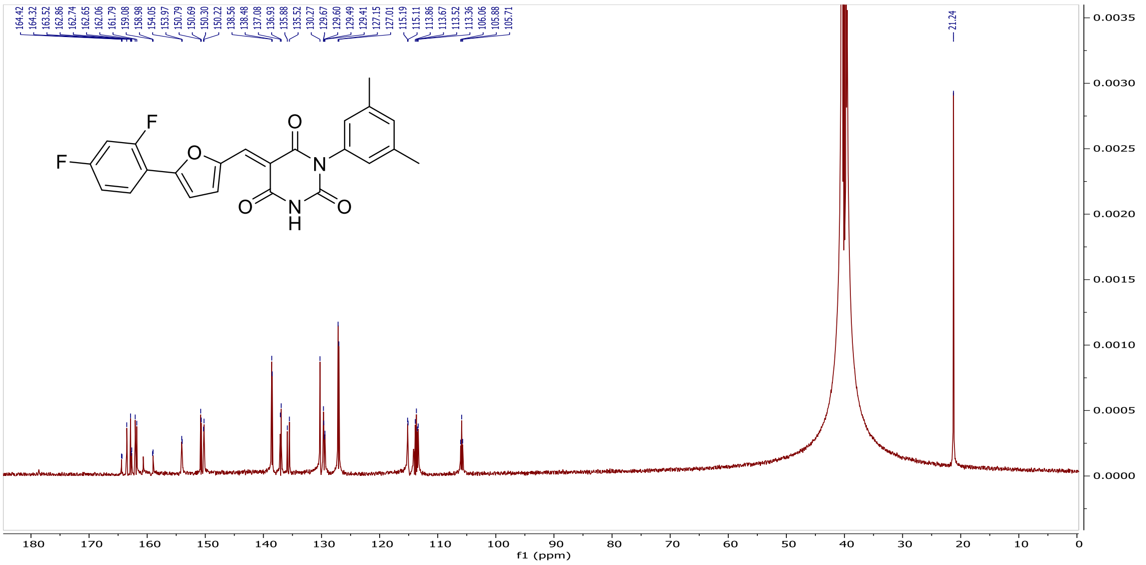

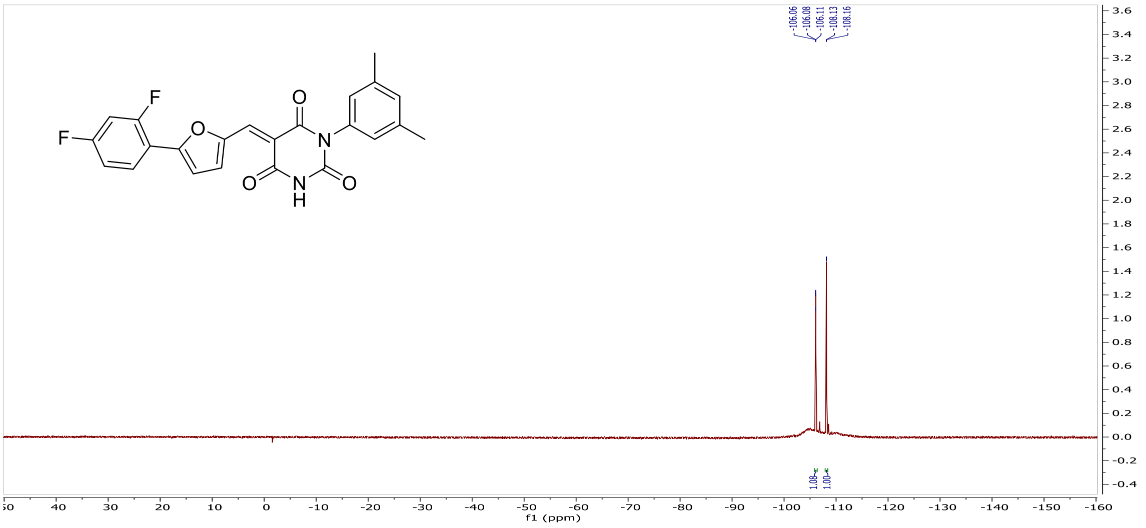

Supplementary Figure 40. ^{1}H NMR (600 MHz, DMSO-d$_6$) (top),^{13}C NMR (151 MHz, DMSO-d$_6$)(middle) and ^{19}F-NMR (376 MHz, DMSO-d$_6$) (bottom) spectrum of **PB-3.8**.

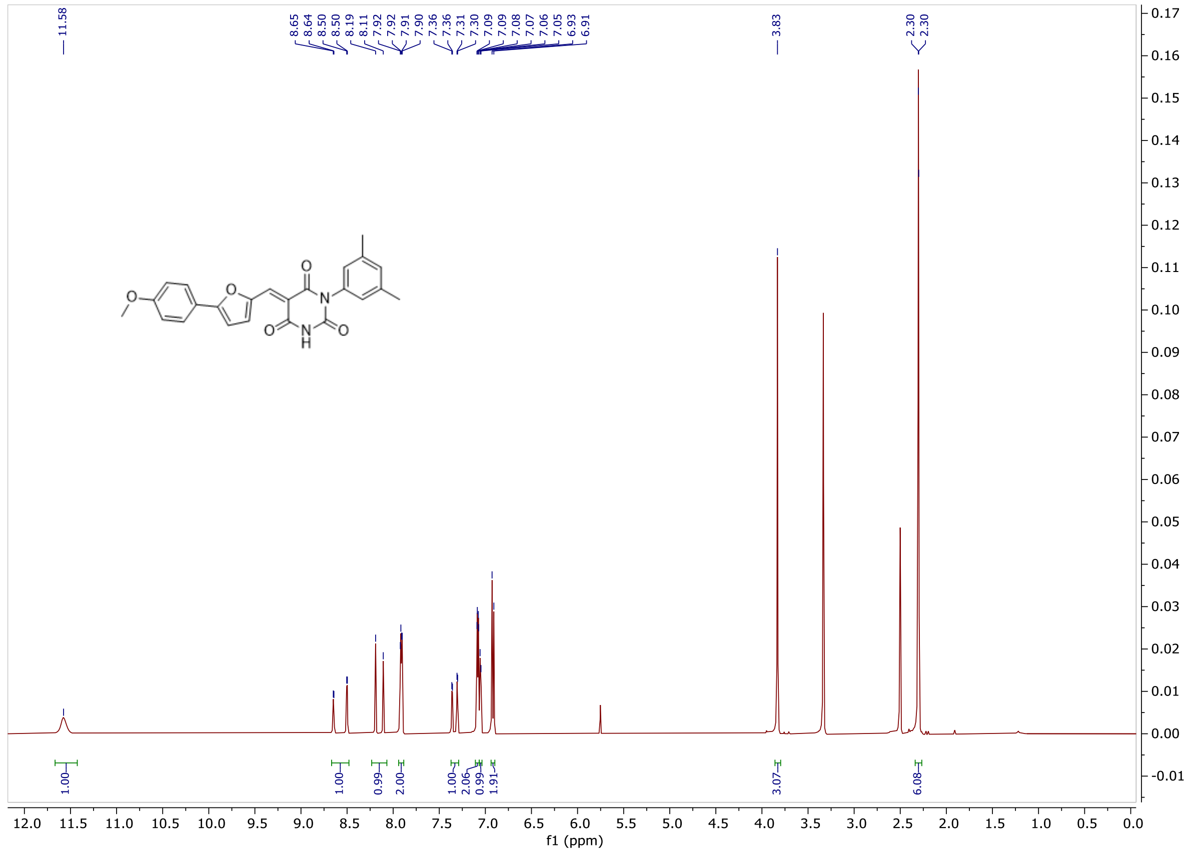

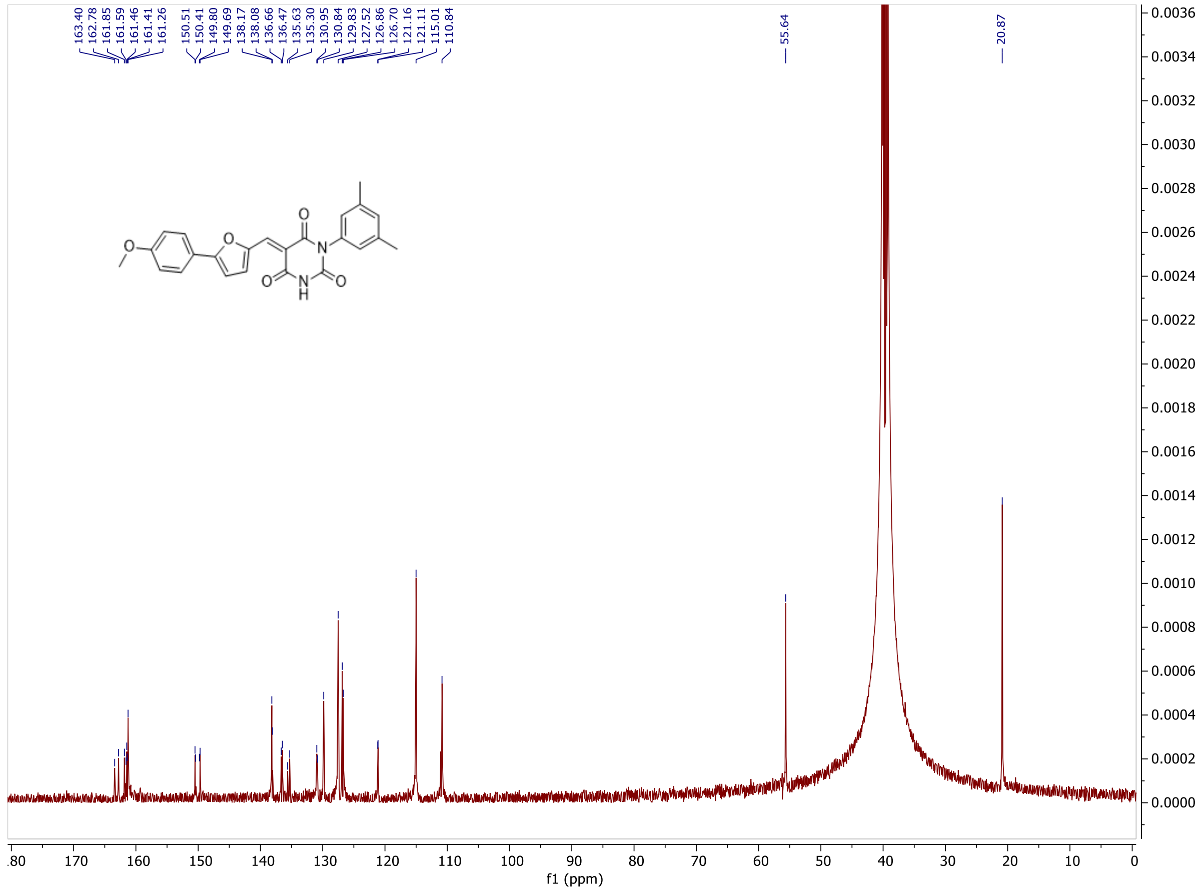

Supplementary Figure 41. ^{1}H NMR (600 MHz, DMSO-d_6) and ^{13}C NMR (151 MHz, DMSO-d_6) spectrum of **PB-3.9**.

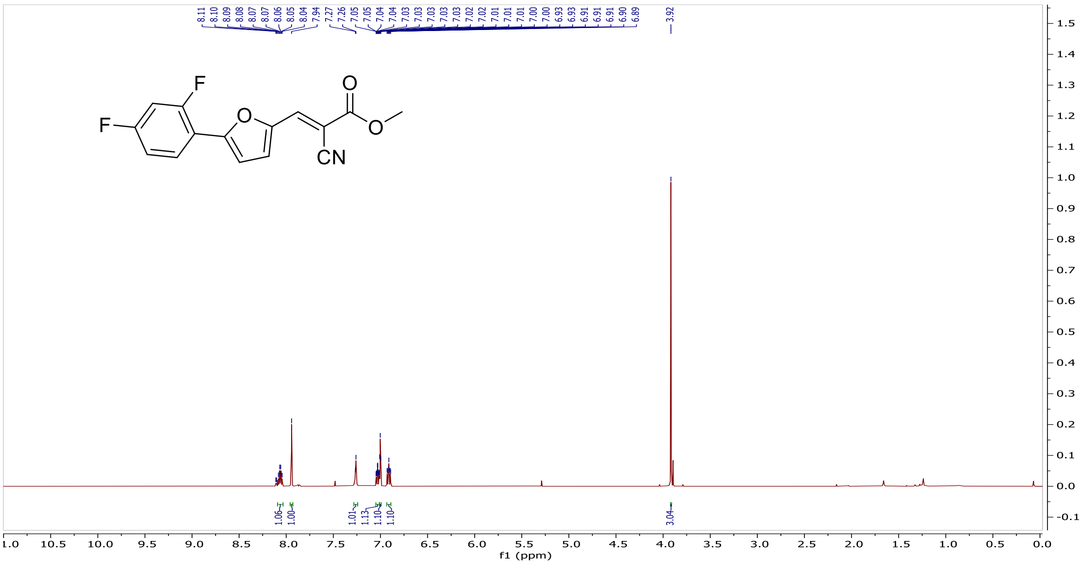

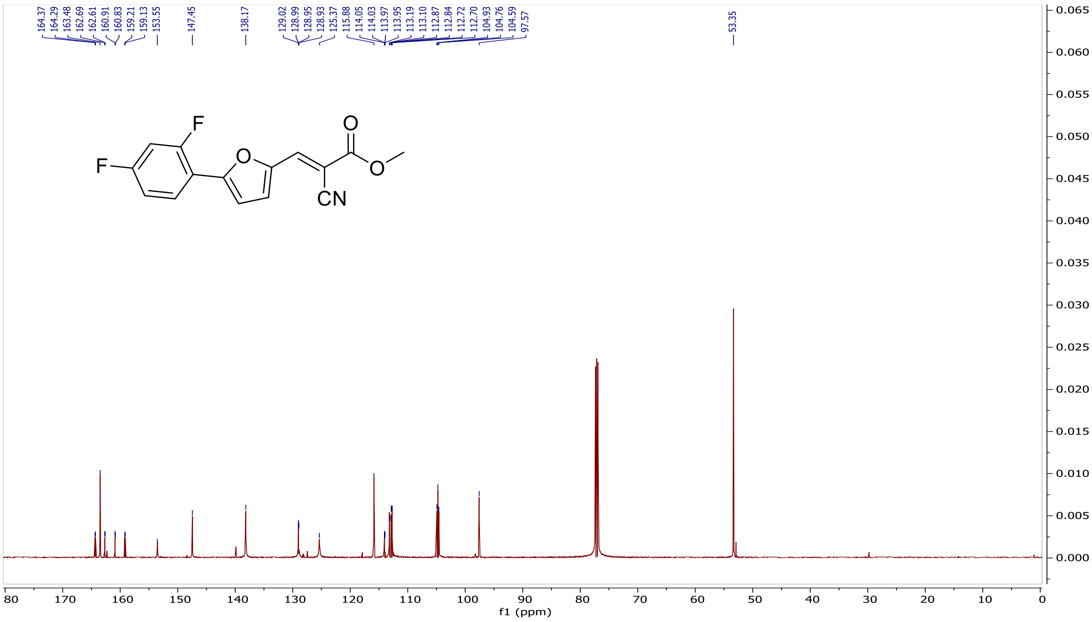

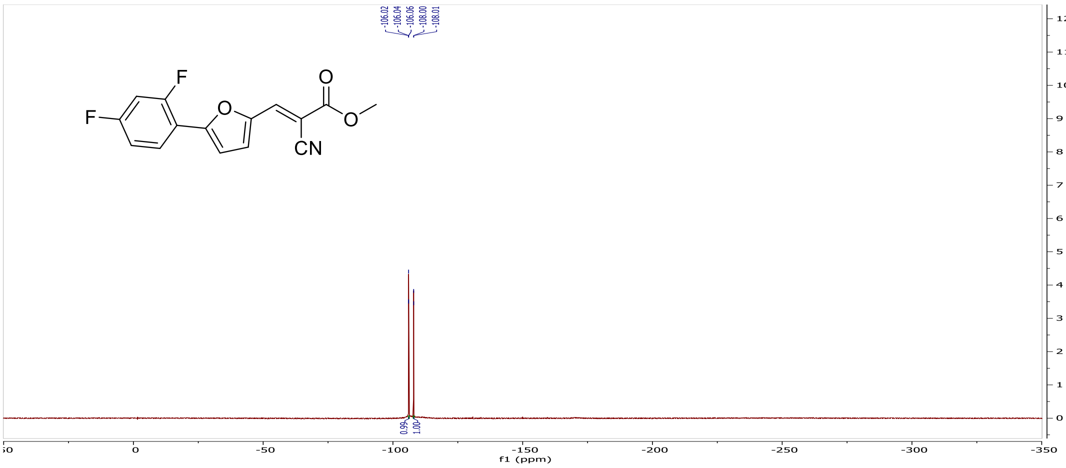

Supplementary Figure 42. ^1H NMR (600 MHz, $CDCl_3$) (top),^{13}C NMR (151 MHz, $CDCl_3$)(middle) and ^{19}F-NMR (376 MHz, $CDCl_3$) (bottom) spectrum of **PB-3.10**.

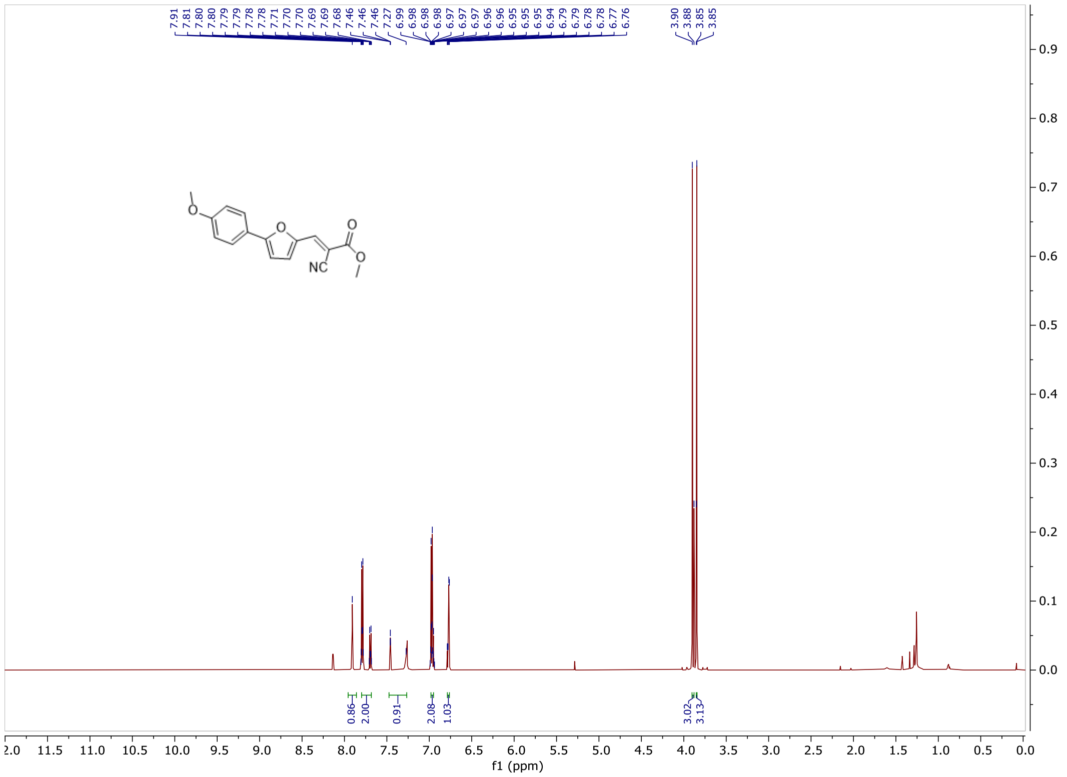

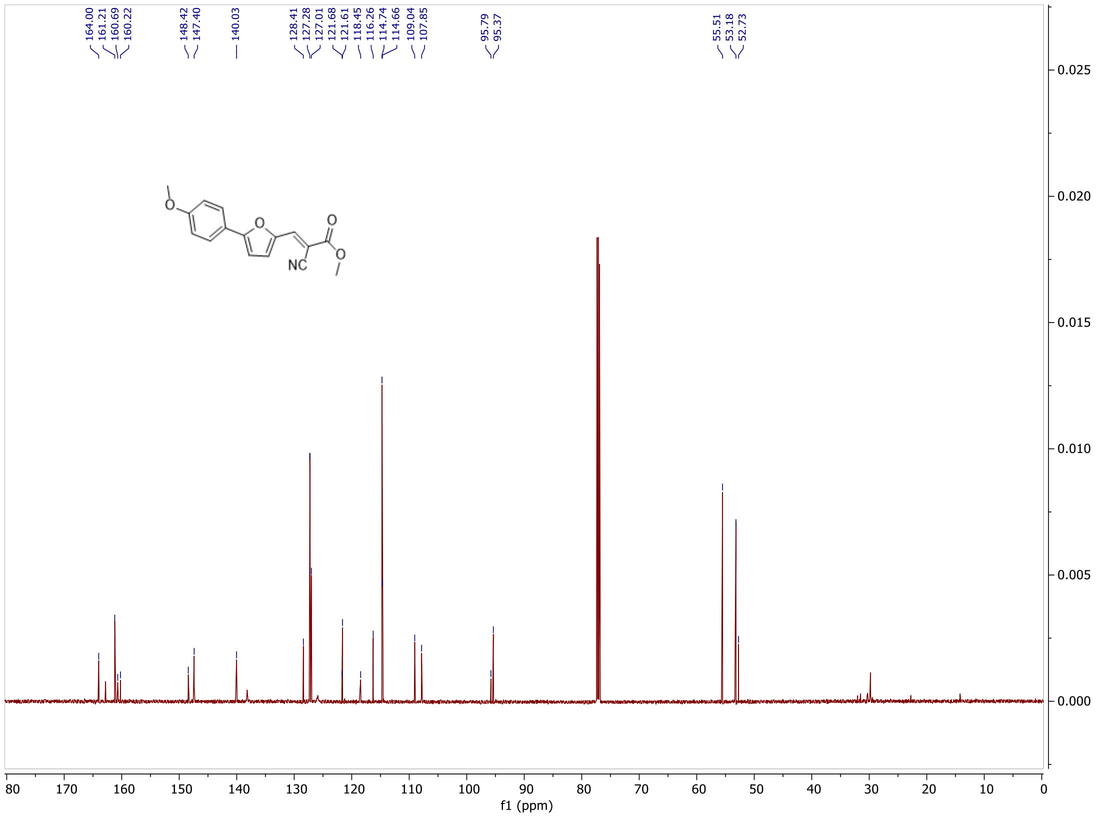

Supplementary Figure 43. ^{1}H NMR (600 MHz, $CDCl_3$) and ^{13}C NMR (151 MHz, $CDCl_3$) spectrum of **PB-3.11**.

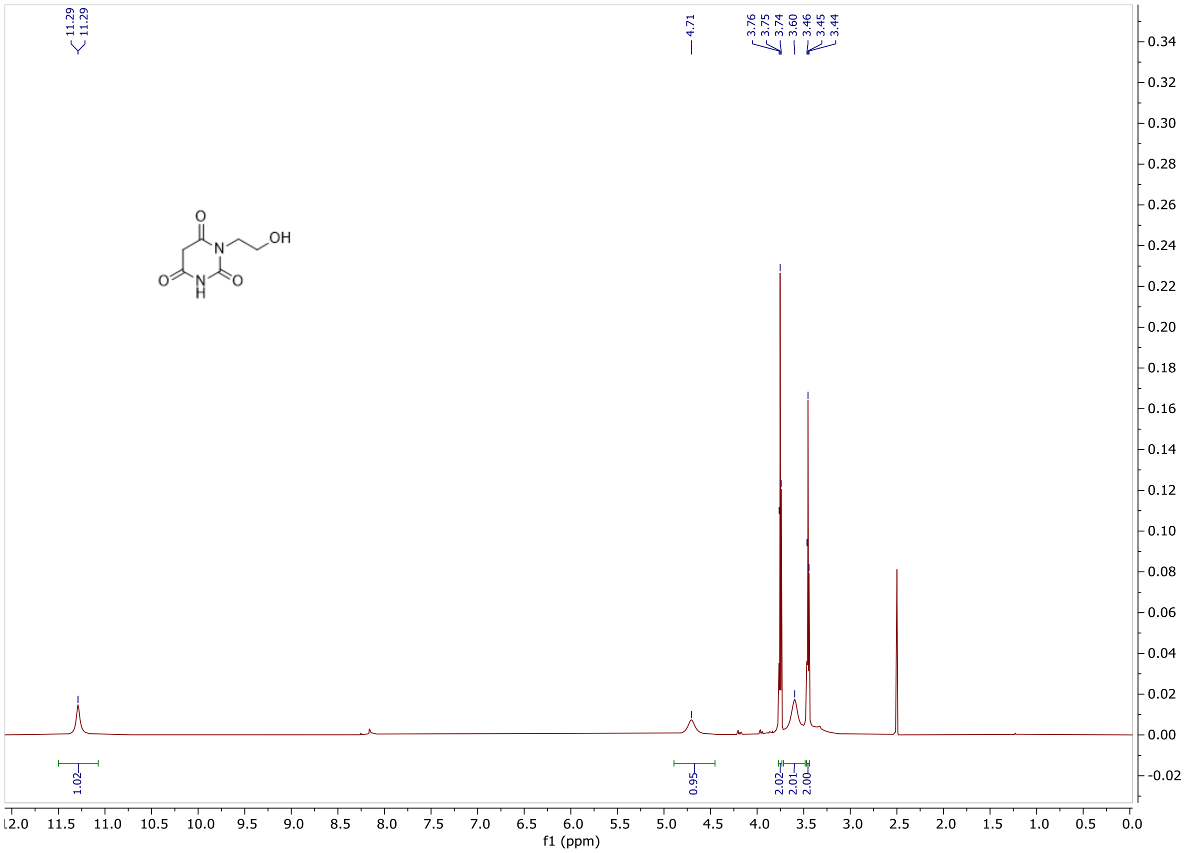

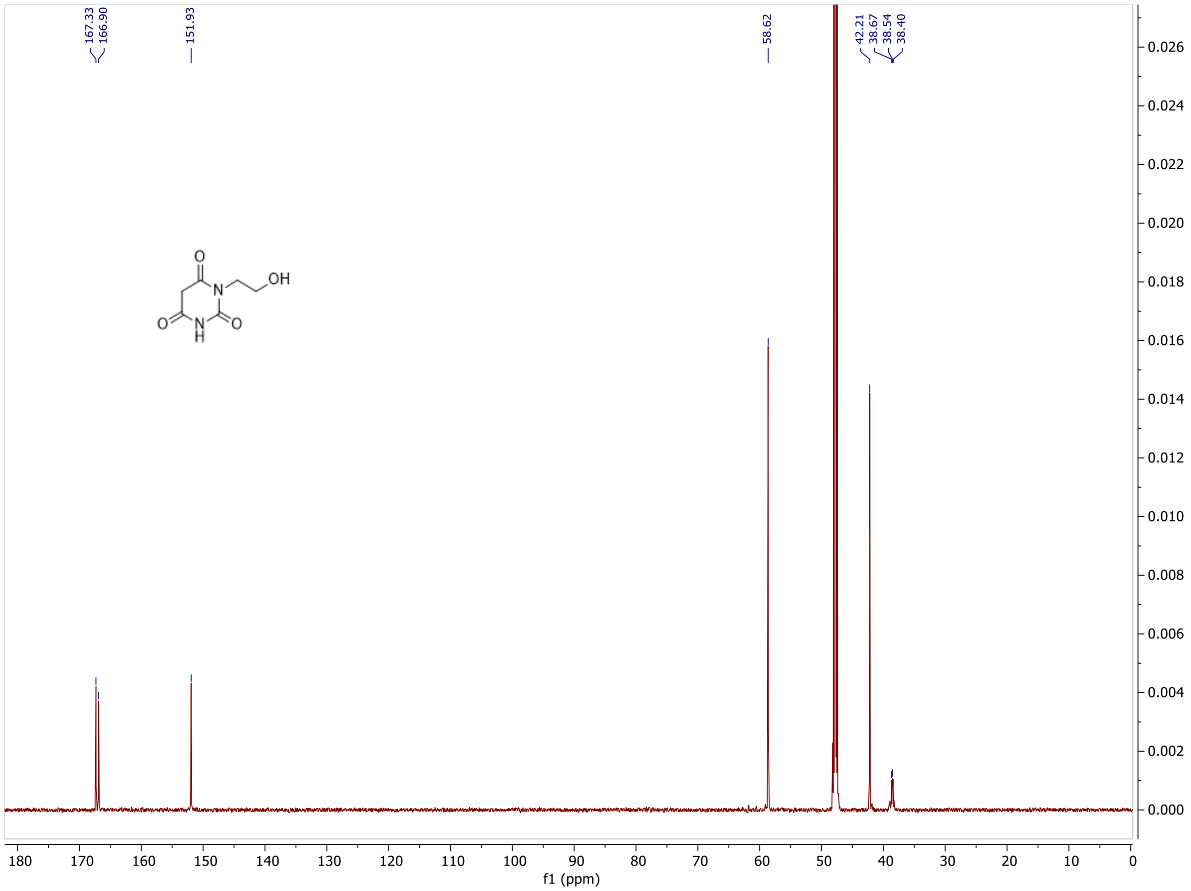

Supplementary Figure 44. ^{1}H NMR (600 MHz, DMSO-d_6) and ^{13}C NMR (151 MHz, CD_3OD) spectrum of **30**.

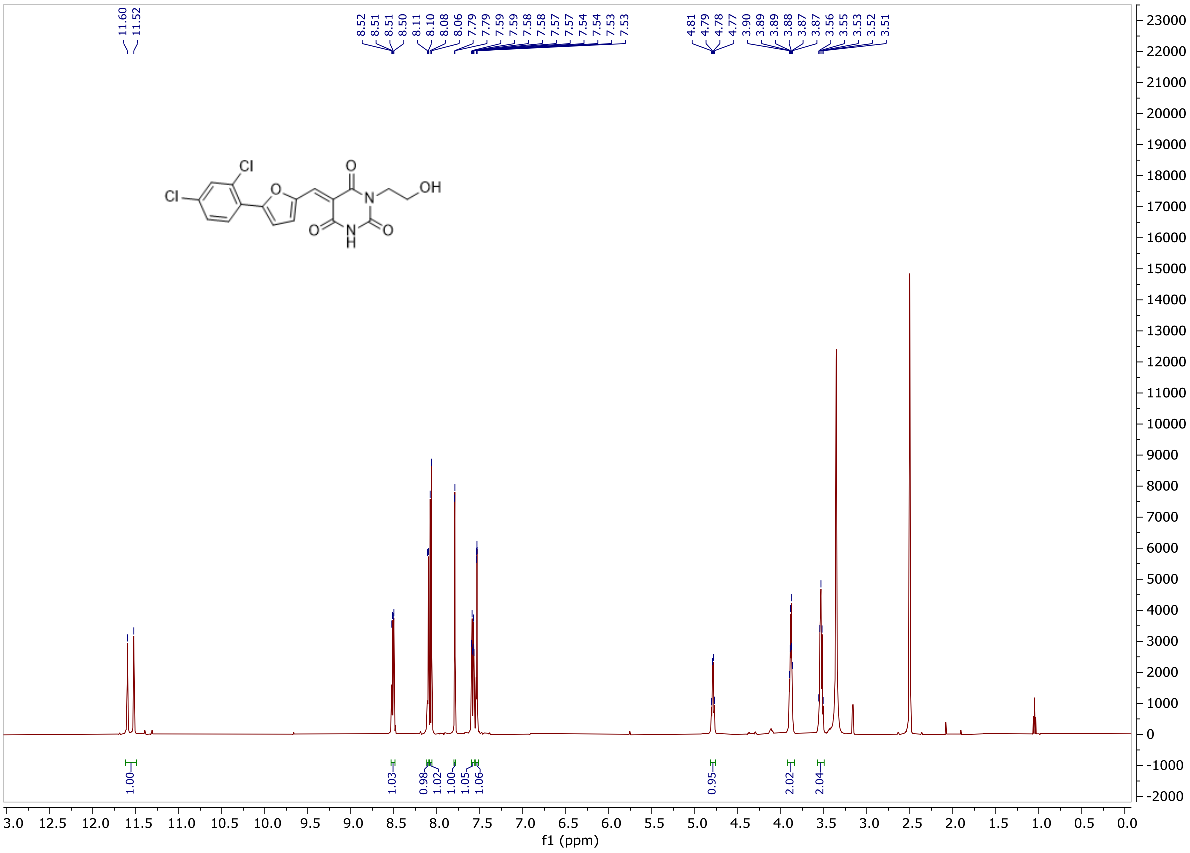

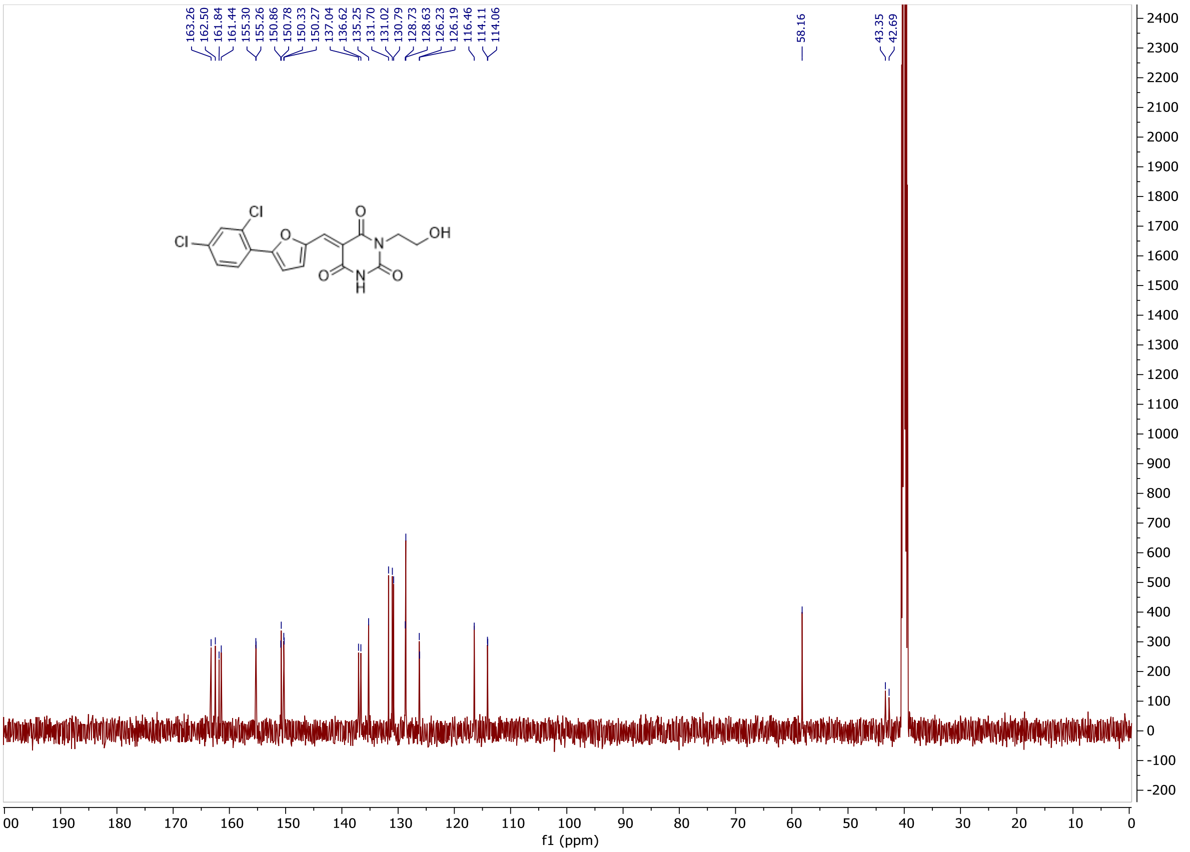

Supplementary Figure 45. ^{1}H NMR (500 MHz, DMSO-d_6) and ^{13}C NMR (151 MHz, DMSO-d_6) spectrum of **PB-3.12**.

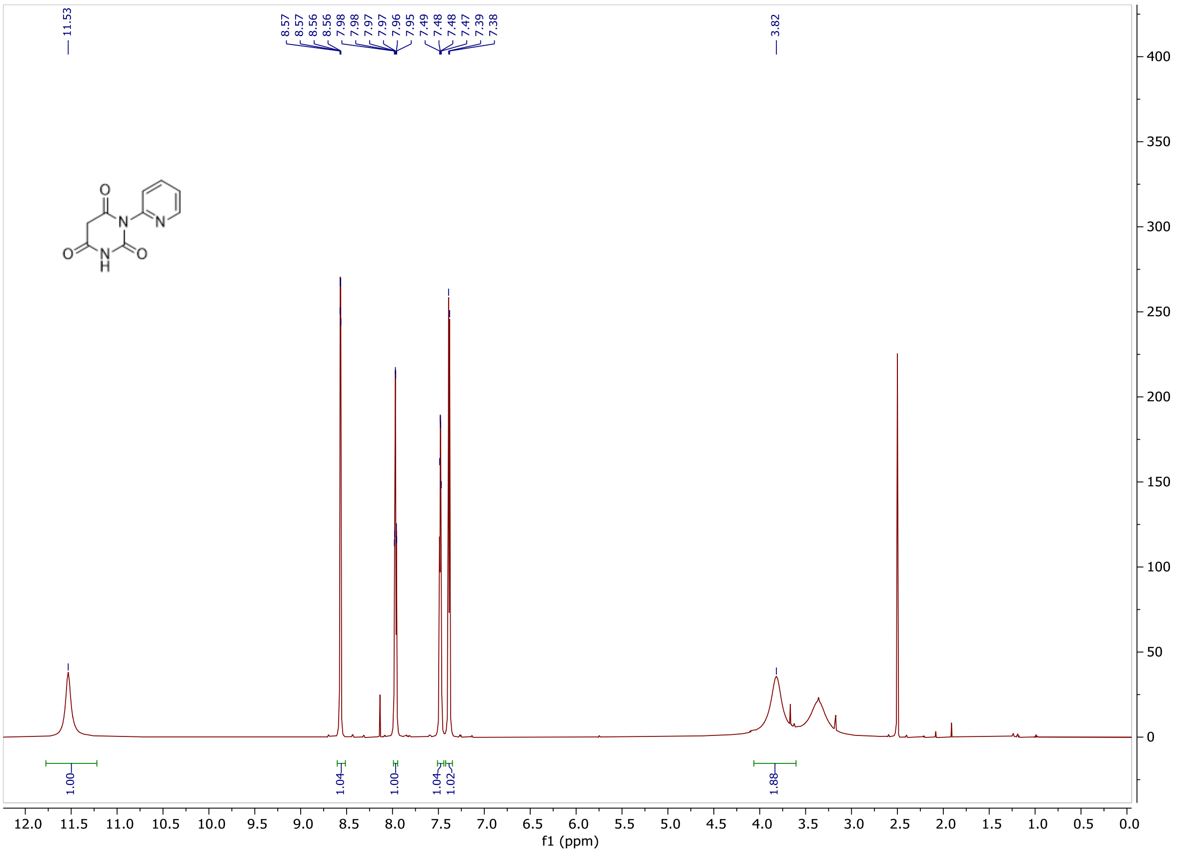

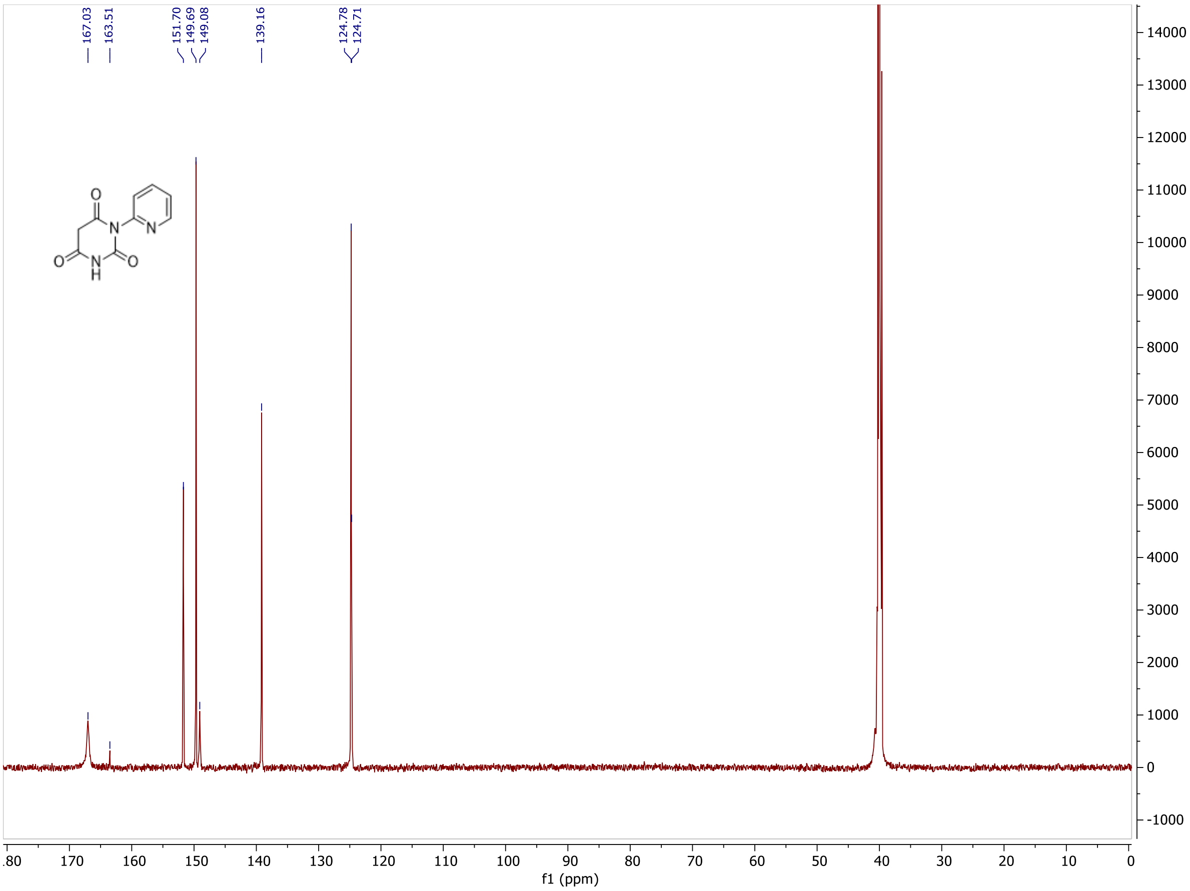

Supplementary Figure 46. ^{1}H NMR (700 MHz, DMSO-d_6) and ^{13}C NMR (176 MHz, DMSO-d_6) spectrum of **33.**

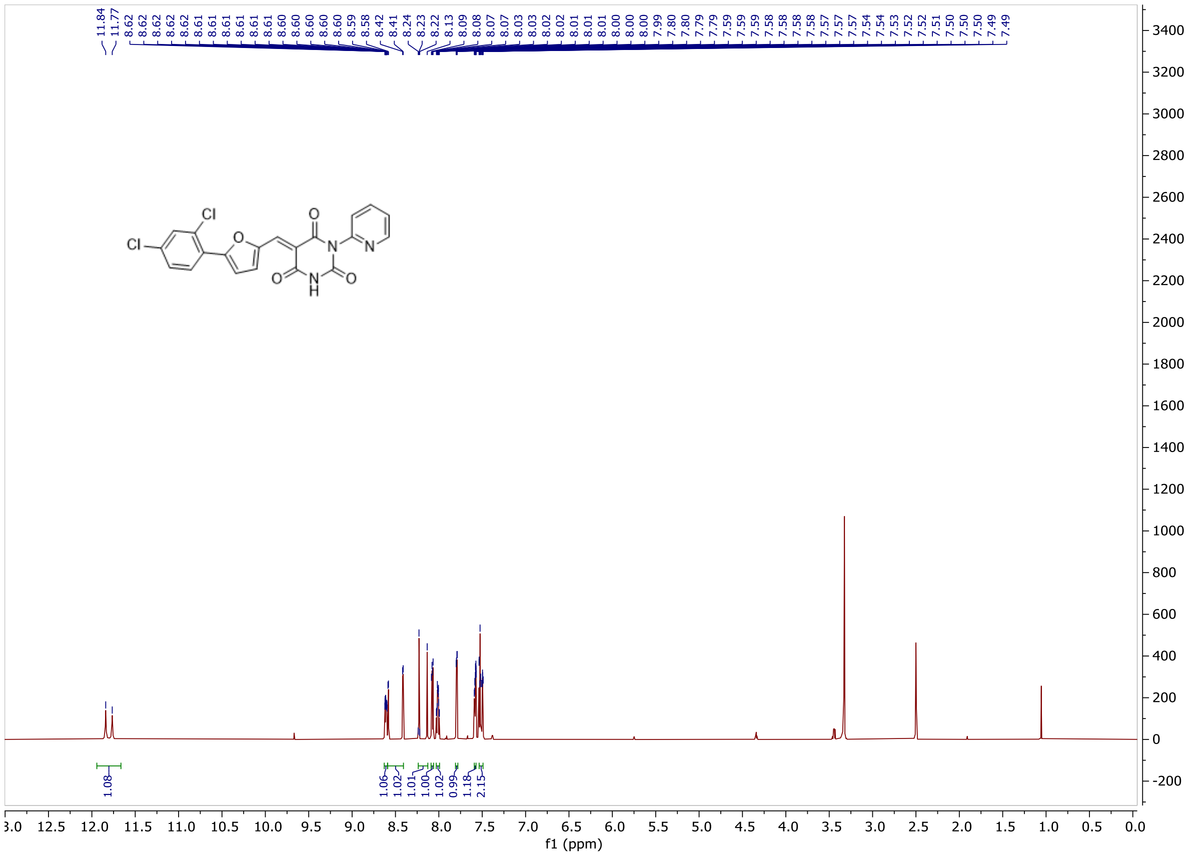

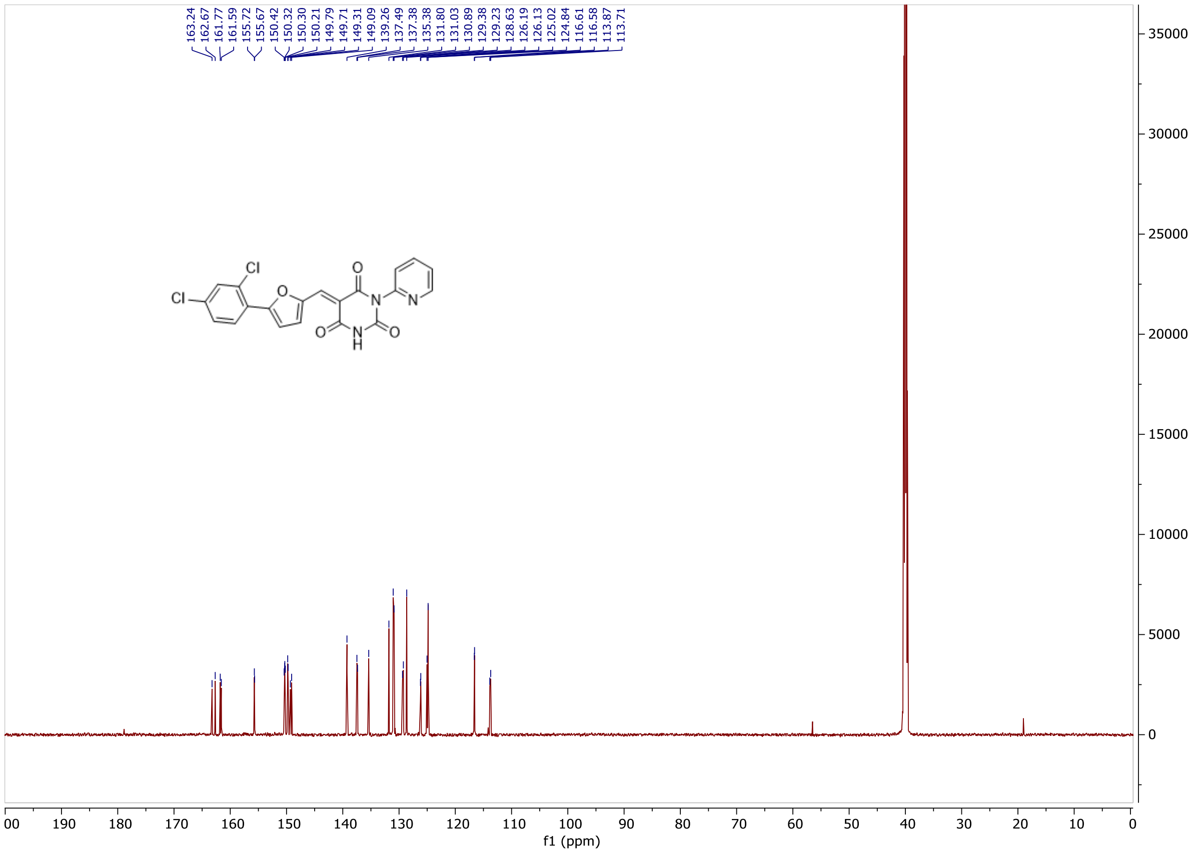

Supplementary figure 47. ^{1}H NMR (500 MHz, DMSO-d_6) and ^{13}C NMR (176 MHz, DMSO-d_6) spectrum of **PB-3.13**.

Supplementary Notes

Supplementary Note 1: Chemical Synthesis

5-(3-((2,4-Difluorophenoxy)-methyl)-4-methoxybenzylidene)-pyrimidine-2,4,6(1H,3H,5H)-trione (PB-1)

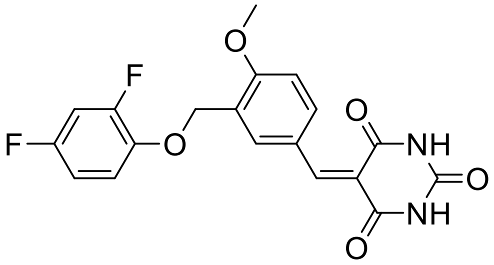

3-[(2,4-Difluorophenoxy)-methyl]-4-methoxybenzaldehyde (139 mg, 0.5 mmol) was dissolved in 10 ml of absolute ethanol. Next, barbituric acid (67 mg, 0.525 mmol) was added to solution. The reaction mixture was heated for 4 h at 100 °C, and then cooled to room temperature. The crystals of product **PB-1** were filtered off, washed twice with ethanol, and dried in *vacuo*.

Yield: 170 mg (87%)

Melting point: 261 °C

^{1}H NMR (600 MHz, DMSO-d_6) δ [ppm] = 11.31 (s, 1H), 11.18 (s, 1H), 8.45 (s, 1H), 8.42 (d, J = 8.9 Hz, 1H), 8.24 (s, 1H), 7.30 – 7.26 (m, 2H), 7.21 (d, J = 8.9 Hz, 1H), 7.00 (t, J = 10.9 Hz, 1H), 5.11 (s, 2H), 3.94 (s, 3H).

^{13}C NMR (151 MHz, DMSO-d_6) δ [ppm] = 163.84, 162.11, 161.22, 156.71 – 154.76 (m), 154.59, 151.67 (dd, J = 247.0, 12.8 Hz), 150.20, 143.04 (dd, J = 10.8, 3.6 Hz), 137.90, 136.49, 124.91, 124.10, 116.46 (dd, J = 9.9, 3.0 Hz), 116.03, 111.04, 110.85 (dd, J = 22.4, 4.3 Hz), 104.86 (dd, J = 27.2, 22.4 Hz), 66.40, 56.19.

^{19}F NMR (565 MHz, DMSO-d_6) δ [ppm] = -119.85, -129.36.

ESI-HRMS: [M+H$^+$] calculated for $C_{19}H_{15}F_2N_2O_5^+$: 389.0949 Da, found: 389.0944 m/z.

Elemental analysis: calculated for $C_{19}H_{14}F_2N_2O_5$: C=58.77%, H=3.63%, N=7.21%, found: C=58.98%, H=3.68%, N=7.25%.

NMR spectra for **PB-1** are in supplementary figure 22.

1-(3,5-Dimethylphenyl)-pyrimidine-2,4,6-(1H,3H,5H)-trione (PB-2.3)

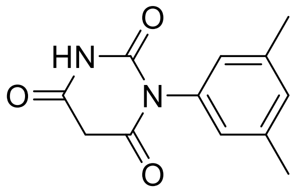

Sodium metal (287 mg, 12.5 mmol) was dissolved in absolute ethanol (15 ml). Next, N-(3,5-dimethylphenyl)-urea (821 mg, 5 mmol) and diethyl malonate (915 µl, 6 mmol) were added to the sodium ethoxide solution, and the reaction mixture was stirred at room temperature for about 10 min until a clear solution was formed. The mixture was heated under reflux for 6 h, and then it was stirred over night at room temperature. The solvent was removed by evaporation and water (15 ml) was added to residue. Further, 2 M HCl was added dropwise until pH 1-2 was reached and the solution was stirred for 0.5 h. The obtained white crystals of **PB-2.3** were filtered off, washed with water, and dried in high vacuum.

Yield: 1.07 g (92 %)

Melting point: 219-220 °C

^{1}**H NMR** (500 MHz, DMSO-d_6) δ [ppm] = 11.46 (s, 1H), 7.04 (s, 1H), 6.83 (s, 2H), 3.71 (s, 2H), 2.25 (s, 6H).

^{13}**C NMR** (126 MHz, DMSO-d_6) δ [ppm] = 166.75, 166.67, 151.55, 137.97, 134.85, 129.72, 126.39, 40.29, 20.68.

ESI-HRMS: [M+H$^+$] calculated for $C_{12}H_{13}N_2O_3^+$: 233.0926 Da, found: 233.0927 m/z.

Elemental analysis: [M+H_2O] calculated for $C_{12}H_{14}N_2O_4$: N=11.19 %, C=57.59 %, H=5.64 %, found: N=11.31 %, C=57.61 %, H=5.66 %.

NMR spectra for **PB-2.3** are in supplementary figure 23.

4-(2,4-Dichlorobenzyloxy)-3-ethoxybenzaldehyde (PB-2.2)

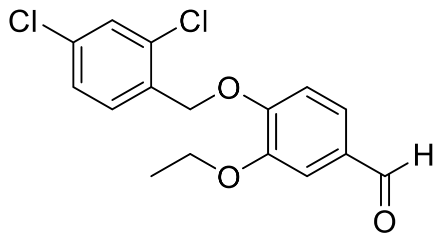

3-Ethoxy-4-hydroxybenzaldehyde (866 mg, 5.21 mmol) was dissolved in DMF (25 ml). Potassium carbonate (1440 mg, 10.42 mmol) was added to the solution and stirred for 1 h at room temperature. Next, 1-chloromethyl-2,4-dichloro-benzene (730 µl, 5.21 mmol) was added to the reaction mixture and heated for 5 h at 45 °C. The suspension was stirred overnight at room temperature, extracted with ethyl acetate (100 ml), washed with brine (50 ml) and water (50 ml). Organic phases were combined and dried with Na_2SO_4. Solvent was removed by evaporation and the semi-solid residue was dried in high vacuum to furnish product **PB-2.2**.

Yield: 1.66 g (99 %)

Melting point: 105 °C

^{1}H NMR (500 MHz, DMSO-d$_6$) δ [ppm] = 9.86 (d, *J* = 17.2 Hz, 1H), 7.67 (d, *J* = 18.0 Hz, 1H), 7.61 (d, *J* = 8.3 Hz, 1H), 7.54 (d, *J* = 9.2 Hz, 1H), 7.50 (d, *J* = 10.4 Hz, 1H), 7.43 (s, 1H), 7.26 (d, *J* = 8.1 Hz, 1H), 5.24 (d, *J* = 16.9 Hz, 2H), 4.10 (td, *J* = 15.4, 14.2, 7.6 Hz, 2H), 1.34 (p, *J* = 9.4, 8.8 Hz, 3H).

^{13}C NMR (126 MHz, DMSO-*d*$_6$) δ [ppm] = 191.41, 152.84, 148.65, 133.64, 133.46, 133.08, 131.07, 130.28, 128.94, 127.61, 125.52, 113.25, 111.38, 67.04, 64.02, 14.55.

ESI-HRMS: [M+H$^+$] calculated for $C_{16}H_{15}Cl_2O_3^+$: 325.0398 Da, found: 325.0396 m/z.

Elemental analysis: calculated for $C_{16}H_{14}Cl_2O_3$: C=59.10 %, H=4.34 %, found: C=59.75 %, H=4.34 %.

NMR spectra for **PB-2.2** are in supplementary figure 24.

(E/Z)-5-(4-(2,4-Dichlorobenzyloxy)-3-ethoxybenzylidene)-1-(3,5-dimethylphenyl)-pyrimidine-2,4,6(1H,3H,5H)-trione (PB-2)

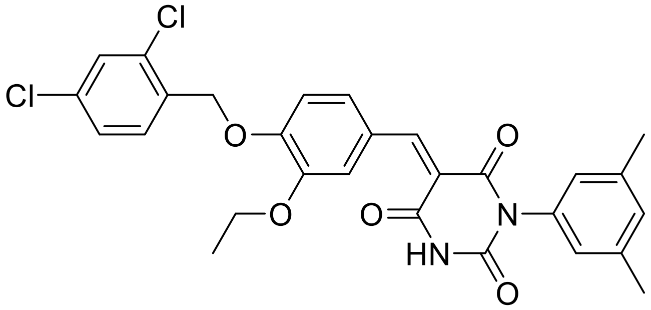

PB-2.2 (180 mg, 0.55 mmol) was dissolved in ethanol (9 ml) and water (1 ml). Next, **PB-2.3** was added to the solution which was heated under reflux until formation of precipitates. Further, ethanol (10 ml) was added and heated under reflux for 2 h. Yellow crystals were formed, which were filtered off and washed with ethanol and diethylether. Crystals were dried in high vacuum to furnish product **PB-2**.

Yield: 233 mg (79 %)

Melting point: 215 °C

^{1}H NMR (500 MHz, DMF-d_7) δ [ppm] = 11.64, 11.56 (2s, 1H), 8.57, 8.41 (2s, 1H), 8.41, 8.30 (2s, 1H), 8.0-7.9 (m, 1H), 7.8 – 7.7 (m, 2H), 7.55 (m, 1H), 7.4 – 7.3 (m, 1H), 7.2-7.0 (m, 3H), 5.38, 5.36 (2s, 2H), 4.24 – 4.08 (2q, 2H), 2.33 (s, 6H), 1.43 – 1.30 (2t, 3H).

^{13}C NMR (126 MHz, DMF-*d_7*) δ [ppm] = 164.28, 162.00, 156.65, 156.10, 153.23, 153.15, 150.85, 150.74, 148.28, 138.63, 138.60, 136.32, 136.09, 134.46, 134.20, 134.16, 133.84, 131.79, 131.62, 131.56, 131.28, 130.02, 129.43, 129.41, 128.03, 128.01, 127.15, 127.01, 126.92, 126.77, 119.38, 118.99, 116.81, 116.65, 113.27, 67.69, 64.64, 20.68, 14.59, 14.54.

ESI-HRMS: [M+H$^+$] calculated for $C_{28}H_{25}Cl_2N_2O_5^+$: 539.1141 Da, found: 539.1133 m/z.

Elemental analysis: calculated for $C_{28}H_{24}Cl_2N_2O_5$: C=62.35 %, H=4.48 %, N=5.19 %, found: C=62.35 %, H=4.497 %, N=5.193 %.

NMR spectra for **PB-2** are in supplementary figure 25.

5-(4-(2,4-Dichlorobenzyloxy)-3-ethoxybenzyl)-1-(3,5-dimethylphenyl)-pyrimidine-2,4,6(1H,3H,5H)-trione (PB-2.1)

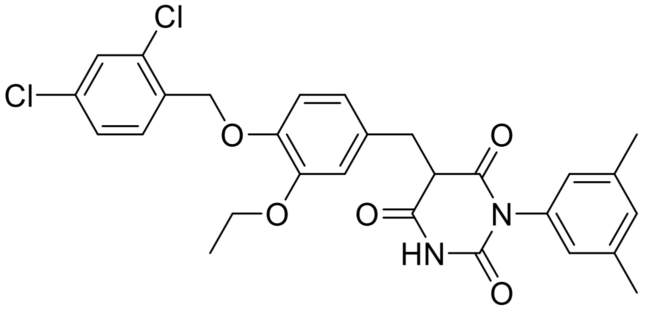

PB-2 (100 mg, 0.185 mmol) was suspended in ethanol (5 ml) and sodium borohydride (21 mg, 0.556 mmol) was added. The mixture was stirred for 45 min. Next, 2 M HCl was added until a pH 2 was reached. The reaction mixture was concentrated *in vacuo* and water (5 ml) was added. Further, the mixture was extracted 3 times with ethyl acetate (10 ml). Organic phases combined and dried with Na_2SO_4. Solvents were removed and the obtained greasy residue was dried in high vacuum to furnish **PB-2.1** as a colorless oil.

Yield: 82 mg (82 %)

^{1}H NMR (500 MHz, Chloroform-d) δ [ppm] = 7.98 (s, 1H), 7.53 (d, *J* = 8.3 Hz, 1H), 7.40 (s, 1H), 7.05 (s, 1H),6.82 (d, *J* = 7.9 Hz, 1H), 6.72 (s, 1H), 6.69 (d, *J* = 8.6 Hz, 1H), 6.51 (s, 2H), 5.18 (s, 2H), 4.02 (q, *J* = 7.5Hz, 2H), 3.86 (s, 1H), 3.55 – 3.46 (m, 2H), 2.31 (s, 6H), 1.43 (q, *J* = 6.6, 5.3 Hz, 3H).

^{13}C NMR (126 MHz, Chloroform-*d*) δ [ppm] = 168.73, 167.87, 149.50, 149.32, 147.85, 139.44, 134.10, 133.75, 133.09, 132.95, 131.40, 129.59, 129.22, 128.95, 127.47, 125.87, 121.88, 115.30, 115.06, 68.10, 64.74, 50.87, 36.84, 21.33, 14.99.

ESI-HRMS: [M+H$^+$] calculated for $C_{28}H_{27}Cl_2N_2O_5^+$: 541.1297 Da, found: 541.1290 m/z.

Elemental analysis: calculated for $C_{28}H_{26}Cl_2N_2O_5$: C=62.12 %, H=4.84 %, N=5.17 %, found: C=62.28 %, H=4.885 %, N=5.266 %.

NMR spectra for **PB-2.1** are in supplementary figure 26.

(E/Z)-1-(3,5-Dimethylphenyl)-5-(3-ethoxy-4-hydroxybenzylidene)-pyrimidine-2,4,6(1H,3H,5H)-trione (PB-2.4)

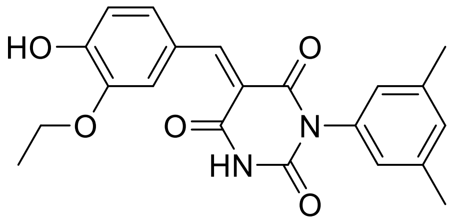

3-Ethoxy-4-hydroxybenzaldehyde (44.6 mg, 0.27 mmol) was dissolved in ethanol (5 ml) and **PB-2.3** (58.1 mg, 0.25 mmol) was added to the solution. Next, the mixture was heated for 5 h under reflux and cooled down to room temperature, then stirred overnight at room temperature. Yellow crystals were filtered off and washed with diethyl ether and ethanol. Finally, crystals were dried in high vacuum to furnish product **PB-2.4**.

Yield: 75 mg (79 %)

Melting point: 251 °C

^{1}H NMR (500 MHz, DMSO-d_6) δ [ppm] = 11.56, 11.45 (2s, 1H), 10.51, 10.47 (2s, 1H), 8.41 (d, *J* =97.3 Hz, 1H), 8.20 (d, *J* = 17.5 Hz, 1H), 7.89 – 7.87 (m, 1H), 7.04 (s, 1H), 6.90 (t, *J* = 9.0 Hz,3H), 4.13 – 3.99 (2q, 2H), 2.30 (s, 6H), 1.40 – 1.30 (2t, 3H).

^{13}C NMR (126 MHz, DMSO-d_6) δ [ppm] = 164.41, 162.32, 153.98, 150.75, 146.72, 142.86, 138.47, 136.10, 130.19, 127.25, 127.09, 120.00, 119.43, 115.99, 114.89, 64.48, 21.28, 15.16.

ESI-HRMS: [M+H$^+$] calculated for $C_{21}H_{21}N_2O_5^+$: 381.1450 Da, found: 381.1445 m/z.

Elemental analysis: calculated for $C_{21}H_{20}N_2O_5$: C=66.31 %, H=5.30 %, N=7.36 %, found: C=66.43 %, H=5.327 %, N=7.366 %.

NMR spectra for **PB-2.4** are in supplementary figure 27.

5-(4-(2,4-Dichlorobenzyloxy)-3-ethoxybenzylidene)-pyrimidine-2,4,6(1H,3H,5H)-trione (PB-2.5)

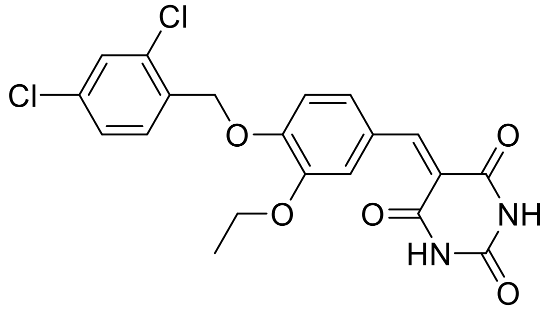

PB-2.2 (84.8 mg, 0.26 mmol) was dissolved in ethanol (5 ml) and pyrimidine-2,4,6-(1H,3H,5H)-trione (35.5 mg, 0.277 mmol) was added. The mixture was heated for 2 h under reflux and cooled down to room temperature. Yellow crystals were filtered and washed with diethylether and ethanol. Finally, crystals were dried in high vacuum to furnish product **PB-2.5**.

Yield: 88 mg (78 %)

Melting point: 253 °C

^{1}H NMR (500 MHz, DMF-d_7) δ [ppm] = 11.39, 11.26 (2s, 2H), 8.55 (s, 1H), 8.37 (s, 1H), 7.93 (d, *J* = 8.6Hz, 1H), 7.75 (d, *J* = 9.0 Hz, 1H), 7.70 (s, 1H), 7.55 (d, *J* = 9.1 Hz, 1H), 7.33, 7.31 (2s, 1H), 5.38 (s,2H), 4.25 – 4.12 (m, 2H), 1.47 – 1.33 (m, 3H).

^{13}C NMR (126 MHz, DMF-*d_7*) δ [ppm] = 164.36, 162.78, 155.97, 153.14, 150.62, 148.23, 134.43, 134.17, 133.83, 131.68, 131.59, 129.40, 128.01, 126.79, 119.01, 116.18, 113.23, 67.67, 64.60, 14.56.

ESI-HRMS: [M+H$^+$] calculated for $C_{20}H_{17}Cl_2N_2O_5^+$: 435.0515 Da, found: 435.0540 m/z.

Elemental analysis: calculated for $C_{20}H_{16}Cl_2N_2O_5$: C=55.19 %, H=3.71 %, N=6.44 %, found: C=55.24 %, H=3.745 %, N=6.968 %.

NMR spectra for **PB-2.5** are in supplementary figure 28.

(E/Z)-5-(3-(3,5-Dichlorophenoxy)-benzylidene)-1-(3,5-dimethylphenyl)-pyrimidine-2,4,6-(1H,3H,5H)-trione (PB-2.6)

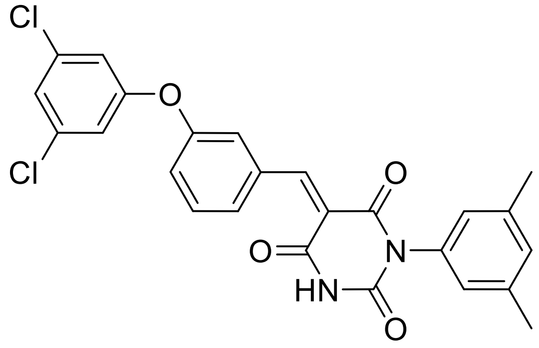

3-(3,5-Dichlorophenoxy)-benzaldehyde (67.6 mg, 0.253 mmol) and **PB-2.3** were dissolved in absoluteethanol (10 ml). The reaction mixture was heated under reflux for 3 h and cooled down to room temperature. Concentrated reaction mixture (2-3 ml) was heated again under reflux for 6 h and stirred overnight at room temperature. Crystals of product **PB-2.6** were obtained by filtration, washed with diethylether and were dried under vacuum.

Yield: 50 mg (41 %)

Melting point: 230 °C

^{1}H NMR (500 MHz, DMSO-d$_6$) δ [ppm] = 11.74, 11.58 (2s, 1H), 8.38, 8.38 (2s, 1H), 7.94 – 7.88 (m, 1H), 7.82 – 7.75 (m, 1H), 7.59 – 7.47 (m, 1H), 7.36 (d, *J* = 31.5 Hz, 1H), 7.32 – 7.24 (m, 1H), 7.11 (s, 1H), 7.06 – 6.99 (m, 2H), 6.89 (s, 2H), 2.29, 2.27 (2s, 6H).

^{13}C NMR (126 MHz, DMSO-*d*$_6$) δ [ppm] = 160.90, 160.73, 158.26, 154.37, 154.12, 153.73, 153.57, 150.22, 150.11, 137.95, 137.86, 135.21, 135.00, 134.96, 134.90, 130.06, 129.94, 129.76, 129.62, 129.51, 129.21, 126.47, 126.38, 123.16, 123.09, 123.02, 120.63, 120.54, 117.09, 116.88, 20.69, 20.68.

ESI-HRMS: [M+H$^+$] calculated for $C_{25}H_{19}Cl_2N_2O_4^+$: 481.0722 Da, found: 481.0713 m/z.

Elemental analysis: calculated for $C_{25}H_{18}Cl_2N_2O_4$: C=62.38 %, H=3.77 %, N=5.82 %, found: C=62.67 %, H=3.837 %, N=5.823 %.

NMR spectra for **PB-2.6** are in supplementary figure 29.

(E/Z)-5-(4-((2,4-Dichlorobenzyl)-oxy)-benzylidene)-1-(3,5-dimethylphenyl)-pyrimidine-2,4,6-(1H,3H,5H)-trione (PB-2.8)

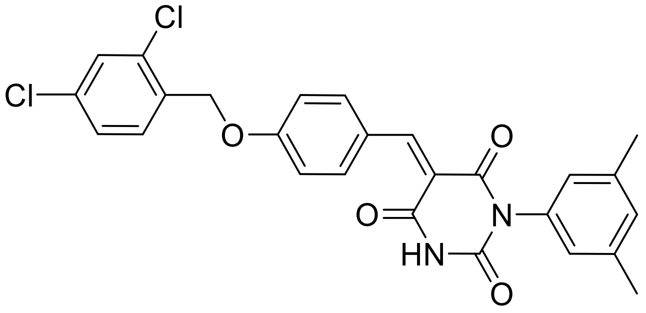

4-((2,4-Dichlorobenzyl)-oxy)-benzaldehyde (72.7 mg, 0.259 mmol) was dissolved in absolute ethanol (10 ml) and **PB-2.3** (60.5 mg, 0.260 mmol) was added to the solution. The mixture was heated under reflux for 5 h and cooled down to room temperature. The suspension was stirred overnight. Yellow crystals were obtained by filtration and washed with ethanol and diethylether. In the end, crystals of product **PB-2.8** were dried in vacuo.

Yield: 87 mg (68 %)

^{1}H NMR (500 MHz, DMF-d_7) δ [ppm] = 11.66, 11.57 (2s, 1H),8.53 – 8.37 (m, 3H), 7.81 – 7.67 (m, 2H), 7.54 (s, 1H), 7.27(d, *J* = 9.9 Hz, 1H), 7.22 (d, *J* = 8.0 Hz, 1H), 7.08 (s, 1H),7.04 (s, 2H), 5.38 – 5.35 (q, 2H), 2.34 (s, 6H).

^{13}C NMR (126 MHz, DMF-*d_7*) δ [ppm] = 164.19, 163.32, 162.89, 161.84, 156.09, 155.65, 150.84, 138.61, 137.93, 137.87, 136.30, 136.04, 134.40, 133.64, 131.86, 129.99, 129.49, 128.05, 127.16, 127.00, 116.88, 114.98, 67.28, 20.67.

ESI-HRMS: [M+H$^+$] calculated for $C_{26}H_{21}Cl_2N_2O_4^+$: 495.0878 Da, found: 495.0874 m/z.

Elemental analysis: calculated for $C_{26}H_{20}Cl_2N_2O_4$: C=63.04 %, H=4.07 %, N=5.66 %, found: C=63.20 %, H=4.078 %, N=5.738 %.

NMR spectra for **PB-2.8** are in supplementary figure 30.

(E/Z)-5-(4-(Benzyloxy)-3-ethoxybenzylidene)-1-phenylpyrimidine-2,4,6(1H,3H,5H)-trione (PB-2.10)

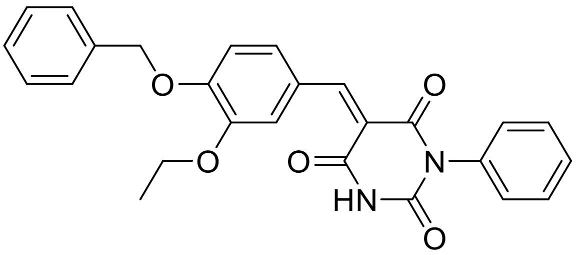

In a solution of 4-(benzyloxy)-3-ethoxybenzaldehyde (128 mg, 0.5 mmol) in ethanol (20 ml) 1-phenylpyrimidine-2,4,6(1H,3H,5H)-trione (102 mg, 0.5 mmol) was added. The mixture was stirred for overnight at room temperature. Crystals were collected by filtration, washed with ethanol and diethylether, and dried under vacuum.

Yield: 139 mg (63 %)

Melting point: 220 °C

^{1}H NMR (500 MHz, DMF-d_7) δ [ppm] = 11.66, 11.55 (2s, 1H), 8.45, 8.22 (2s, 1H), 7.98 – 7.93 (m, 1H), 7.58 – 7.37 (m, 10H), 7.32 – 7.26 (m, 2H), 5.35, 5.32 (2s, 2H), 4.24 – 4.07 (2q, 2H), 1.43 – 1.32 (2t, 3H).

^{13}C NMR (126 MHz, DMF-*d_7*) δ [ppm] = 164.18, 156.72, 156.20, 153.74, 150.58, 148.11, 137.03, 136.42, 131.93, 131.42, 129.57, 129.42, 128.95, 128.91, 128.70, 128.69, 128.40, 128.25, 128.02, 127.97, 126.28, 126.13, 119.23, 118.79, 116.07, 112.98, 112.93, 70.55, 70.51, 64.46, 64.41, 14.44, 14.40.

ESI-HRMS: [M+H$^+$] calculated for $C_{26}H_{23}N_2O_5^+$: 443.1607 Da, found: 443.1599 m/z.

Elemental analysis: calculated for $C_{26}H_{22}N_2O_5$: C=70.58 %, H=5.01 %, N=6.33 %, found: C=70.73 %, H=5.077 %, N=6.397 %.

NMR spectra for **PB-2.10** are in supplementary figure 31.

2-(4-(2,4-Dichlorobenzyloxy)-3-ethoxybenzylidene)-malonamide (PB-2.12)

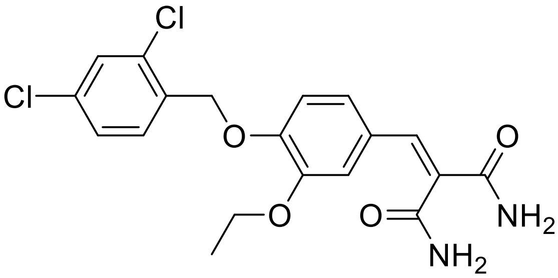

Under nitrogen atmosphere, **PB-2.2** (88.9 mg, 0.273 mmol) and malonamide (30.7 mg, 0.300 mmol) was suspended in toluene (5 ml) in presence of molecular sieves (3 Å). Next, piperidine (27.2 µl, 0.273 mmol) and glacial acetic acid (15.5 µl, 0.273 mmol) were added to reaction mixture and was heated under reflux for 5 h, then cooled down to room temperature. Ethyl acetate (10 ml) was added to the reaction mixture and precipitates were formed, which were filtered and washed with ethyl acetate. Combined organic phases were evaporated and product **PB-2.12** was purified by MPLC (DCM / Methanol, 99:1 to 80:20).

Yield: 14 mg (13 %)

^{1}H NMR (500 MHz, DMSO-d_6) δ [ppm] = 7.82 (s, 1H), 7.70(s, 1H), 7.60 (d, *J* = 8.9 Hz, 1H), 7.50 – 7.47 (m, 2H), 7.29 (s, 1H), 7.23 (s, 2H), 7.13 – 7.04 (m,3H), 5.18 (s, 2H), 4.03 – 3.99 (m, 2H),1.35 – 1.30 (m, 3H).

^{13}C NMR (126 MHz, DMSO-d_6) δ [ppm] = 169.60, 165.80, 148.62, 147.98, 134.06, 133.56, 133.47, 133.40, 131.45, 131.09, 128.90, 127.53, 123.17, 119.41, 114.20, 113.90, 66.99, 63.92, 14.64.

ESI-HRMS: [M+H$^+$] calculated for $C_{19}H_{19}Cl_2N_2O_4{}^+$: 409.0722 Da, found: 409.0718 m/z.

Elemental analysis: calculated for $C_{19}H_{18}Cl_2N_2O_4$: C=55.76 %, H=4.43 %, N=6.84 %, found: C=55.97 %, H=4.699 %, N=6.921 %.

NMR spectra for **PB-2.12** are in supplementary figure 32.

(E/Z)-5-((5-(2,4-Dichlorophenyl)-furan-2-yl)-methylene)-1-(3,5-dimethylphenyl)-pyrimidine-2,4,6(1H,3H,5H)-trione (PB-3)

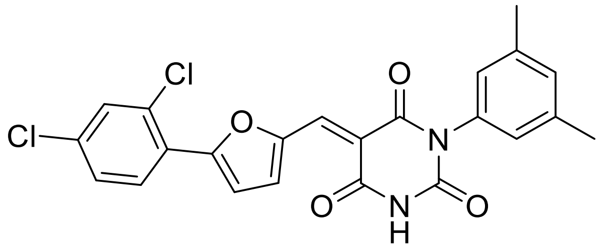

5-(2,4-Dichlorophenyl)-furan-2-carbaldehyde (60.3 mg, 0.25 mmol) and **PB-2.3** (58.1 mg, 0.25 mmol) were suspended in ethanol (10 ml). The suspension was heated under reflux for 4 h and cooled down to room temperature. Yellow crystals were collected by filtration, washed with ethanol and diethylether, and dried in vacuo to furnish product **PB-3**.

Yield: 89 mg (78 %)

Melting point: 291 °C

^{1}H NMR (500 MHz, DMF-d$_7$) δ [ppm] = 11.74 – 11.70 (m, 1H), 8.71 – 8.53 (q, 1H), 8.29 (d, *J* = 24.9Hz, 1H), 8.21 – 8.15 (m, 1H), 7.82 (s, 1H), 7.70 – 7.55 (m, 2H), 7.10 – 7.04 (m, 3H), 2.35, 2.33 (2s, 6H).

^{13}C NMR (126 MHz, DMF-*d*$_7$) δ [ppm] = 163.57, 162.72, 155.70, 150.79, 138.67, 137.30, 136.99, 136.18, 135.83, 135.54, 132.02, 131.04, 130.84, 130.11, 128.61, 127.07, 126.58, 116.43, 114.59, 20.68.

ESI-HRMS: [M+H$^+$] calculated for $C_{23}H_{17}Cl_2N_2O_4^+$: 455.0565 Da, found: 455.0556 m/z.

Elemental analysis: calculated for $C_{23}H_{16}Cl_2N_2O_4$: C=60.68 %, H=3.54 %, N=6.15 %, found: C=60.80 %, H=3.577 %, N=6.173 %.

NMR spectra for **PB-3** are in supplementary figure 33.

5-((5-(2,4-Dichlorophenyl)furan-2-yl)methylene)pyrimidine-2,4,6(1H,3H,5H)-trione (PB-3.2)

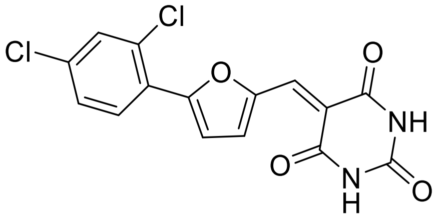

5-(2,4-Dichlorophenyl)-furan-2-carbaldehyde (60.3 mg, 0.25 mmol) and pyrimidine-2,4,6(1H,3H,5H)-trione (38 mg, 0.3 mmol) were suspended in ethanol (40 mol %, 10 µl). The reaction mixture was heated under reflux for 4 h and cooled down to room temperature. Yellow precipitate was collected by filtration, washed with ethanol and diethylether, and dried in vacuum to yield the product **(PB-3.2)**.

Yield: 68 mg (77 %).

Melting point:326 °C

^{1}H NMR (600 MHz, DMSO-d_6) δ [ppm] = 11.38 , 11.30 (2d, *J* = 1.9 Hz, 2H), 8.51 (d, *J* = 3.9 Hz, 1H), 8.11 (d, *J* = 8.6 Hz, 1H), 8.10 (s, 1H), 7.83 (d, *J* = 2.1 Hz, 1H), 7.61 (dd, *J* = 8.6, 2.1 Hz, 1H), 7.56 (d, *J* = 4.0,1H).

^{13}C NMR (151 MHz, DMSO-d_6) δ [ppm] = 163.77, 162.57, 155.26, 150.74, 150.42, 136.43, 135.31, 131.79, 131.12, 130.94, 128.73, 126.42, 116.49, 114.34.

ESI-HRMS: [M+H$^+$] calculated for $C_{15}H_9Cl_2N_2O_4^+$: 350.9934 Da, found: 350.9926 m/z.

Elemental analysis: calculated for $C_{15}H_8Cl_2N_2O_4$: C= 51.31 %, H= 2.30%, N= 7.98 %, found: C=51.54%, H=2.701%, N=7.988%.

NMR spectra for **PB-3.2** are in supplementary figure 34.

Methyl (E/Z)-2-cyano-3-(5-(2,4-dichlorophenyl)furan-2-yl)acrylate (PB-3.3)

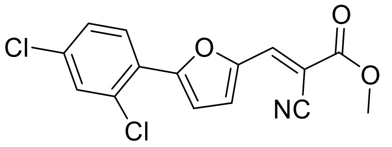

Methyl 2-cyanoacetate (22 µl, 0.25 mmol) was added to a stirred suspension of 5-(2,4-dichlorophenyl) furan-2-carbaldehyde (60.3 mg, 0.25 mmol) in methanol (12 ml). Piperidine (40 mol %, 10 µl) was added. The resulting reaction mixture was stirred overnight at room temperature. The solvent was removed under vacuum and the residue was dissolved in DCM. The organic layer was washed with brine and Saturated $NaHCO_3$ solution, then dried over $MgSO_4$, and dried in vacuum to yield the product **(PB-3.3)** as a yellow solid.

Yield: 78 mg (97 %)

Melting point: 148 °C

^{1}H NMR (500 MHz, $CDCl_3$) δ [ppm] = 8.10 (d, *J* = 8.6 Hz, 1H), 7.98 (s, 1H), 7.49 (d, *J* = 2.1 Hz, 1H), 7.43 (d, *J* = 3.9 Hz, 1H), 7.41 (dd, *J* = 8.6, 2.1 Hz, 1H), 7.30 (d, *J* = 3.8 Hz, 1H), 3.93 (s, 3H).

^{13}C NMR (126 MHz, $CDCl_3$) δ[ppm] = 163.37, 155.21, 147.64, 138.25, 135.69, 131.85, 130.84, 130.07, 128.22, 125.94, 124.75, 115.78, 114.71, 98.31, 53.43.

ESI-HRMS: [M+H$^+$] calculated for $C_{15}H_{10}Cl_2NO_3^+$: 322.0032 Da, found: 322.0034 m/z.

Elemental analysis: calculated for $C_{15}H_9Cl_2NO_3$: C= 55.93 %, H= 2.82%, N= 4.35 %, found: C=55.93%, H=2.902%, N=4.478%.

NMR spectra for **PB-3.3** are in supplementary figure 35.

(E/Z)-3-(5-(2,4-Dichlorophenyl)furan-2-yl)-2-(4-methoxybenzoyl)acrylonitrile (PB-3.4)

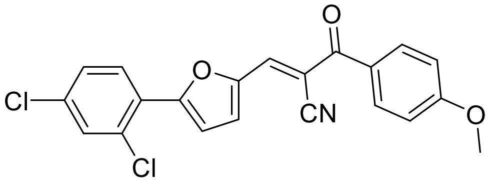

3-(4-Methoxyphenyl)-3-oxopropanenitrile (43.8 mg, 0.25 mmol) was added to a stirred suspension of 5-(2, 4-dichlorophenyl) furan-2-carbaldehyde (60.3 mg, 0.25 mmol) in methanol (12 ml). Piperidine (40 mol %, 10 µl) was added. The resulting reaction mixture was stirred overnight at room temperature. The solvent was removed under vacuum and the residue was dissolved in DCM. The organic layer was washed with brine and Saturated $NaHCO_3$ solution, then dried over $MgSO_4$, and dried in vacuum to yield the product **(PB-3.4)** as a yellow solid.

Yield: 91 mg (91 %)

Melting point:157 °C

1H NMR (500 MHz, $CDCl_3$) δ [ppm] = 8.14 (d, *J* = 8.6 Hz, 1H), 7.98 (d, *J* = 8.9 Hz, 2H), 7.92 (s, 1H), 7.50 (d, *J* = 2.1 Hz, 1H), 7.46 (d, *J* = 3.9 Hz, 1H), 7.40 (dd, *J* = 8.6, 2.1 Hz, 1H), 7.37 – 7.34 (m, 1H), 7.00 (d, *J* = 8.9 Hz, 2H), 3.90 (s, 3H).

13C NMR (126 MHz, $CDCl_3$) δ [ppm] =186.36, 163.99, 154.97, 148.29, 138.57, 135.62, 131.88, 131.85, 130.86, 130.07, 128.82, 128.18, 126.05, 124.64, 118.01, 114.86, 114.02, 105.50, 55.69.

ESI-HRMS: [M+H$^+$] calculated for $C_{21}H_{14}Cl_2NO_3^+$: 398.0345 Da, found: 398.0345 m/z.

Elemental analysis: calculated for $C_{21}H_{13}Cl_2NO_3$: C=63.34%, H=3.29%, N=3.52%, found: C=63.51%, H=3.371%, N=3.551%.

NMR spectra for **PB-3.4** are in supplementary figure 36.

(E/Z)-2-Benzoyl-3-(5-(2,4-dichlorophenyl)furan-2-yl)acrylonitrile (PB-3.5)

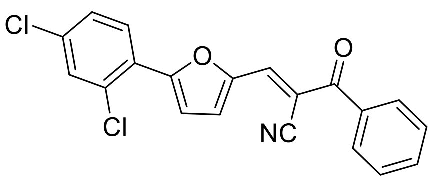

3-oxo-3-phenylpropanenitrile (36.3 mg, 0.25 mmol) was added to a stirred suspension of 5-(2,4-dichlorophenyl) furan-2-carbaldehyde (60.3 mg, 0.25 mmol) in methanol (12 ml). Piperidine (40 mol %, 10 µl) was added. The resulting reaction mixture was stirred overnight at room temperature. The solvent was removed under vacuum and the residue was dissolved in DCM. The organic layer was washed with brine and Saturated $NaHCO_3$ solution, then dried over MgSO4, and dried in vacuum to yield the product **(PB-3.5)** as a yellow solid.

Yield: 90 mg (98 %)

Melting point: 136 °C

^{1}H NMR (500 MHz, $CDCl_3$) δ [ppm] = 8.14 (d, *J* = 8.6 Hz, 1H), 7.93 (s, 1H), 7.92 – 7.90 (m, 2H), 7.66 – 7.60 (m, 1H), 7.53 (d, *J* = 8.0 Hz, 2H), 7.50 (d, *J* = 2.1 Hz, 1H), 7.47 (d, *J* = 3.9 Hz, 1H), 7.40 (m, 2H).

^{13}C NMR (126 MHz, $CDCl_3$) δ[ppm] = 188.35, 155.37, 148.12, 138.91, 136.28, 135.80, 133.36, 131.94, 130.90, 130.13, 129.25, 128.74, 128.21, 125.97, 125.26, 117.58, 114.99, 105.42.

ESI-HRMS: [M+H$^+$] calculated for $C_{20}H_{12}Cl_2NO_2^+$: 368.0240 Da, found: 368.0241 m/z.

Elemental analysis: calculated for $C_{20}H_{11}Cl_2NO_2$: C= 65.24%, H= 3.01%, N= 3.80 %, found: C=65.42%, H=3.121%, N=3.866%.

NMR spectra for **PB-3.5** are in supplementary figure 37.

2-((5-(2,4-Dichlorophenyl)furan-2-yl)methylene)malonamide(PB-3.6)

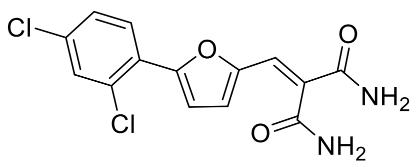

Malonamide (30.6mg, 0.3 mmol) was added to a stirred suspension of 5-(2,4-dichlorophenyl) furan-2-carbaldehyde (60.3 mg, 0.25 mmol) in methanol (12 ml). Piperidine (40 mol %, 10 µl) was added. The resulting reaction mixture was stirred overnight at room temperature. The solvent was evaporated under vacuum. The residue was purified using normal phase flash chromatography with Ethyl acetate/Methanol 9:1 (v/v) mixture to yield the product **(PB-3.6)** as a white solid.

Yield: 77mg (95 %)

Melting point: 276 °C

^{1}H NMR (600 MHz, DMSO-D_6) δ [ppm]= 7.98 (s, 1H), 7.92 (d, J = 8.6 Hz, 1H), 7.75 (d, J = 2.2 Hz, 1H), 7.66 (s, 1H), 7.50 (dd, J = 8.7, 2.2 Hz, 1H), 7.35 (s, 1H), 7.30 (d, J = 3.7 Hz, 1H), 7.20 (s, 1H), 7.17 (s, 1H), 7.01 (d, J = 3.7 Hz, 1H).

^{13}C NMR (151 MHz, DMSO-D_6) δ[ppm] = 169.35, 165.75, 150.55, 150.07, 133.54, 130.99, 130.79, 130.54, 129.79, 128.55, 127.25, 121.51, 118.08, 114.37.

ESI-HRMS: [M+H$^+$] calculated for $C_{14}H_{11}Cl_2N_2O_3^+$: 325.0141Da, found: 325.0147 m/z.

Elemental analysis: calculated for $C_{14}H_{10}Cl_2N_2O_3$: C= 51.72%, H= 3.10%, N= 8.62 %, found: C=51.81%, H=3.460%, N=8.625%.

NMR spectra for **PB-3.6** are in supplementary figure 38.

Methyl (E/Z)-4-(2-cyano-3-(5-(2,4-dichlorophenyl)furan-2-yl)acryloyl)benzoate (PB-3.7)

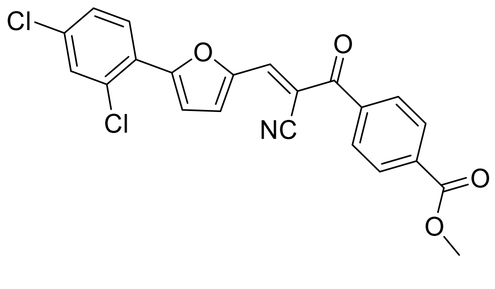

Methyl 4-(2-cyanoacetyl) benzoate (50.8 mg, 0.25 mmol) was added to a stirred suspension of 5-(2,4-dichlorophenyl) furan-2-carbaldehyde (60.3 mg, 0.25 mmol) in methanol (12 ml). Piperidine (40 mol %, 10 µl) was added. The resulting reaction mixture was stirred overnight at room temperature. The solvent was removed under vacuum and the residue was dissolved in DCM. The organic layer was washed with brine and Saturated $NaHCO_3$ solution, then dried over MgSO4, and dried in vacuum to yield the product **(PB-3.7)** as a yellow solid.

Yield: 96 mg (90 %)

Melting point: 188 °C

^{1}H NMR (600 MHz, $CDCl_3$) δ[ppm] = 8.18 (d, *J* = 8.4 Hz, 2H), 8.14 (d, *J* = 8.6 Hz, 1H), 7.95 (d, *J* = 8.4 Hz, 2H), 7.94 (s, 1H), 7.52 (d, *J* = 2.1 Hz, 1H), 7.49 (d, *J* = 3.9 Hz, 1H), 7.44 – 7.41 (m, 2H), 3.97 (s, 3H).

^{13}C NMR (151 MHz, $CDCl_3$) δ[ppm] = 187.90, 166.15, 155.85, 148.02, 139.88, 139.14, 136.01, 134.03, 132.06, 130.95, 130.17, 129.89, 129.66, 129.04, 128.25, 125.83, 117.35, 115.18, 104.79, 52.66.

ESI-HRMS: [M+H$^+$] calculated for $C_{22}H_{14}Cl_2NO_4^+$: 426.0294 Da, found: 426.0293 m/z.

Elemental analysis: calculated for $C_{22}H_{13}Cl_2NO_4$: C= 61.99 %, H= 3.07%, N= 3.29 %, found: C=62.13%, H=3.126%, N=3.343%.

NMR spectra for **PB-3.7** are in supplementary figure 39.

(E/Z)-5-((5-(2,4-Difluorophenyl)furan-2-yl)methylene)-1-(3,5-dimethylphenyl)pyrimidine-2,4,6(1H,3H,5H)-trione (PB-3.8)

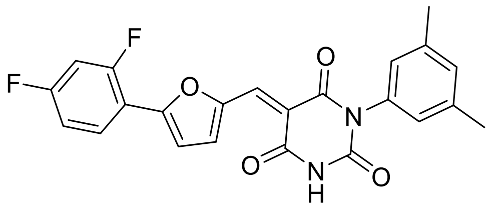

5-(2,4-Difluorophenyl)furan-2-carbaldehyde (52 mg, 0.25 mmol) and 1-(3,5-dimethylphenyl)pyrimidine-2,4,6(1H,3H,5H)-trione (70 mg, 0.3 mmol) were suspended in ethanol (10 ml). The reaction mixture was heated overnight under reflux, and cooled down to room temperature. Yellow precipitate was collected by filtration, washed with ethanol and diethylether, and then was purified using normal phase flash chromatography with a Dichloromethane/ Methanol (0.8%) mixture to yield the product **(PB-3.8)**.

Yield: 77 mg (73%)

Melting point: 274 °C

^{1}H NMR (600 MHz, DMSO-d_6) δ [ppm] = 11.66 (d, J = 44 Hz, 1H), 8.61 – 8.45 (m, 1H), 8.21 – 8.07 (m, 2H), 7.54 – 7.46 (m, 1H), 7.31 – 7.16 (m, 2H), 7.07 – 7.05 (m, 1H), 6.94 – 6.91 (m, 2H), 2.36 – 2.22 (m, 6H).

^{13}C NMR (151 MHz, DMSO-d_6) δ[ppm]= 164.37 , 163.52, 162.76 , 162.06, 161.79, 159.03 , 154.01 , 150.74 , 150.26 , 138.52 , 137.01 , 135.88, 135.52, 130.27, 129.56 , 127.08 , 115.15 , 113.60, 105.88 , 21.24.

^{19}F NMR (376 MHz, DMSO-d_6) δ [ppm] = -106.08, -108.14.

ESI-HRMS: [M+H$^+$] calculated for $C_{23}H_{17}F_2N_2O_4^+$: 423.1151 Da, found: 423.1163 m/z.

Elemental analysis: calculated for $C_{23}H_{16}F_2N_2O_4$: C= 65.40%, H= 3.82%, N= 6.63 %, found: C=65.58%, H=3.968%, N=6.495%.

NMR spectra for **PB-3.8** are in supplementary figure 40.

(E/Z)-1-(3,5-Dimethylphenyl)-5-((5-(4-methoxyphenyl)furan-2-yl)methylene)pyrimidine-2,4,6(1H,3H,5H)-trione(PB-3.9)

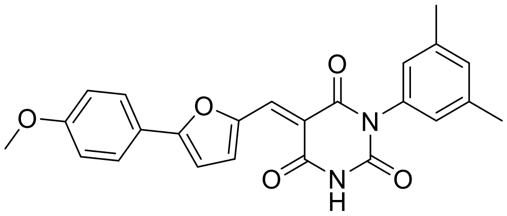

5-(4-Methoxyphenyl)furan-2-carbaldehyde (51 mg, 0.25 mmol) and 1-(3,5-dimethylphenyl)pyrimidine-2,4,6(1H,3H,5H)-trione (70 mg, 0.3 mmol) were suspended in ethanol (10 ml). The reaction mixture was heated overnight under reflux, and cooled down to room temperature. Red precipitate was collected by filtration, washed with ethanol and diethylether, and then was purified using normal phase flash chromatography with a Dichloromethane/ Methanol (1%) mixture to yield the product **(PB-3.9)**.

Yield: 78 mg (75%)

Melting point:284 °C

^{1}H NMR (600 MHz, DMSO-d_6) δ [ppm] = δ 11.58 (s, 1H), 8.66 – 8.48 (m, 1H), 8.15 (d, *J* = 50.0 Hz, 1H), 7.91 (dd, *J* = 8.8, 3.6 Hz, 2H), 7.37 – 7.29 (m, 1H), 7.09 – 7.07 (m, 2H), 7.05 (d, *J* = 5.9 Hz, 1H), 6.92 (d, *J* = 11.5 Hz, 2H), 3.83 (s, 3H), 2.30 (d, *J* = 2.5 Hz, 6H).

^{13}C NMR (151 MHz, DMSO-d_6) δ[ppm]= 163.40, 162.78, 161.85, 161.59, 161.44 , 161.26, 150.10 , 138.13 , 136.56 , 135.46 , 130.89 , 129.83, 127.52, 126.78 , 121.14 , 115.01, 110.84, 55.64, 20.87.

ESI-HRMS: [M+H$^+$] calculated for $C_{24}H_{21}N_2O_5^+$: 417.1445 Da, found: 417.1455 m/z.

Elemental analysis: calculated for $C_{24}H_{20}N_2O_5$: C= 69.22%, H= 4.84%, N= 6.73 %, found: C=69.23%, H= 4.856%, N=6.740%.

NMR spectra for **PB-3.9** are in supplementary figure 41.

Methyl (E/Z)-2-cyano-3-(5-(2,4-difluorophenyl)furan-2-yl)acrylate (PB-3.10)

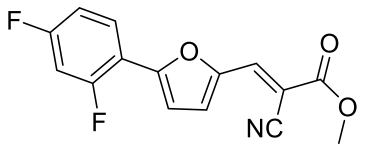

Methyl 2-cyanoacetate (24 µl, 0.275 mmol) was added to a stirred suspension of 5-(2,4-difluorophenyl) furan-2-carbaldehyde (52 mg, 0.25 mmol) in methanol (12 ml). Piperidine (40 mol %, 10 µl) was added. The resulting reaction mixture was stirred overnight at room temperature. The solvent was removed under vacuum and the residue was dissolved in DCM. The organic layer was washed with brine, then dried over Na_2SO_4. The organic solvent was evaporated and the residue was purified using normal phase flash chromatography with a hexane/ethyl acetate 95:5 (v/v) mixture to yield the product **(PB-3.10)** as a yellow solid.

Yield: 62 mg (85 %)

Melting point:186 °C

^{1}H NMR (600 MHz, $CDCl_3$) δ [ppm] = 8.12 – 8.03 (m, 1H), 7.94 (s, 1H), 7.27 (d, *J* = 3.2 Hz, 1H), 7.05 – 7.01 (m, 1H), 7.00 (t, *J* = 3.7 Hz, 1H), 6.93 – 6.89 (m, 1H), 3.92 (s, 3H).

^{13}C NMR (151 MHz, $CDCl_3$) δ[ppm]= 164.48-162.52(m) ,163.48,160.02, 153.55, 147.45, 138.17, 128.98, 125.37, 115.88, 114.00, 113.15, 112.78, 104.76, 97.57, 53.35.

^{19}F NMR (376 MHz, $CDCl_3$) δ [ppm] = -108.01, -106.04

ESI-HRMS: [M+H$^+$] calculated for $C_{15}H_{10}F_2NO_3^+$: 290.0623 Da, found: 290.0628 m/z.

Elemental analysis: calculated for $C_{15}H_9F_2NO_3$: C= 62.29 %, H= 3.14%, N= 4.84 %, found: C=62.25%, H=3.562%, N=4.870%.

NMR spectra for **PB-3.10** are in supplementary figure 42.

Methyl (E/Z)-2-cyano-3-(5-(4-methoxyphenyl)furan-2-yl)acrylate (PB-3.11)

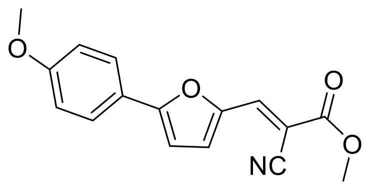

Methyl 2-cyanoacetate (24 µl, 0.275 mmol) was added to a stirred suspension of 5-(4-methoxyphenyl) furan-2-carbaldehyde (51 mg, 0.25 mmol) in methanol (12 ml). Piperidine (40 mol %, 10 µl) was added. The resulting reaction mixture was stirred overnight at room temperature. The solvent was evaporated under vacuum and the residue was dissolved in DCM. The organic layer was washed with brine, then dried over Na_2SO_4, The organic solvent was evaporated and the residue was purified using normal phase flash chromatography with a hexane/ethyl acetate 92:8 (v/v) mixture to yield the product **(PB-3.11)** as a yellow solid.

Yield: 57 mg (81 %)

Melting point:159 °C

^{1}H NMR (600 MHz, $CDCl_3$) δ [ppm] = 7.91 (s, 1H), 7.84 – 7.65 (m, 2H), 7.48 – 7.27 (m, 1H), 7.00 – 6.93 (m, 2H), 6.77 (dd, *J* = 9.9, 3.5 Hz, 1H), 3.89 (d, *J* = 12.1 Hz, 3H), 3.85 (d, *J* = 3.5 Hz, 3H).

^{13}C NMR (151 MHz, $CDCl_3$) δ[ppm] = 164.00, 161.21, 160.46, 147.91, 140.03, 128.41, 127.15, 121.64, 117.36, 114.70, 108.44, 95.58, 55.51, 52.96.

ESI-HRMS: [M+H$^+$] calculated for $C_{16}H_{14}NO_4^+$: 284.0917 Da, found: 284.0922 m/z.

Elemental analysis: calculated for $C_{16}H_{13}NO_4$: C= 67.84 %, H= 4.63%, N= 4.94 %, found: C=67.88%, H=4.692%, N=4.970%.

NMR spectra for **PB-3.11** are in supplementary figure 43.

1-(2-Hydroxyethyl)-pyrimidine-2,4,6(1H,3H,5H)-trione (30)

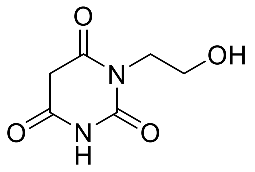

Diethyl malonate (3.661 ml, 24mmol) and 1-(2-hydroxyethyl) urea (833 mg, 8 mmol) were added to a stirred solution of sodium (460 mg, 20 mmol) in ethanol (40 ml). The reaction mixture was refluxed overnight. The solvent was evaporated, and the residue was purified using normal phase flash chromatography with a Dichloromethane/ Methanol (9%)/ Formic acid (0.1%) mixture to yield the product **(30)** as a yellow oil.

Yield: 812 mg (59 %).

^{1}H NMR (600 MHz, DMSO-d_6)δ [ppm] = 11.30 – 11.28 (m, 1H), 4.71 (s, 1H), 3.75 (t, *J* = 6.5 Hz, 2H), 3.60 (s, 2H), 3.45 (t, *J* = 6.5 Hz, 2H).

^{13}C NMR (151 MHz, CD_3OD) δ [ppm] = 167.33, 166.90, 151.93, 58.62, 42.21, 38.53.

ESI-HRMS: [M+H$^+$] calculated for $C_6H_9N_2O_4^+$: 173.0557 Da, found: 173.0562 m/z.

Elemental analysis: calculated for $C_6H_8N_2O_4$: C=41.86 %, H=4.68%, N=16.27 % found: : C=41.91 %, H=4.737%, N=16.60 %.

NMR spectra for **30** are in supplementary figure 44.

(E)-5-((5-(2,4-Dichlorophenyl)furan-2-yl)methylene)-1-(2-hydroxyethyl)pyrimidine-2,4,6(1H,3H,5H)-trione (PB-3.12)

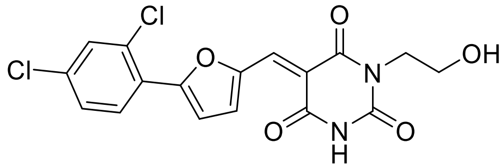

5-(2,4-Dichlorophenyl)-furan-2-carbaldehyde (83 mg, 0.344 mmol) and 1-(2-hydroxyethyl) pyrimidine-2,4,6(1H,3H,5H)-trione (71 mg, 0.413 mmol) were suspended in 10 ml of ethanol/ methanol 1:1 (v/v). Piperidine (29 mol %, 10 µl) was added. The reaction mixture was heated overnight under reflux, and cooled down to room temperature. yellow precipitate was collected by filtration, washed with ethanol and diethylether, and dried in vacuum to give the product **(PB-3.12)**.

Yield: 88 mg (65%)

Melting point: 253 °C

^{1}H NMR (500 MHz, DMSO-d_6) δ [ppm] = 11.56 (d, *J* = 37 Hz, 1H), 8.51 (dd, *J* = 8.1, 4.0 Hz, 1H), 8.10 (d, *J* = 4.4 Hz, 1H), 8.07 (d, *J* = 8.6 Hz, 1H), 7.79 (d, *J* = 2.1 Hz, 1H), 7.58 (dt, *J* = 8.6, 2.4 Hz, 1H), 7.54 (dd, *J* = 4.1, 0.8 Hz, 1H), 4.79 (q, *J* = 5.8 Hz, 1H), 3.88 (td, *J* = 6.4, 3.9 Hz, 2H), 3.53 (p, *J* = 6.3 Hz, 2H).

^{13}C NMR (126 MHz, DMSO-d_6) δ[ppm]= 163.26, 162.50, 161.84, 161.44, 155.28 , 150.82 , 150.30, 136.83 , 135.25, 131.70, 130.91 , 128.68 , 126.21 , 116.46, 114.09 , 58.16, 43.02 .

ESI-HRMS: [M+H$^+$] calculated for $C_{17}H_{13}Cl_2N_2O_5^+$: 395.0196 Da, found: 395.0205 m/z.

Elemental analysis: calculated for $C_{17}H_{12}Cl_2N_2O_5$: C= 51.67%, H= 3.06%, N= 7.09 %, found: C=51.86%, H= 3.159%, N=7.167%.

NMR spectra for **PB-3.12** are in supplementary figure 45.

1-(Pyridin-2-yl)pyrimidine-2,4,6(1H,3H,5H)-trione (33)

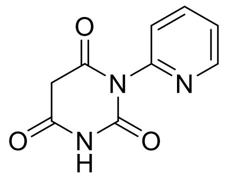

Diethyl malonate (3.661 ml, 24mmol) and 1-(pyridin-2-yl) urea (1.1 g, 8 mmol) were added to a stirred solution of sodium (460 mg, 20 mmol) in ethanol (35 ml). The reaction mixture was refluxed overnight. The solvent was evaporated, and water (25 ml) was added to residue. Further, 2 M HCl was added dropwise until pH 1-2 was reached and the solution was stirred for 0.5 h. The white precipitate was filtered off, washed with water, and then was purified using normal phase flash chromatography with a Dichloromethane/ Methanol (14%)/ Formic acid (0.1%) mixture to yield the product **(33)** as a white solid.

Yield: 780 mg (48 %)

Melting point: 263 °C

^{1}H NMR (700 MHz, DMSO-d_6) δ [ppm] = 11.53 (s, 1H), 8.57 (dd, *J* = 5.0, 1.9 Hz, 1H), 7.97 (td, *J* = 7.7, 2.0 Hz, 1H), 7.48 (dd, *J* = 7.5, 4.8 Hz, 1H), 7.38 (d, *J* = 7.9 Hz, 1H), 3.82 (s, 2H).

^{13}C NMR (176 MHz, DMSO-d_6) δ [ppm] = 167.03, 163.51, 151.70, 149.69, 149.08, 139.16, 124.78, 124.71.

ESI-HRMS: [M+H$^+$] calculated for $C_9H_8N_3O_3^+$: 206.0560 Da, found: 206.0579 m/z.

Elemental analysis: calculated for $C_9H_7N_3O_3$: C=52.69 %, H=3.44%, N=20.48 % found: C=52.74 %, H=3.522%, N=20.55 %.

NMR spectra for **33** are in supplementary figure 46.

(E/Z)-5-((5-(2,4-Dichlorophenyl)furan-2-yl)methylene)-1-(pyridin-2-yl)pyrimidine-2,4,6(1H,3H,5H)-trione (PB-3.13)

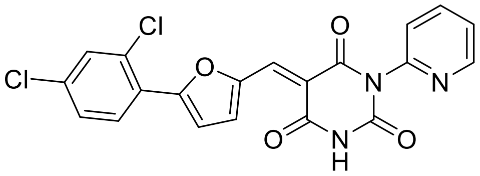

5-(2,4-Dichlorophenyl)-furan-2-carbaldehyde (121 mg, 0.5 mmol) and 1-(pyridin-2-yl) pyrimidine-2,4,6(1H,3H,5H)-trione (123 mg, 0.6 mmol) were suspended in ethanol (7 ml). Piperidine (20 mol %, 10 µl) was added. The reaction mixture was heated overnight under reflux, and cooled down to room temperature. yellow precipitate was collected by filtration, washed with ethanol and diethylether, and dried in vacuum to yield the product **(PB-3.13)**.

Yield: 116 mg (54%)

Melting point:262 °C

^{1}H NMR (700 MHz, DMSO-d_6) δ [ppm] = ^{1}H NMR (700 MHz, DMSO) δ 11.80 (d, *J* = 52 Hz, 1H),8.63 – 8.59 (m, 1H), 8.59 – 8.40 (m, 1H), 8.23 – 8.13 (m, 1H), 8.08 (dd, *J* = 8.6, 4.8 Hz, 1H), 8.04 – 7.98 (m, 1H), 7.79 (dd, *J* = 6.0, 2.2 Hz, 1H), 7.61 – 7.56(m, 1H), 7.55 – 7.48 (m, 2H).

^{13}C NMR (176 MHz, DMSO-d_6) δ[ppm]= 163.24, 162.67, 161.77, 161.59, 155.70 , 150.31 , 149.75 , 149.31, 149.09, 139.26, 137.43 , 135.38, 131.80, 130.96 , 129.30 , 128.63, 126.16 , 124.93 , 116.60 , 113.79 .

ESI-HRMS: [M+H$^+$] calculated for $C_{20}H_{12}Cl_2N_3O_4^+$: 428.0199 Da, found: 428.0201 m/z.

Elemental analysis: calculated for $C_{20}H_{11}Cl_2N_3O_4$: C= 56.10%, H= 2.59%, N= 9.81 %, found: C=56.25 %, H= 2.602 %, N=9.836 %.

NMR spectra for **PB-3.13** are in supplementary figure 47.

Supplementary Note 2: Protein MS Reports

Supplementary Note 2a (report)

**Data File**	PLY_WT.d	**Sample Name**	PLY_WT
**Sample Type**	Sample	**Position**	P1-A1
**Instrument Name**	EMMA	**User Name**	
**Acq Method**	EV68 denat.m	**Acquired Time**	31/03/2021 08:13:53 (UTC+02:00)
**IRM Calibration Status**	Success	**DA Method**	BioConfirmIntactProtein-Default.m
**Comment**			

**Sample Group**		**Info.**	
**Stream Name**	LC 1	**Acquisition Time (Local)**	30/03/2021 23:13:53 (UTC-08:00)
**Acquisition SW Version**	6200 series TOF/6500 series Q-TOF 10.1 (48.0)	**QTOF Driver Version**	10.01.00
**QTOF Firmware Version**	14.808	**Tune Mass Range Max.**	3200

Biomolecules

RT	Mass	Height	Area	Algorithm
10.899	55583.95447	3130	12799994	Maximum Entropy …
10.949	55536.69865	3817	17341861	Maximum Entropy …
10.949	55610.67457	2904	18442509	Maximum Entropy …

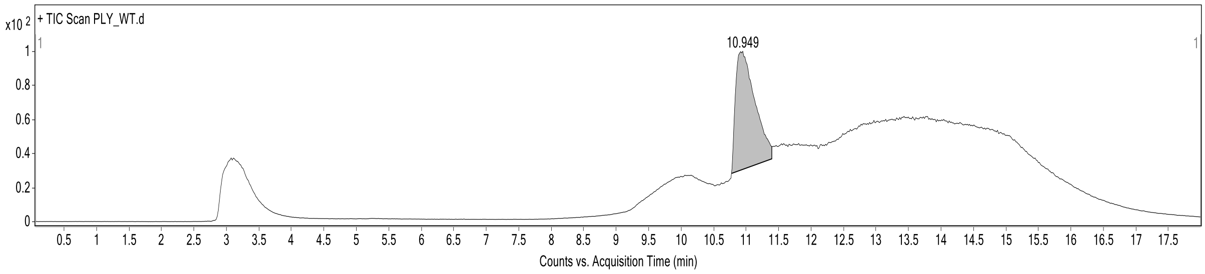

MS Spectrum

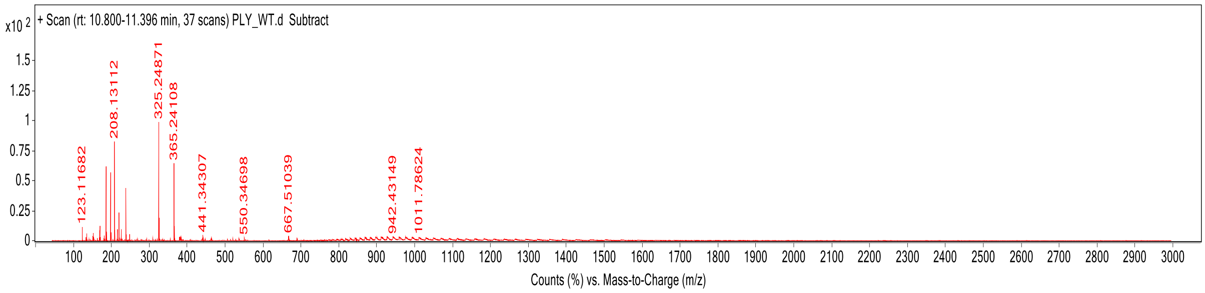

Source Biomolecule Spectrum Peak List

z	Neutral Mass	Abund
	170.15416	10950.21
1	186.14914	55574.88
1	198.14924	49228.53
1	208.13112	76381.49
1	220.13104	20237.3
1	238.14169	37450.48
1	325.24871	85325.78
1	326.25199	16229.61
1	365.24108	55991.7
1	366.2441	10946.38

Deconvoluted Spectrum

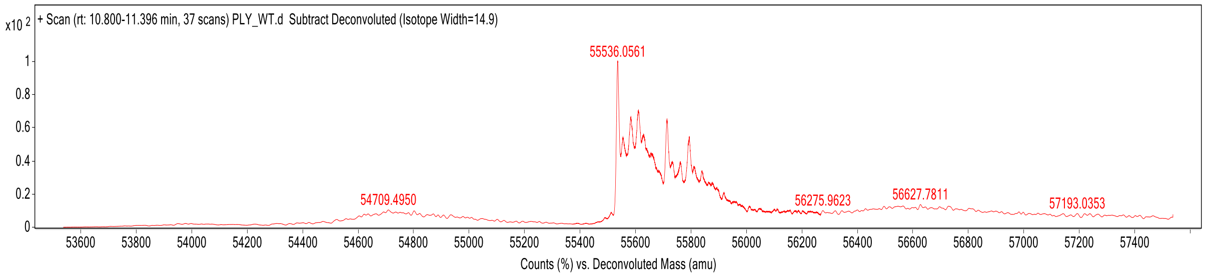

Source Biomolecule Spectrum Peak List

Neutral Mass	Abund
55536.0561	5261.74
55555.5159	2834.93
55583.7507	3481.21
55610.7063	3678.61
55628.9567	2921.78
55657.6473	2301.26
55713.9071	3431.77
55732.9605	2053.89
55761.8844	2053.21
55793.4374	2789.84

Overlaid Deconvolution Plot

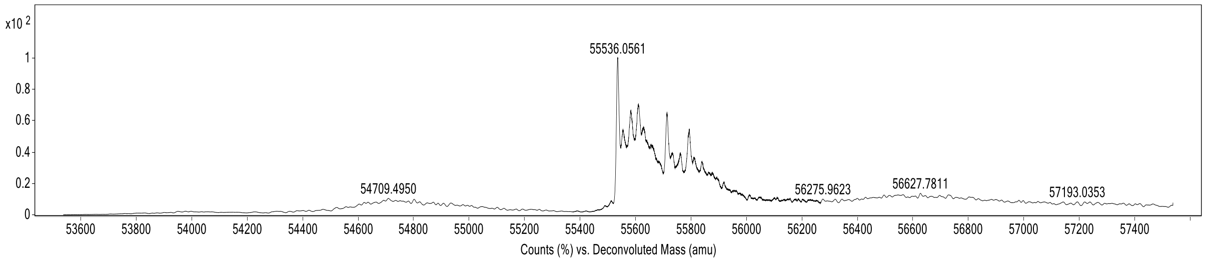

Supplementary Note 2b (report)

**Data File**	PLY_WT+PB3.d	**Sample Name**	PLY_WT+PB3
**Sample Type**	Sample	**Position**	P1-A3
**Instrument Name**	EMMA	**User Name**	
**Acq Method**	EV68 denat.m	**Acquired Time**	31/03/2021 08:51:59 (UTC+02:00)
**IRM Calibration Status**	Success	**DA Method**	BioConfirmIntactProtein-Default.m
**Comment**			

**Sample Group**		**Info.**	
**Stream Name**	LC 1	**Acquisition Time (Local)**	30/03/2021 23:51:59 (UTC-08:00)
**Acquisition SW Version**	6200 series TOF/6500 series Q-TOF 10.1 (48.0)	**QTOF Driver Version**	10.01.00
**QTOF Firmware Version**	14.808	**Tune Mass Range Max.**	3200

Biomolecules

RT	Mass	Height	Area	Algorithm
10.908	55761.14234	1865	4555617	Maximum Entropy …
10.991	55585.93689	2377	2987649	Maximum Entropy …

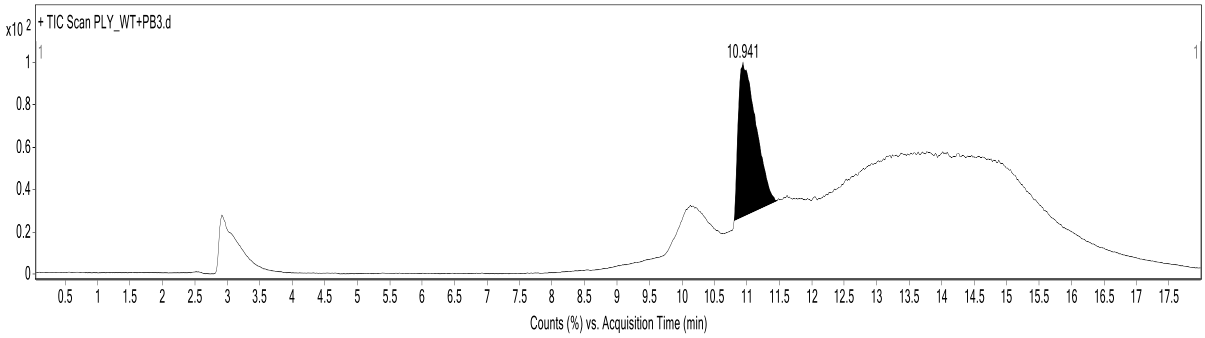

MS Spectrum

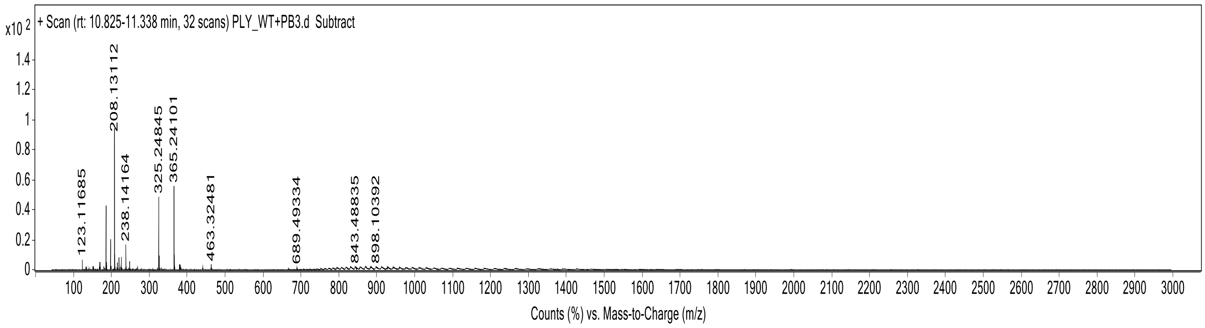

Source Biomolecule Spectrum Peak List

z	Neutral Mass	Abund
1	186.14911	38036.08
1	198.1492	17326.29
1	208.13112	83432.06
1	209.13426	9295.09
	226.14193	7480.6
1	238.14164	14087.49
1	325.24845	41256.98
1	326.25187	7842.17
1	365.24101	47114.52
1	366.2435	8904.62

Deconvoluted Spectrum

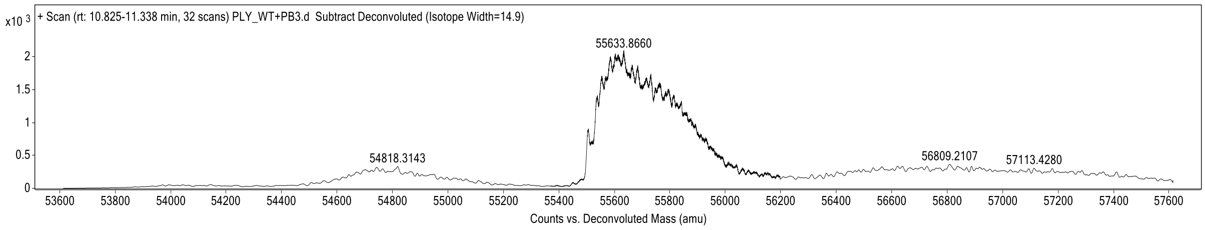

Source Biomolecule Spectrum Peak List

Neutral Mass	Abund
55554.8313	1666.53
55569.1068	1676.29
55585.7049	1972.13
55603.3546	2006.74
55613.6741	1969.53
55633.866	2074.06
55663.9834	1845.48
55683.5572	1852.08
55715.341	1660.34
55731.1141	1713.97

Overlaid Deconvolution Plot

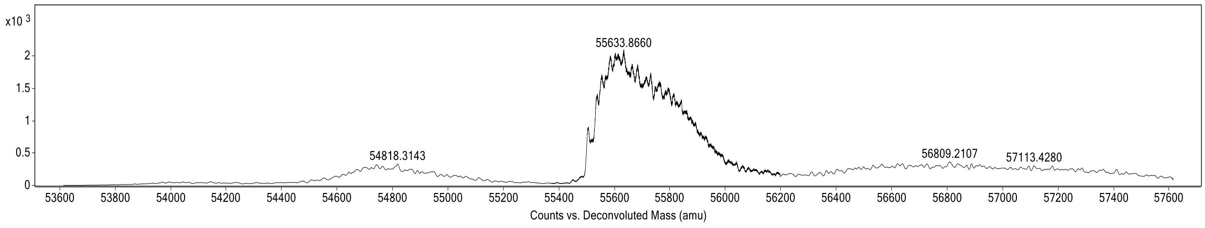

Supplementary Note 2c (report)

**Data File**	PLYPB3.d	**Sample Name**	PLY PB3
**Sample Type**	Sample	**Position**	p1c2
**Instrument Name**	EMMA	**User Name**	
**Acq Method**	EV68 denat.m	**Acquired Time**	15/04/2021 08:43:41 (UTC+02:00)
**IRM Calibration Status**	Success	**DA Method**	BioConfirmIntactProtein-Default.m
**Comment**	Jupiter säule		

**Method part to run:**	Acquisition Only	**Sample Group**	
**Info.**		**Stream Name**	LC 1
**Acquisition Time (Local)**	14/04/2021 23:43:41 (UTC-08:00)	**Acquisition SW Version**	6200 series TOF/6500 series Q-TOF 10.1 (48.0)
**QTOF Driver Version**	10.01.00	**QTOF Firmware Version**	14.808
**Tune Mass Range Max.**	1700		

No Biomolecules found

Biomolecules

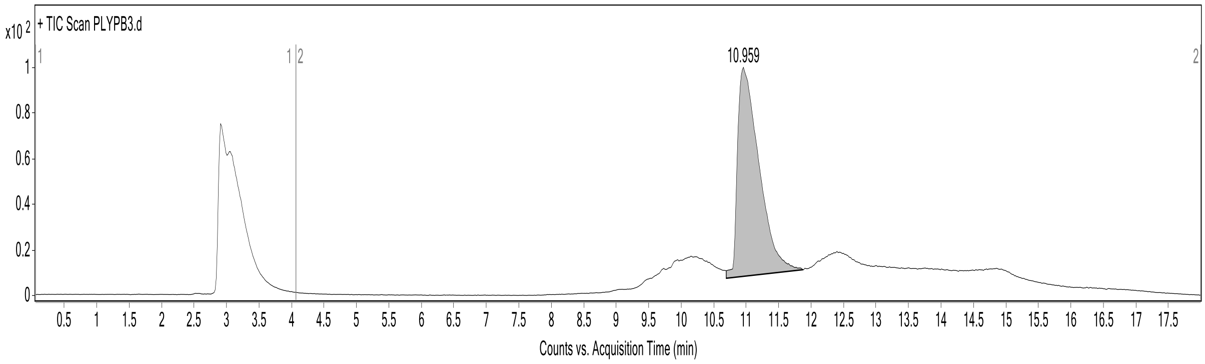

MS Spectrum

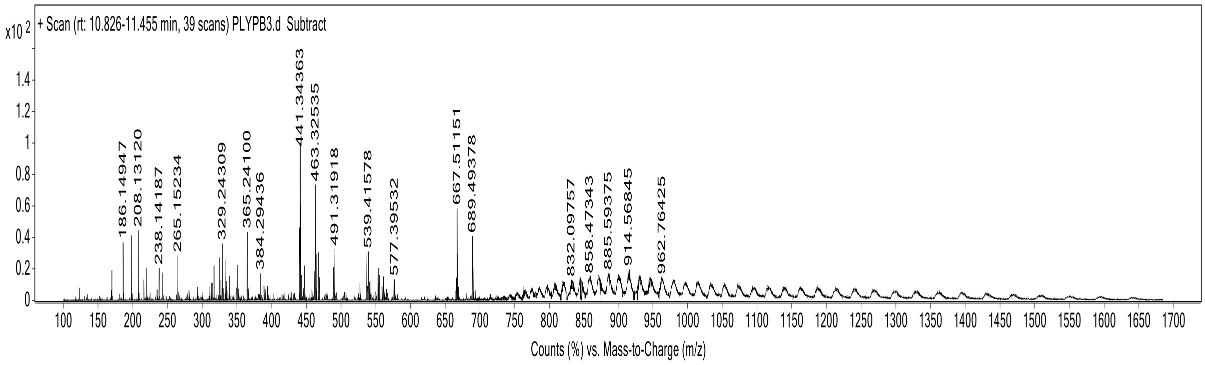

Source Biomolecule Spectrum Peak List

z	Neutral Mass	Abund
	186.14947	2427.17
	198.14905	2774.41
	208.1312	3009.18
	365.241	2845.34
	440.31178	3001.59
	441.34363	6478.38
1	442.3299	4087.06
1	463.32535	4860.82
1	667.51151	3803.02
1	689.49378	2681.92

Deconvoluted Spectrum

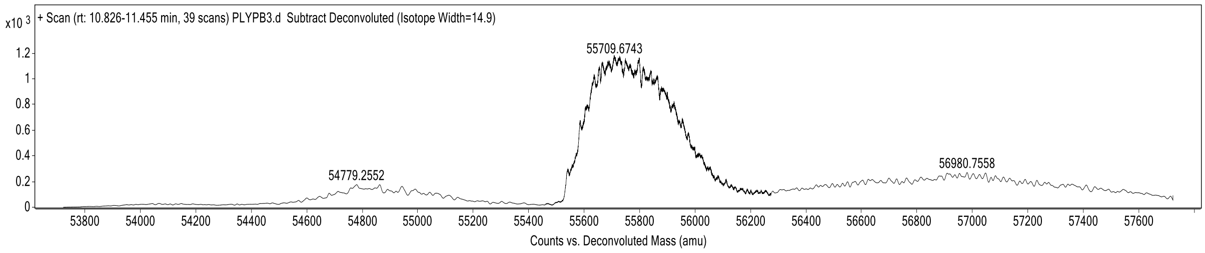

Source Biomolecule Spectrum Peak List

Neutral Mass	Abund
55654.356	1077.62
55666.2495	1114.28
55688.8657	1118.87
55709.6743	1163.3
55727.1298	1150.67
55750.0033	1135.13
55765.6638	1096.87
55783.8044	1059.87
55797.3043	1144.65
55814.0135	1077.94

Overlaid Deconvolution Plot

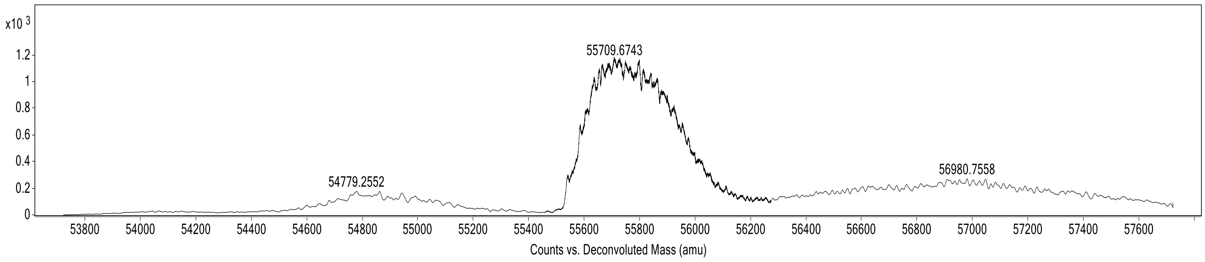

Supplementary Note 2d (report)

**Data File**	PLYC428A.d	**Sample Name**	PLYC428A
**Sample Type**	Sample	**Position**	P1-A2
**Instrument Name**	EMMA	**User Name**	
**Acq Method**	EV68 denat.m	**Acquired Time**	31/03/2021 08:33:01 (UTC+02:00)
**IRM Calibration Status**	Success	**DA Method**	BioConfirmIntactProtein-Default.m
**Comment**			

**Sample Group**		**Info.**	
**Stream Name**	LC 1	**Acquisition Time (Local)**	30/03/2021 23:33:01 (UTC-08:00)
**Acquisition SW Version**	6200 series TOF/6500 series Q-TOF 10.1 (48.0)	**QTOF Driver Version**	10.01.00
**QTOF Firmware Version**	14.808	**Tune Mass Range Max.**	3200

Biomolecules

RT	Mass	Height	Area	Algorithm
10.842	55682.36472	12915	46210113	Maximum Entropy …
10.859	55761.23524	6125	10671290	Maximum Entropy …
10.859	55728.06615	4722	38283304	Maximum Entropy …
10.875	55553.62564	15368	75383905	Maximum Entropy …
10.925	55504.35636	27914	55122213	Maximum Entropy …

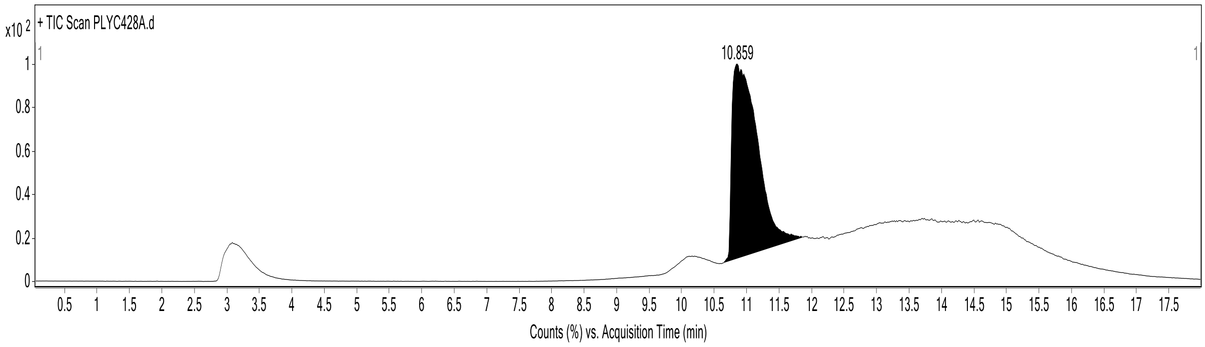

MS Spectrum

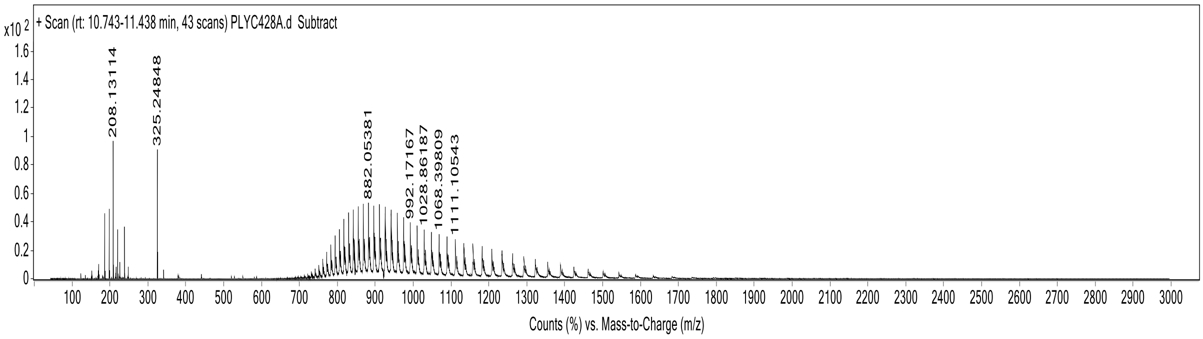

Source Biomolecule Spectrum Peak List

z	Neutral Mass	Abund
	186.14921	13470.2
	198.14908	13474.35
	208.13114	26644.17
	325.24848	25049.6
2	854.92943	13545.78
	868.27304	14008.28
1	882.05381	14226.09
	896.26431	13663.24
1	910.92392	13942.38
1	926.06269	13469.56

Deconvoluted Spectrum

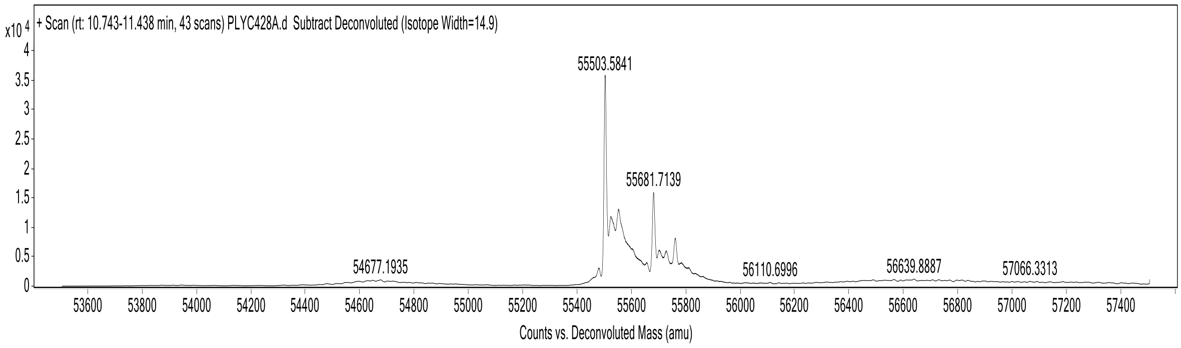

Source Biomolecule Spectrum Peak List

Neutral Mass	Abund
55503.5841	35574.75
55525.9219	11733.25
55552.9434	13013.53
55656.4544	3984.16
55681.7139	15904.28
55703.0698	6091.48
55728.2166	5953.94
55761.3564	8109.04
55784.1955	3942.85
55810.7223	3062.53

Overlaid Deconvolution Plot

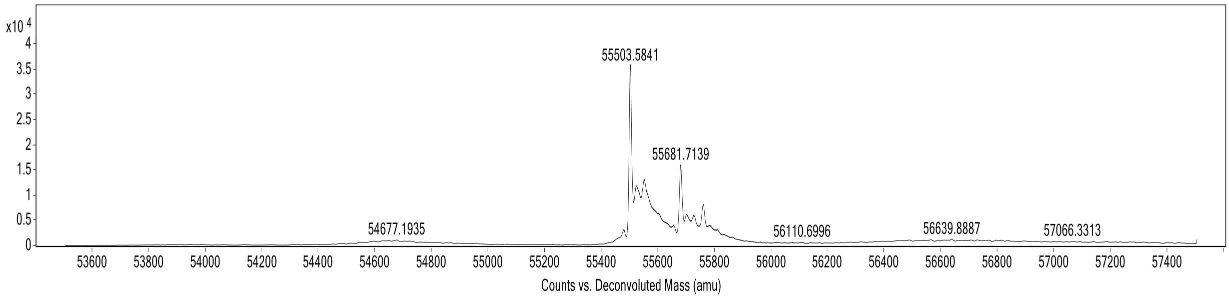

Supplementary Note 2e (report)

**Data File**	PLYC428A+PB3.d	**Sample Name**	PLYC428A+PB3
**Sample Type**	Sample	**Position**	P1-A4
**Instrument Name**	EMMA	**User Name**	
**Acq Method**	EV68 denat.m	**Acquired Time**	31/03/2021 09:10:57 (UTC+02:00)
**IRM Calibration Status**	Success	**DA Method**	BioConfirmIntactProtein-Default.m
**Comment**			

**Sample Group**		**Info.**	
**Stream Name**	LC 1	**Acquisition Time (Local)**	31/03/2021 00:10:57 (UTC-08:00)
**Acquisition SW Version**	6200 series TOF/6500 series Q-TOF 10.1 (48.0)	**QTOF Driver Version**	10.01.00
**QTOF Firmware Version**	14.808	**Tune Mass Range Max.**	3200

Biomolecules

RT	Mass	Height	Area	Algorithm
10.909	55682.10597	8618	42041534	Maximum Entropy …
10.926	55504.67865	20467	44538032	Maximum Entropy …
10.942	55761.59097	4958	30213424	Maximum Entropy …

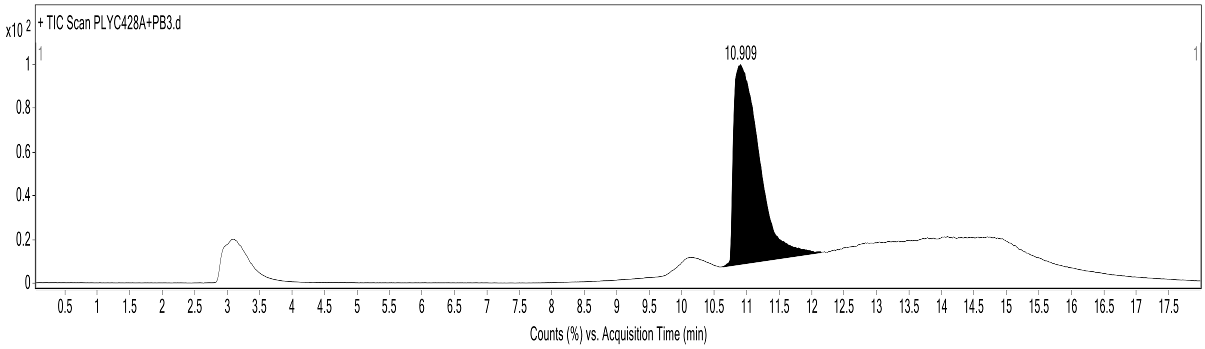

MS Spectrum

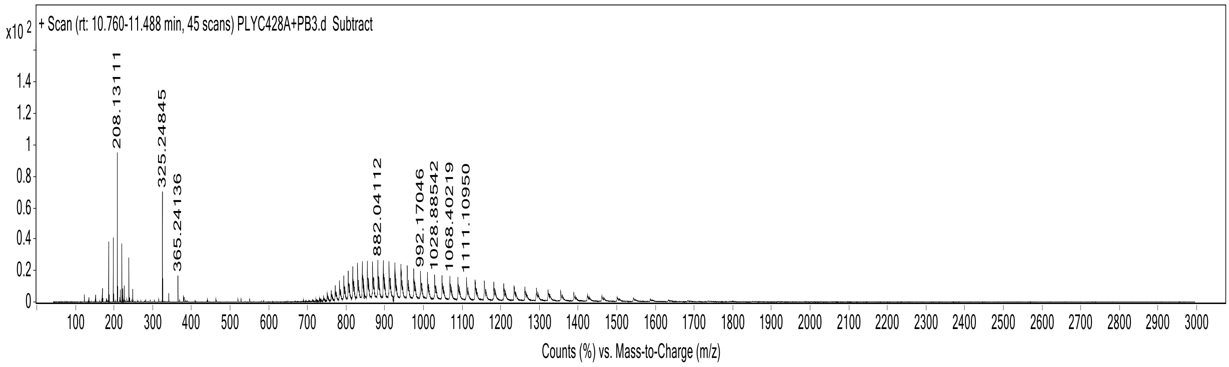

Source Biomolecule Spectrum Peak List

z	Neutral Mass	Abund
	186.14912	15710.43
	198.14907	15924.03
	208.13111	38010.53
	220.13094	14366.88
	238.14149	10773.61
1	325.24845	27307.35
2	854.9411	9945.25
1	882.04112	10151.55
	896.28266	10066.09
1	910.91993	9841.94

Deconvoluted Spectrum

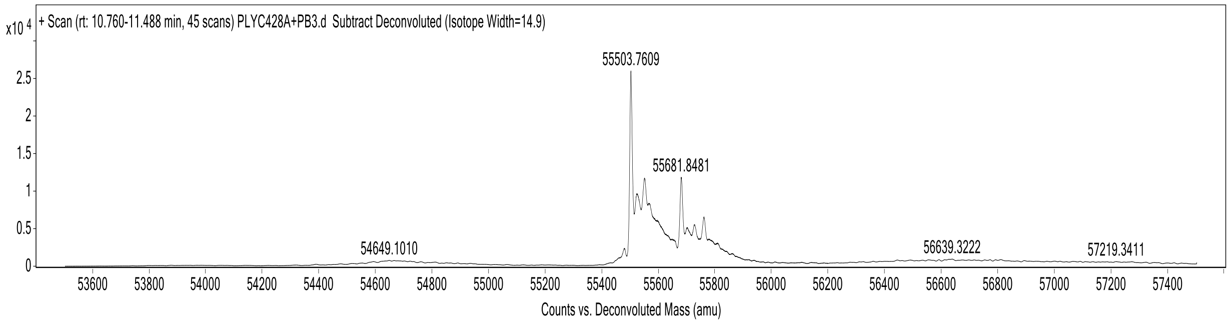

Source Biomolecule Spectrum Peak List

Neutral Mass	Abund
55503.7609	26013.32
55526.1731	9601.33
55551.9334	11702.72
55567.8806	8236.52
55647.6832	3452.26
55681.8481	11836.92
55702.6499	5143.65
55728.5646	5506.06
55761.8676	6537.73
55779.2259	3516.19

Overlaid Deconvolution Plot

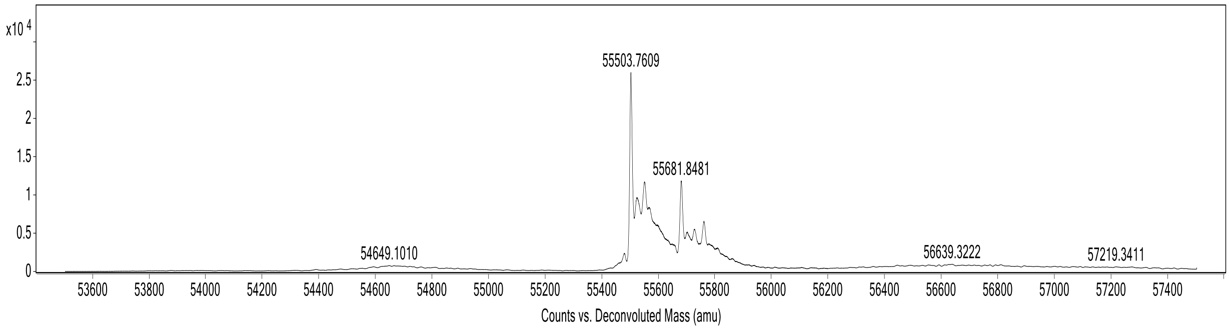

Supplementay Note 2f (report)

**Data File**	Pneumolysin (PLY).d	**Sample Name**	PLY
**Sample Type**	Sample	**Position**	p1a1
**Instrument Name**	Instrument 1	**User Name**	
**Acq Method**	EV68 denat.m	**Acquired Time**	04/03/2019 08:07:52 (UTC+01:00)
**IRM Calibration Status**	Success	**DA Method**	BioConfirmIntactProtein-Default.m
**Comment**	Robertassay_waste		

**Method part to run:**	Acquisition Only	**Sample Group**	
**Info.**		**Stream Name**	LC 1
**Acquisition Time (Local)**	03/03/2019 23:07:52 (UTC-08:00)	**Acquisition SW Version**	6200 series TOF/6500 series Q-TOF B.09.00 (B9044.0)
**QTOF Driver Version**	8.00.00	**QTOF Firmware Version**	14.723
**Tune Mass Range Max.**	10000		

Biomolecules

Mass	Height	Algorithm
55536.39533	236840	Maximum Entropy …
55715.26305	74680	Maximum Entropy …
55572.25221	64701	Maximum Entropy …
55795.67908	50255	Maximum Entropy …

+ Scan (rt: 9.427-9.984 min, 35 scans) Pneumolysin (PLY).d

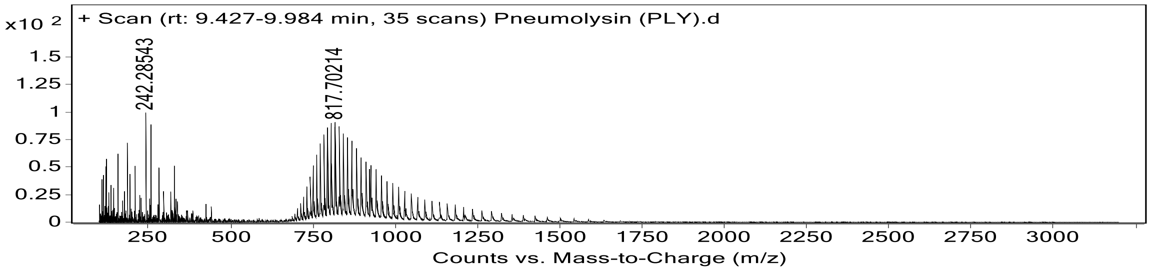

Source Biomolecule Spectrum Peak List

z	Neutral Mass	Abund
	186.22249	9609.53
	242.28543	12792.21
	258.27996	12388.65
2	783.19749	10186.45
	794.37053	10998.36
	805.8649	11535.85
	817.70214	11625.02
	829.89223	11142.63
	842.45352	10277.99
	855.39975	9855.25

+ Scan (rt: 9.427-9.984 min, 35 scans) Pneumolysin (PLY).d Deconvoluted (Isotope Width=0.0)

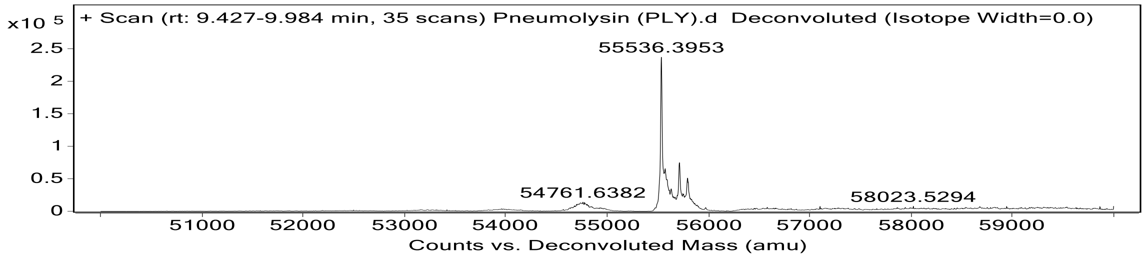

Source Biomolecule Spectrum Peak List

Neutral Mass	Abund
55536.3953	236840.21
55572.2522	64701.08
55591.0409	48287.95
55612.6701	32364.46
55632.3924	34394.94
55654.9567	21759.73
55670.2132	21573.05
55715.263	74680.23
55751.3144	25641.53
55795.6791	50255.13

Supplementary Note 2g (report)

**Data Filename**	PLY- D206R.d	**Sample Name**	PLY- D206R
**Sample Type**	Sample	**Position**	P2-A4
**Instrument Name**	Instrument 1	**User Name**	
**Acq Method**	EV68 denat_waste.m	**Acquired Time**	9/19/2019 3:22:21 PM
**IRM Calibration Status**	Success	**DA Method**	todelete.m
**Comment**			

**Sample Group**		**Info.**	
**Stream Name**	LC 1	**Acquisition SW Version**	6200 series TOF/6500 series Q-TOF B.09.00 (B9044.0)

User Spectra

Fragmentor Voltage	Collision Energy	Ionization Mode
350	0	ESI

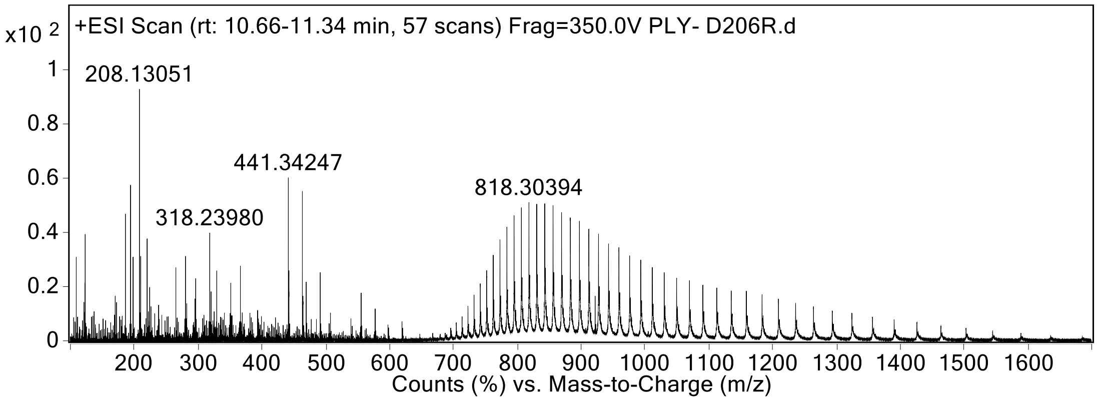

Peak List

m/z	z	Abund
186.14846		5059.38
194.11528		6657.95
208.13051		10688.86
441.34247	1	6579.24
463.32456		5921.64
806.45716		5251.91
818.30394		5450.06
830.49349	2	5388.93
843.07121	2	5406.57
856.01562		5330.69

Fragmentor Voltage	Collision Energy	Ionization Mode
350	0	ESI

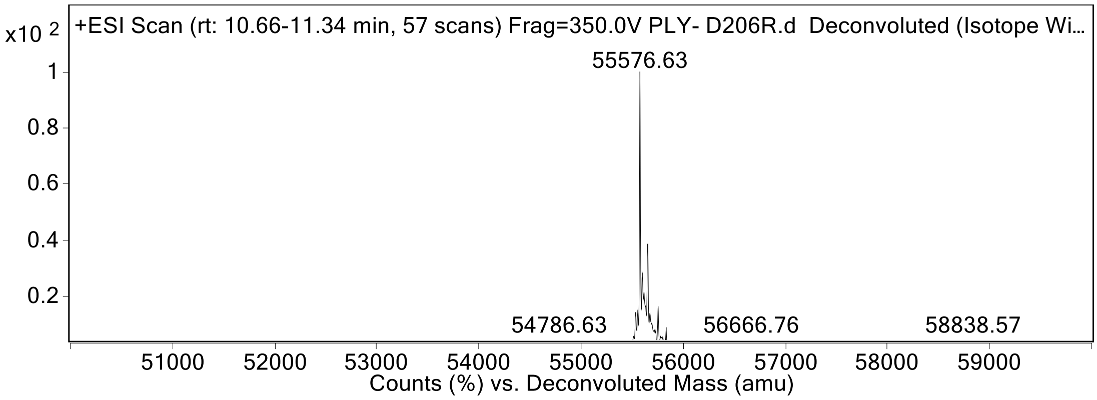

Peak List

m/z	**Abund**	**Score (DB)**
55535.46	34208.97	8
55556.06	35652.12	8
55576.63	239566.63	9
55600.1	67901.87	9
55615.55	51224.46	9
55630.09	39920.42	8
55652.04	92718.63	9
55675.49	33711.67	8
55692.79	24205.11	8
55754.18	39587.54	8

Supplementary Note 2h (report)

**Data Filename**	PLY- CHOL.d	**Sample Name**	PLY- DM
**Sample Type**	Sample	**Position**	P2-A1
**Instrument Name**	Instrument 1	**User Name**	
**Acq Method**	EV68 denat_waste.m	**Acquired Time**	9/19/2019 2:25:15 PM
**IRM Calibration Status**	Success	**DA Method**	todelete.m
**Comment**			

**Sample Group**		**Info.**	
**Stream Name**	LC 1	**Acquisition SW Version**	6200 series TOF/6500 series Q-TOF B.09.00 (B9044.0)

User Spectra

Fragmentor Voltage	Collision Energy	Ionization Mode
350	0	ESI

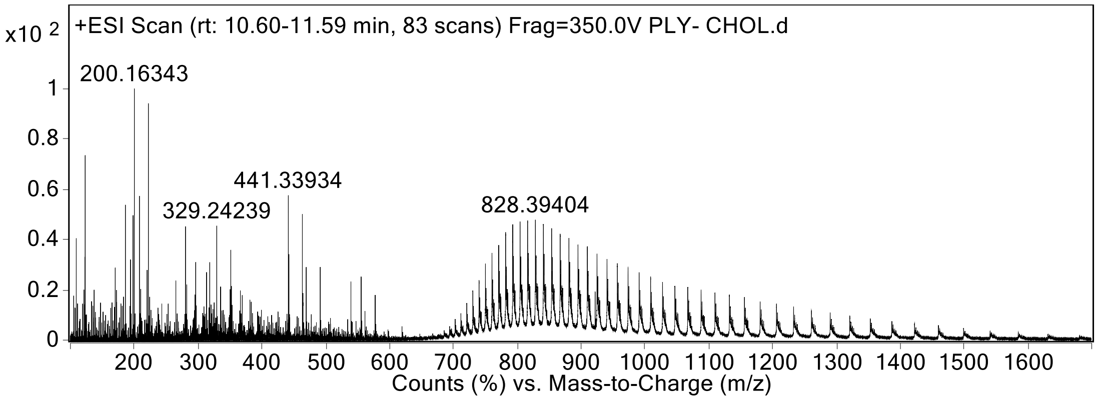

Peak List

m/z	z	Abund
123.07911		6577.9
186.14709		4613.58
198.14753		4200.37
200.16343		8298.9
208.12941		4900.94
222.14512		7799.35
329.24239		4099.54
441.33934	1	5204.9
463.321		4290.76
828.39404		3966.66

Fragmentor Voltage	Collision Energy	Ionization Mode
350	0	ESI

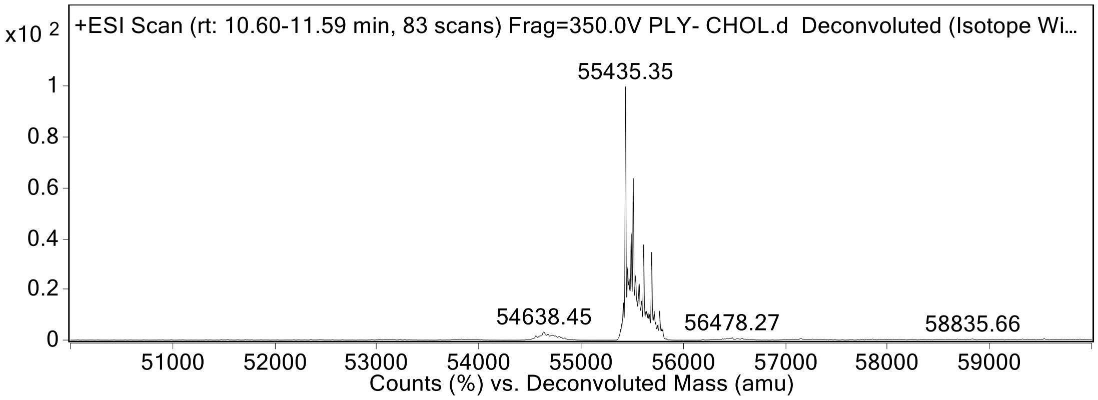

Peak List

m/z	**Abund**	**Score (DB)**
55435.35	162386.57	10
55457.55	45523.72	9
55469.7	38703.31	9
55490.8	67551.34	9
55511.26	102992.82	9
55533.61	40352.45	8
55567.54	36042.41	8
55590.96	24705.13	8
55612.99	61018.96	9
55691.5	56214.55	9

Supplementary Note 2i (report)

**Data Filename**	PLY- E151Q.d	**Sample Name**	PLY- E151Q
**Sample Type**	Sample	**Position**	P2-A3
**Instrument Name**	Instrument 1	**User Name**	
**Acq Method**	EV68 denat_waste.m	**Acquired Time**	9/19/2019 3:03:15 PM
**IRM Calibration Status**	Success	**DA Method**	todelete.m
**Comment**			

**Sample Group**		**Info.**	
**Stream Name**	LC 1	**Acquisition SW Version**	6200 series TOF/6500 series Q-TOF B.09.00 (B9044.0)

User Spectra

Fragmentor Voltage	Collision Energy	Ionization Mode
350	0	ESI

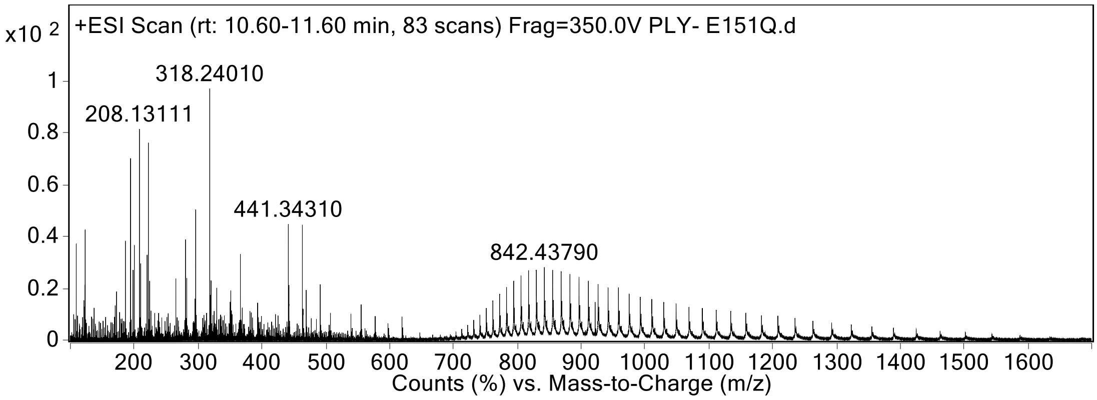

Peak List

m/z	z	Abund
123.08003		5189.03
186.1487		4518.61
194.11581		8426.41
208.13111		9802.98
222.14703		8745.09
280.2635		4464.78
296.2581		5961.11
318.2401	1	11478.98
441.3431	1	5492.6
463.32502	1	5234.45

Fragmentor Voltage	Collision Energy	Ionization Mode
350	0	ESI

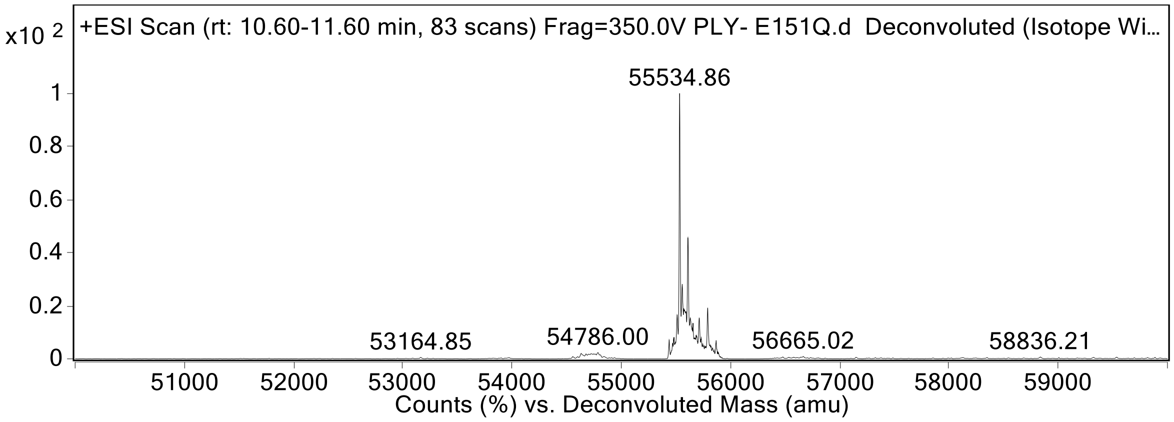

Peak List

m/z	Abund	Score (DB)
55512.34	22368.64	8
55534.86	135490.04	10
55558.31	38122.78	9
55577.99	24839.07	9
55590.49	23926.69	9
55610.73	61763.08	9
55633.12	20624.5	8
55655.95	18186.65	8
55713.72	20856.8	8
55792.37	26026.06	8

**Data Filename**	PLY- E151K.d	**Sample Name**	PLY- E151K
**Sample Type**	Sample	**Position**	P2-A2
**Instrument Name**	Instrument 1	**User Name**	
**Acq Method**	EV68 denat_waste.m	**Acquired Time**	9/19/2019 2:44:20 PM
**IRM Calibration Status**	Success	**DA Method**	todelete.m
**Comment**			

**Sample Group**		**Info.**	
**Stream Name**	LC 1	**Acquisition SW Version**	6200 series TOF/6500 series Q-TOF B.09.00 (B9044.0)

User Spectra

Fragmentor Voltage	Collision Energy	Ionization Mode
350	0	ESI

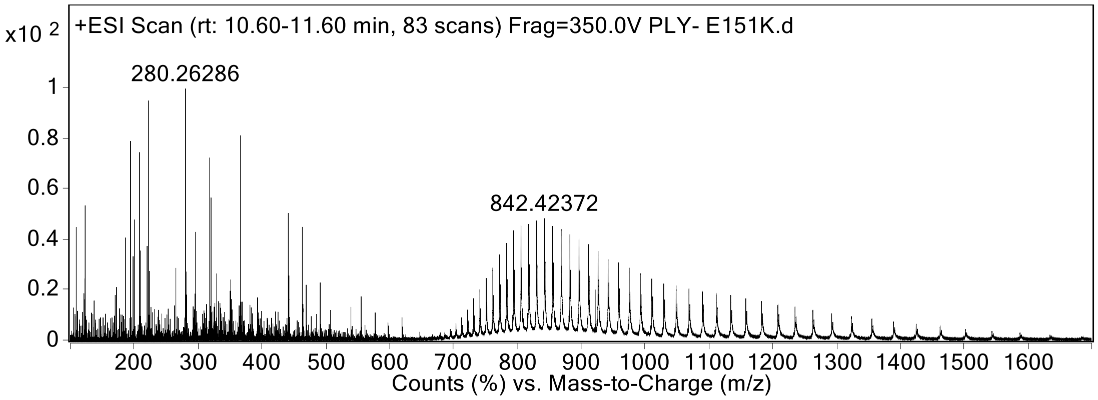

Peak List

m/z	z	Abund
123.07969		4704.57
194.11532		6873.9
208.1304		6569.94
222.14643		7905.31
280.26286		8317.9
318.23921		6177.53
320.25492		4953.28
366.26015		6787.77
441.34164	1	4399.83
842.42372		4021.91

**Fragmentor Voltage**	**Collision Energy**	**Ionization Mode**
350	0	ESI

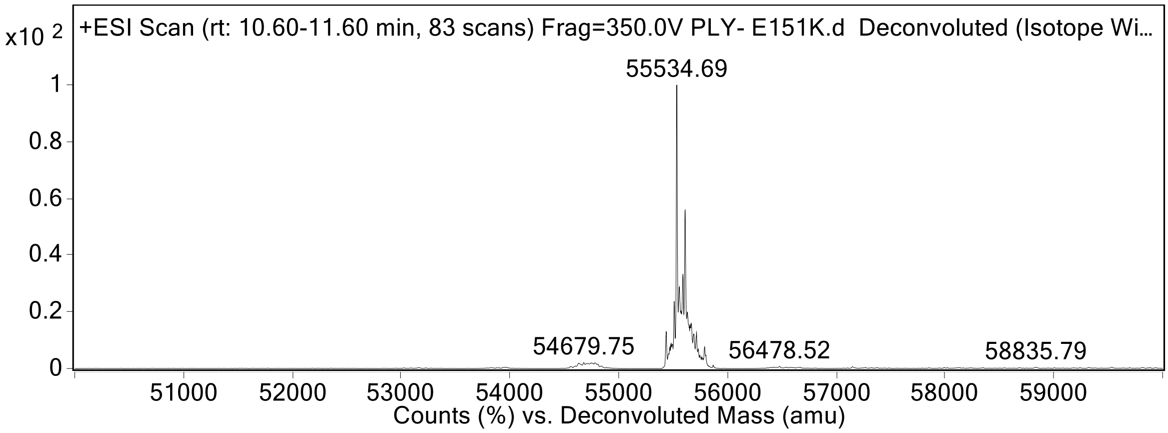

Peak List

m/z	Abund	Score (DB)
55436.74	19725.31	8
55511.72	38204.24	9
55534.69	160997.8	10
55557.51	46248.93	9
55590.09	53461.34	9
55611.01	90112.84	10
	31964.98	9

Supplementary Note 2j (report)

**Data Filename**	PLY- E151Q.d	**Sample Name**	PLY- E151Q
**Sample Type**	Sample	**Position**	P2-A3
**Instrument Name**	Instrument 1	**User Name**	
**Acq Method**	EV68 denat_waste.m	**Acquired Time**	9/19/2019 3:03:15 PM
**IRM Calibration Status**	Success	**DA Method**	todelete.m
**Comment**			

**Sample Group**		**Info.**	
**Stream Name**	LC 1	**Acquisition SW Version**	6200 series TOF/6500 series Q-TOF B.09.00 (B9044.0)

User Spectra

Fragmentor Voltage	Collision Energy	Ionization Mode
350	0	ESI

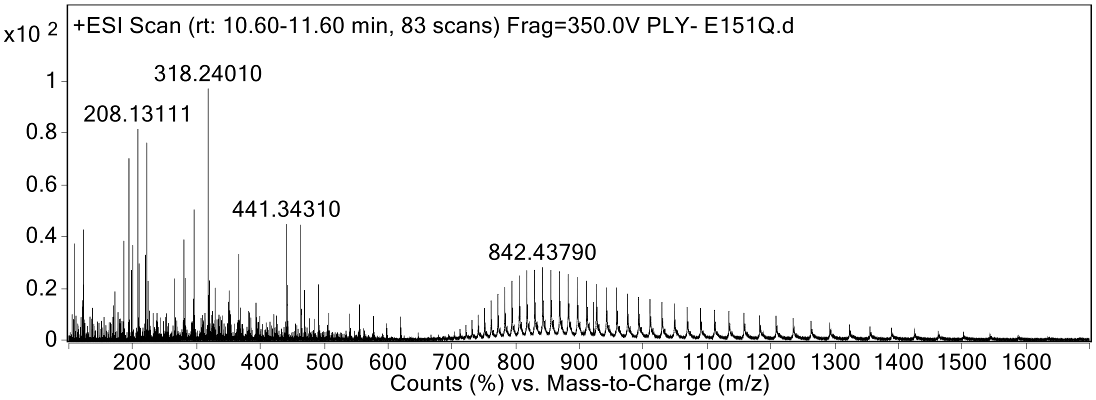

Peak List

m/z	z	Abund
123.08003		5189.03
186.1487		4518.61
194.11581		8426.41
208.13111		9802.98
222.14703		8745.09
280.2635		4464.78
296.2581		5961.11
318.2401	1	11478.98
441.3431	1	5492.6
463.32502	1	5234.45

Fragmentor Voltage	Collision Energy	Ionization Mode
350	0	ESI

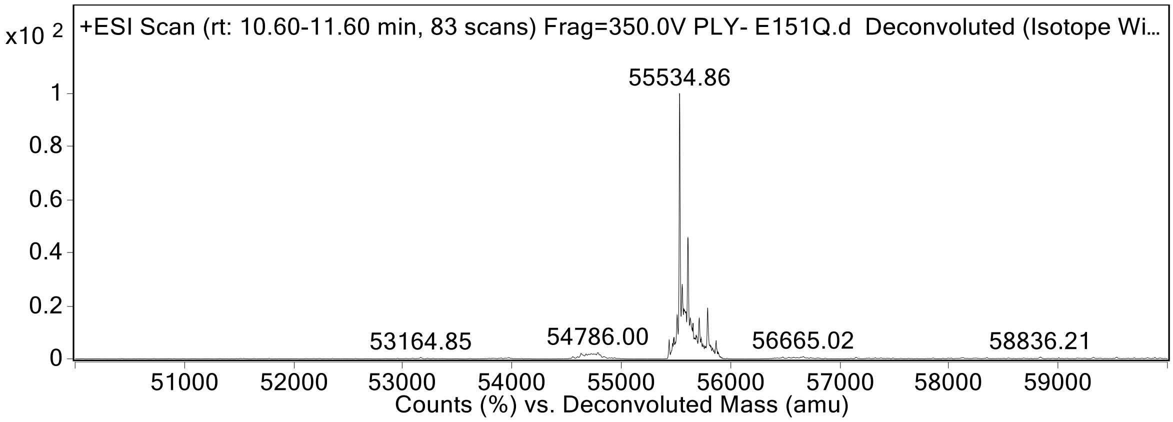

Peak List

m/z	**Abund**	**Score (DB)**
55512.34	22368.64	8
55534.86	135490.0<br>4	10
55558.31	38122.78	9
55577.99	24839.07	9
55590.49	23926.69	9
55610.73	61763.08	9
55633.12	20624.5	8
55655.95	18186.65	8
55713.72	20856.8	8
55792.37	26026.06	8
