# Supplementary material for: Targeted small molecule inhibitors blocking the cytolytic effects of pneumolysin and homologous toxins
Source: Nat Commun. 2024 Apr 26;15:3537. doi: 10.1038/s41467-024-47741-3 (PMC11053136; doi:10.1038/s41467-024-47741-3)
Supplement: Supplementary file 3 — Description of Additional Supplementary Files [file 41467_2024_47741_MOESM3_ESM.pdf]

Description of Additional Supplementary Files

Supplementary Video 1.
The video shows a 3D representation of a cryo-electron tomograms of liposomes treated with PLY. The liposomal membranes contain back-to-back protein pores and prepores of PLY as highlighted (Figure 3a).

Supplementary Video 2.
The video shows a 3D representation of a cryo-electron tomograms of isolated PLY-pores. The protein pores were formed by oligomerization of PLY monomers in liposomal membranes and were isolated by treatment of the PLY-liposome mixture with the detergent Cymal-6 followed by Amphipol-35 (Figure 3b).

Supplementary Video 3.
The video shows a 3D representation of a cryo-electron tomograms of liposomes in the presence of PLY and inhibitor (PB-2). The liposomes are constructed of smooth vesicle membranes without the formation membrane-embedded proteins and pores or pre-pores (Figure 3c).
