# Supplementary material for: Targeted small molecule inhibitors blocking the cytolytic effects of pneumolysin and homologous toxins
Source: Nat Commun. 2024 Apr 26;15:3537. doi: 10.1038/s41467-024-47741-3 (PMC11053136; doi:10.1038/s41467-024-47741-3)
Supplement: Supplementary file 7 — Reporting Summary [file 41467_2024_47741_MOESM7_ESM.pdf]

nature portfolio

Corresponding author(s): Prof. Dr. Jörg Rademann

Last updated by author(s): Mar 5, 2024

Reporting Summary

Nature Portfolio wishes to improve the reproducibility of the work that we publish. This form provides structure for consistency and transparency in reporting. For further information on Nature Portfolio policies, see our Editorial Policies and the Editorial Policy Checklist.

Statistics

For all statistical analyses, confirm that the following items are present in the figure legend, table legend, main text, or Methods section.

n/a	Confirmed	
☐	☒	The exact sample size (*n*) for each experimental group/condition, given as a discrete number and unit of measurement
☐	☒	A statement on whether measurements were taken from distinct samples or whether the same sample was measured repeatedly
☐	☒	The statistical test(s) used AND whether they are one- or two-sided<br>*Only common tests should be described solely by name; describe more complex techniques in the Methods section.*
☐	☒	A description of all covariates tested
☐	☒	A description of any assumptions or corrections, such as tests of normality and adjustment for multiple comparisons
☐	☒	A full description of the statistical parameters including central tendency (e.g. means) or other basic estimates (e.g. regression coefficient) AND variation (e.g. standard deviation) or associated estimates of uncertainty (e.g. confidence intervals)
☐	☒	For null hypothesis testing, the test statistic (e.g. *F*, *t*, *r*) with confidence intervals, effect sizes, degrees of freedom and *P* value noted<br>*Give P values as exact values whenever suitable.*
☒	☐	For Bayesian analysis, information on the choice of priors and Markov chain Monte Carlo settings
☒	☐	For hierarchical and complex designs, identification of the appropriate level for tests and full reporting of outcomes
☒	☐	Estimates of effect sizes (e.g. Cohen's *d*, Pearson's *r*), indicating how they were calculated

Our web collection on statistics for biologists contains articles on many of the points above.

Software and code

Policy information about availability of computer code

Data collection	Molecular Operating Environment (MOE), 2019.0102; Chemical Computing Group Inc. and CCDCs Software Gold V5.1 for Computational methods, Magellan V7.2 for data acquisition in Tecan, Unicorn V6.3 and NanoDrop One Software V1.4.2 for protein purification and concentration measurement, Agilent Mass Hunter workstation data aquisition for Q-TOF/LC-MS, ForteBio Data Acquisition for BLI, Nicomp Z3000 Software ZPW388 V2.16 for DLS, EFI Inspect 3D V4.3 Software for Cryo-TEM and Zen 2012 software for Live Cell microscopy; BD LSRFortessa (BD Biosciences), Data Acquisition: OpenLab CDS. Version 7.2. Agilent Technologies Inc.
Data analysis	Graphpad 5 & 6 & 10, LigandScout 4.4 and Software VMD 1.9.3 for molecular docking analysis, Agilent MassHunter Bio Confirm 10.0 for Q-TOF/LC-MS analysis, ForteBio Data Analysis 9 for BLI, FlowJo V10.8.1, Data Analysis: OpenLab CDS. Version 7.2. Agilent Technologies Inc.

For manuscripts utilizing custom algorithms or software that are central to the research but not yet described in published literature, software must be made available to editors and reviewers. We strongly encourage code deposition in a community repository (e.g. GitHub). See the Nature Portfolio guidelines for submitting code & software for further information.

Data

Policy information about availability of data

All manuscripts must include a data availability statement. This statement should provide the following information, where applicable:

- Accession codes, unique identifiers, or web links for publicly available datasets
- A description of any restrictions on data availability
- For clinical datasets or third party data, please ensure that the statement adheres to our policy

The authors declare that the data supporting the findings of this study are available within the article and its Supplementary Information files. All raw data supporting the findings of this study have been uploaded as a source data file, which is also accessible in figshare via the link https://figshare.com/articles/dataset/_b_A_b_b_Targeted_b_b_Small_Molecule_Inhibitor_with_Elongated_Residence_Time_Blocking_the_Cytolytic_Effects_of_Pneumolysin_and_Homogolous_Toxins_b_/25040261. The MS data has been reposited as a part of the source data file. Crystallographic data of PLY monomers and Cryo-EM map of PLY pre-pore used in this study are available at Protein Data Bank under the following entries: 4ZGH and 2BK2, respectively.

Research involving human participants, their data, or biological material

Policy information about studies with human participants or human data. See also policy information about sex, gender (identity/presentation), and sexual orientation and race, ethnicity and racism.

Reporting on sex and gender	Study participants : two female, one male; no information on gender available
Reporting on race, ethnicity, or other socially relevant groupings	*Please specify the socially constructed or socially relevant categorization variable(s) used in your manuscript and explain why they were used. Please note that such variables should not be used as proxies for other socially constructed/relevant variables (for example, race or ethnicity should not be used as a proxy for socioeconomic status).* *Provide clear definitions of the relevant terms used, how they were provided (by the participants/respondents, the researchers, or third parties), and the method(s) used to classify people into the different categories (e.g. self-report, census or administrative data, social media data, etc.)* *Please provide details about how you controlled for confounding variables in your analyses.*
Population characteristics	Healthy human research participants were randomly chosen from a large healthy cohort irrespective of genetic background, sex and age. Age ranged from 34-35.
Recruitment	Participants were recruited at the University Medical Center Hamburg Eppendorf after providing informed written consent. No self-selective bias was introduced.
Ethics oversight	This study protocol was approved by the ethnical committee of the Ärtzekammer Hamburg (PV4780)

Note that full information on the approval of the study protocol must also be provided in the manuscript.

Field-specific reporting

Please select the one below that is the best fit for your research. If you are not sure, read the appropriate sections before making your selection.

[x] Life sciences   [ ] Behavioural & social sciences   [ ] Ecological, evolutionary & environmental sciences

For a reference copy of the document with all sections, see nature.com/documents/nr-reporting-summary-flat.pdf

Life sciences study design

All studies must disclose on these points even when the disclosure is negative.

Sample size	Minimum triplicate or more sample sizes (n≥ 3) were used for comparisons between two or multiple groups and statistical analysis were followed. There were no calculations for sample size. Sample size was chosen based on previous experience and standards in the field.
Data exclusions	No Data were excluded.
Replication	The experimental findings were successfully repeated thrice in independent experiments, all the essential information for replication of experimental data is available in methods. For functional NK cell assay, one experiment with three independent biological replicates was performed with duplicates for each donor
Randomization	Samples were randolmy allocated
Blinding	Investigators were not blinded because sample preparation, data analysis and evaluation required back and forth transfer of data.

Reporting for specific materials, systems and methods

We require information from authors about some types of materials, experimental systems and methods used in many studies. Here, indicate whether each material, system or method listed is relevant to your study. If you are not sure if a list item applies to your research, read the appropriate section before selecting a response.

Materials & experimental systems

n/a	Involved in the study	
☐	☒	Antibodies
☐	☒	Eukaryotic cell lines
☒	☐	Palaeontology and archaeology
☒	☐	Animals and other organisms
☐	☒	Clinical data
☒	☐	Dual use research of concern
☒	☐	Plants

Methods

n/a	Involved in the study	
☒	☐	ChIP-seq
☐	☒	Flow cytometry
☒	☐	MRI-based neuroimaging

Antibodies

Antibodies used

Following antibodies were used in this study:
αCDS6-BUV395 (BD Bioscience, clone: NCAM16.2, dilution 1:100, cat#: 563554, lot: 2041252)
αCD16-BV711 (Biolegend, clone: 3G8, dilution 1:100, cat# : 302044, lot : B371027)
αCD107a-BV421(Biolegend, clone: H4A3, dilution 1:100, cat#: 328626, lot: B379690)
αCD69-PE (Biolegend, clone: FN50, dilution 1:100, cat# : 310906, lot: B352653)
αPerforin -APC (Biolegend clone:dG9, dilution 1:50, cat# : 308112, lot: B329585)

Following commercial staining kits were used in this study:
Fixable Near-IR Dead Cell Stain (Invitrogen, cat# : L34976, lot: 2298176)
Fluorescent cell tracer dye (CFSE, In vitrogen, cat#: C34554, lot: 2486625)

Validation

All antibodies are commercially available and are validated for flow cytometry by the vendor on their official websites.
Details for antibody validation or access to certificates of analysis of the respective vendors (Lot number of each antibody is provided in “Antibody used”):
Biolegend: https://www.biolegend.com/de-de/certificate-of-analysis?action=Detail
BD Bioscience: https://regdocs.bd.com/regdocs/qcinfo

Eukaryotic cell lines

Policy information about cell lines and Sex and Gender in Research

Cell line source(s)

A549 cells were provided by Prof . Dr. Andreas Hocke, however originally purchased from ATCC (ATCC CCL-185'"). K562 cells were provided by the Altfeld Laboratory, and originally obtained from DSMZ.

Authentication

A549 cells were authenticated by ATCC. No additional authentication procedures were conducted.
Methods used for authentication: https://www.atcc.org/products/crm-ccl-185?matchtype=&network=x&device=c&adposition=&keyword=&gad_source=1&gclid=CjwKCAiAopuvBhBCEiwAm8jaMW076W8zZJKKTvObtMeJjc8eTAuWEDl-gUvzsu137n_v3Bh6OMaLihoC6XEQAvD_BwE
K562 cells were authenticated by DMSZ. No additional authentication procedures were conducted.
Methods used for authentication: DSMZ: https://www.dsmz.de/collection/catalogue/details/culture/ACC-10

Mycoplasma contamination

A549 cells were mycoplasma- free and it was confirmed with MycoAlert Mycoplasma Detect ion Kit (Lonza). K562 were mycoplasma- free as confirmed with HEK -BlueTM 2 cells using the PlasmoTestTM Mycoplasma Detection Kit (InvivoGen).

Commonly misidentified lines
(See ICLAC register)

No, no common misidentified cell lines were used.

Clinical data

Policy information about clinical studies

All manuscripts should comply with the ICMJE guidelines for publication of clinical research and a completed CONSORT checklist must be included with all submissions.

Clinical trial registration: *Provide the trial registration number from ClinicalTrials.gov or an equivalent agency.*

Study protocol: *Note where the full trial protocol can be accessed OR if not available, explain why.*

Data collection: *Describe the settings and locales of data collection, noting the time periods of recruitment and data collection.*

Outcomes: *Describe how you pre-defined primary and secondary outcome measures and how you assessed these measures.*

Plants

Seed stocks: *Report on the source of all seed stocks or other plant material used. If applicable, state the seed stock centre and catalogue number. If plant specimens were collected from the field, describe the collection location, date and sampling procedures.*

Novel plant genotypes: *Describe the methods by which all novel plant genotypes were produced. This includes those generated by transgenic approaches, gene editing, chemical/radiation-based mutagenesis and hybridization. For transgenic lines, describe the transformation method, the number of independent lines analyzed and the generation upon which experiments were performed. For gene-edited lines, describe the editor used, the endogenous sequence targeted for editing, the targeting guide RNA sequence (if applicable) and how the editor was applied.*

Authentication: *Describe any authentication procedures for each seed stock used or novel genotype generated. Describe any experiments used to assess the effect of a mutation and, where applicable, how potential secondary effects (e.g. second site T-DNA insertions, mosiacism, off-target gene editing) were examined.*

Flow Cytometry

Plots

Confirm that:

- [x] The axis labels state the marker and fluorochrome used (e.g. CD4-FITC).
- [x] The axis scales are clearly visible. Include numbers along axes only for bottom left plot of group (a 'group' is an analysis of identical markers).
- [x] All plots are contour plots with outliers or pseudocolor plots.
- [x] A numerical value for number of cells or percentage (with statistics) is provided.

Methodology

Sample preparation: Peripheral blood mononuclear cells (PB M Cs) were obtained from the peripheral blood of healthy donors (n =3) through density gradient centrifugation. NK cells were subsequently enriched from PBMCs using a negative-selection strategy employing the EasySep Human NK Cell Enrichment Kit (Stemcell). NK cells were washed and then resuspended in complete medium (RPMl-1 640 medium (Life Technologies) supplemented with 10% (v/v) heat-inactivated FBS (Biochrom), 100 U/ ml penicillin , and 100µg/ml streptomycin (Sigma-Aldrich) and rested overnight.
After overnight resting, 50.000 NK cells were used against the K562 target cell line in a flow-cytometry based cytotoxicity assay at an effect or to target ratio of 2:1. Target cells were labeled with a fluorescent cell tracer dye (CFSE, lnvitrogen) to measure surviving K562 cells at the end of the assay by flow cytometry. K562 cells were incubated with CFSE (1: 1000) for 8 min (mixing after 4 min) in 1 ml PBS at room temperature. Reaction was quenched usin g 7 ml RPM l/ 10% FCS. NK cell:K562 co-c ultures were incubated for 3 h in 5-ml Polystyrene round-bottom tubes (Corning) at a final volume of 100 µ 1 at 37°C, 5% (v/v) CO2 in RPMl/10%FBS . After incubation, 25 µ1 of precision counting beads (Biolegend) were added to ensure uptake of equal volumes during flow cytometric analysis. CFSE+ cells were immediately counted at a BD LSRFortessa (BD Biosc iences), and analysed by FlowJo Vlü.8.1. Lysis was calculated using K562 without NK cells (K562 only) as a reference by following formula: 100- (K562 survived (absolute count)/ K562 only (absolut e count) x 100). Three individual donors were measured, with two technical replicates for each donor. Mean of the two technical replicates was used for further analysis. Following the initial target cell count , left over samples from each condition were combined for a surface staining of degranulation and activation. Cells were transferred to 96 u-bottom plate (Sarstedt), washed with PBS and stained with fixable Near-lR Dead Cell Stain (lnvitrogen) as well as αCD56-BUV395 (BD Bioscience, clone: NCA M1 6.2, diluti on 1:100), α CD16-BV711 (Biolegend,clone: 3G8, dilution 1:100), αCD107a-BV421(Biolegend, clone: H4A3, dilution 1:100), αCD69-PE (Biolegend, clone: FN50, dilution 1:100) in PBS supplemented with 2% FBS for 30 min at 4°C. Cells were washed twice prior to intracellular staining. Perforin staining was performed using BD Cytofix/Cytoperm Fixation/ Permeabilization kit (BD Biosciences) and αPerforin-APC (Biolegend clone:dG9, dilution 1:50) according to manufacturer's protocol. Cells were fixed with 1xCellFIX (BD Biosciences) and stored at 4°C in PBS until flow cytometry.

Instrument: BD LSR Fortessa v7.0 (BD Biosciences).

Software	FlowJo V10.8.1.
Cell population abundance	50.000 NK cells were used per condition against 25.000 K-562 cells. No further sub-gating for NK cell populations was applied.
Gating strategy	NK cell cyotoxicity against K-562 (Supplemental Figure 16B)<br>First gate was set on population based on forward and side scatter characteristics (FSC-A against SSC-A), second gate was set to exclude doublets usin g FSC- H again st FSC-A. Next , live K-562 cells were gated as CSFE+ (plotted against FSC- H). This last gate was used to export the absolute cell count of CFSE+ K-562.<br><br>NK cell degranulation:<br>First gate was set on population based on forward and side scatter characteristics (FSC-A again st SSC-A), second gate was set to exclude doublets usin g FSC-H against FSC-A, third gate was set on Near-lR Dead Cell Stain negative and CFSE negative cells to gate on live NK cells. Subsequently, an NK cell gate was set gating on CD56(bright and dim) and CD16. Then CD16 was plotted against CD107a to gate the % of CD107a+ cells. This gate was set an each donor individually based an the NK cell only control.

☒ Tick this box to confirm that a figure exemplifying the gating strategy is provided in the Supplementary Information.
